# Optimal HMPAO *α* value for Lassen’s correction algorithm obscured by statistical noise

**DOI:** 10.1007/s12149-016-1073-z

**Published:** 2016-03-26

**Authors:** Masashi Kameyama, Koji Murakami, Masahiro Jinzaki

**Affiliations:** Division of Nuclear Medicine, Department of Radiology, National Center for Global Health and Medicine, 1-21-1 Toyama, Shinjuku-ku, Tokyo, 162-8655 Japan; Division of Nuclear Medicine, Department of Radiology, School of Medicine, Keio University, 35 Shinanomachi, Shinjuku-ku, Tokyo, 160-8582 Japan; Department of Radiology, School of Medicine, Keio University, 35 Shinanomachi, Shinjuku-ku, Tokyo, 160-8582 Japan

**Keywords:** Lassen’s linearization correction algorithm, ^99m^Tc-HMPAO, Single photon emission computed tomography, Image contrast, Renkin–Crone’s equation

## Abstract

**Objective:**

[^99m^Tc] d,l-hexamethyl-propyeneamine oxime (^99m^Tc-HMPAO), a brain perfusion tracer, suffers significant underestimation of regional cerebral blood flow (rCBF). Lassen et al. developed their linearization algorithm to correct the influence of back-diffusion of the tracer, and proposed their parameter *α* as 1.5. Based on mathematical modeling and literature review, recently, a new *α* value of 0.5 has been proposed for Lassen’s correction algorithm for ^99m^Tc-HMPAO, although correction using the old *α* value of 1.5 was confirmed to be sufficient. Inugami et al. reported that linearization correction gives a stable correlation coefficient over a wide range of *α*. Our hypotheses are that statistical noise is the source of the stable correlation coefficient presented by them and that the robustness of the correlation coefficient is the reason why many studies confirmed the value of *α* as 1.5.

**Methods:**

Statistical noise was added in silico to the count, whose relationship with flow was *α* = 0.5. Then, the count was corrected by Lassen’s linearization algorithm with a variety of *α*.

**Results:**

This study confirmed the hypothesis that smaller *α* values (strong correction) increase the noise at high flow values, leading to nominal increases in correlation coefficient as *α* decreases.

**Conclusion:**

Despite this, adoption of the new, smaller *α* value of 0.5 would be more useful clinically in regaining the contrast between low-flow and high-flow areas of the brain.

**Electronic supplementary material:**

The online version of this article (doi:10.1007/s12149-016-1073-z) contains supplementary material, which is available to authorized users.

## Introduction

**Fig. 1:**
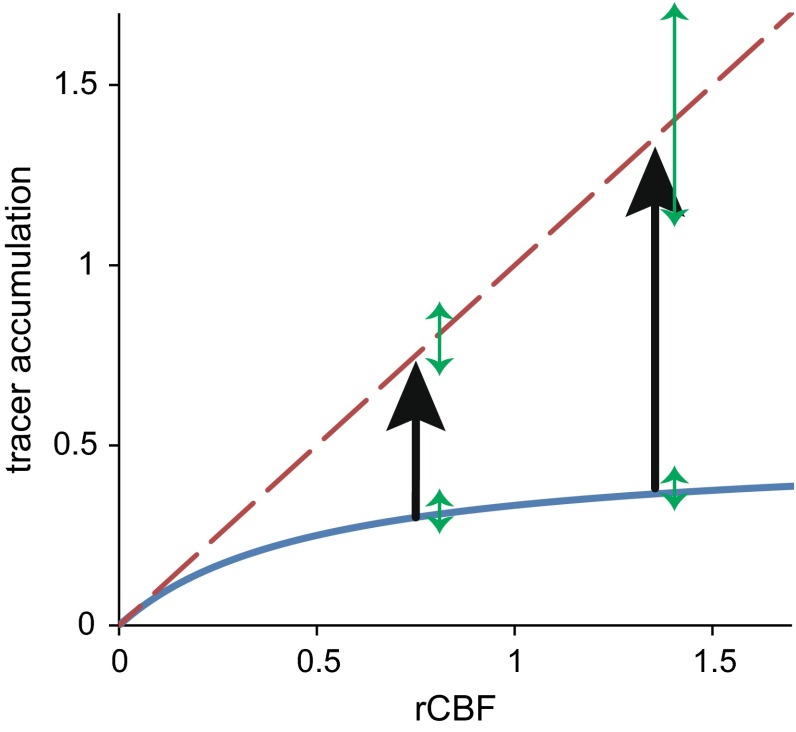
Relationship between rCBF (*X* axis) and SPECT count (*Y* axis) (*blue solid line*) shows significant underestimation at high flow values. Lassen’s linearization algorithm makes this relationship linear (*red broken line*). However, Lassen’s linearization algorithm magnifies noise (*green arrows*) at high flow values

[^99m^Tc] d,l-hexamethyl-propyeneamine oxime (^99m^Tc-HMPAO) is used world-wide to obtain regional cerebral blood flow (rCBF) distribution with single photon emission computed tomography (SPECT). There are several advantages to using ^99m^Tc-HMPAO over [^123^I] *N*-isopropyl-*p*-iodoamphetamine (^123^I-IMP). ^99m^Tc can be easily obtained by ^99^Mo/^99m^Tc generator while ^123^I (half-life: 13 h) needs to be delivered. Furthermore, the image quality afforded by the use of ^99m^Tc-HMPAO is superior due to the ability to administer higher doses of ^99m^Tc and as ^99m^Tc results in less scatter radiation than ^123^I. Despite these advantageous features, the major drawback of using ^99m^Tc-HMPAO is that it suffers underestimation of rCBF at high flow values (Fig. 1). Such underestimation reduces the contrast on SPECT images, complicating the diagnosis of a variety of neurological diseases.

There are two reasons for the low contrast quality in images taken using ^99m^Tc-HMPAO as a tracer. One is the change in first-pass extraction from arterial plasma to brain tissue, and the other is the flow-dependent back-diffusion from brain tissue to arterial plasma. To correct the influence of back-diffusion of ^99m^Tc-HMPAO, Lassen et al. developed their linearization algorithm, and proposed their parameter *α* as 1.5, which was derived from the rate constants they estimated [1]. An author of this paper has recently proposed that mathematical approximation of the Renkin–Crone’s equation [2, 3] leads to Lassen’s equation. It was also proposed that integrating both the back-diffusion and first-pass extraction gives an *α* value of 0.5 which corrects not only for the effect of back-diffusion but also for changes in first-pass extraction fraction depending on rCBF [4]. It is worth noting that a smaller *α* produces stronger compensation.

We would like to explain why this weaker *α* value has been overlooked until recently. Though no studies had confirmed the appropriateness of 1.5 as the optimal *α* value, many studies have confirmed its adequacy [5–8]. Inugami et al. reported that linearization correction gave a stable correlation coefficient over a wide range of *α* (1.0–3.0) when using the cerebellum as the reference region [7]. To explain this stable correlation coefficient, this study hypothesizes that when *α* is small (the correction is strong), the influence of the high flow value greatly increases, therefore the noise associated with the high flow value also increases (Fig. 1). This may explain why the correlation coefficient is so minimally affected by changes in *α* and why an *α* value of 1.5 has been considered acceptable for so long. To confirm this hypothesis, a computer simulation was executed.

## Materials and methods

### Simulation

All simulations were performed with Excel 2010 (Microsoft Corporation, Redmond, WA, USA) on a standard personal computer.

### Data generation

First, *F*/*F*_*r*_ was obtained. *F* is rCBF and *F*_*r*_ is rCBF in a reference region. We generated 1000 points of *F*/*F*_*r*_, which followed a normal distribution with a mean of 1 and standard deviation of 0.18.

Then, SPECT count ratio *C*/*C*_*r*_ was obtained using the following equation which is based on Lassen’s equation:

1 $$\frac{C}{{C_{r}} } = \frac{F}{{F_{r} }}\frac{{1 + \alpha_{\text{t}} }}{{\frac{F}{{F_{r} }} + \alpha_{t} }} + {NL} \times \sqrt {\frac{F}{{F_{r} }}\frac{{1 + \alpha_{t} }}{{\frac{F}{{F_{r} }} + \alpha_{t} }}} \times N(0,1)$$

*α*_*t*_ denotes the true value of Lassen’s parameter, which has been defined as 0.5 for this article, based on our previous paper regarding ^99m^Tc-HMPAO [4]. The second term is statistical noise, where NL is a constant determining noise level. We have adopted the values 0 (no noise level), 0.0125 (extremely low noise level), 0.025 (low noise level) and 0.05 (medium noise level) as NL, and *N*(0, 1) follows a normal distribution with a mean of 0 and a standard deviation of 1. *C/C*_*r*_ follows the normal distribution whose mean is expected count ratio by extended Lassen’s equation (the first term) and whose standard deviation is the square root of the expected count ratio.

### Correction by Lassen’s equation

*C*/*C*_*r*_ is corrected by Lassen’s correction algorithm with a variety of *α*.

2 $$\frac{A}{{A_{r} }} = \frac{{\alpha \times C/C_{r} }}{{1 + \alpha - C/C_{r} }}$$

*A*/*A*_*r*_ is the corrected tracer accumulation ratio. Please note that Eq. (2) is an inverse function of the Lassen’s equation. The correlation coefficient and slope between *A*/*A*_*r*_ and *F*/*F*_*r*_ were determined. The procedure is given in Online Resource 1.

## Results

### Generated *C*/*C*_*r*_ and accumulation corrected by Lassen’s algorithm

**Fig. 2:**
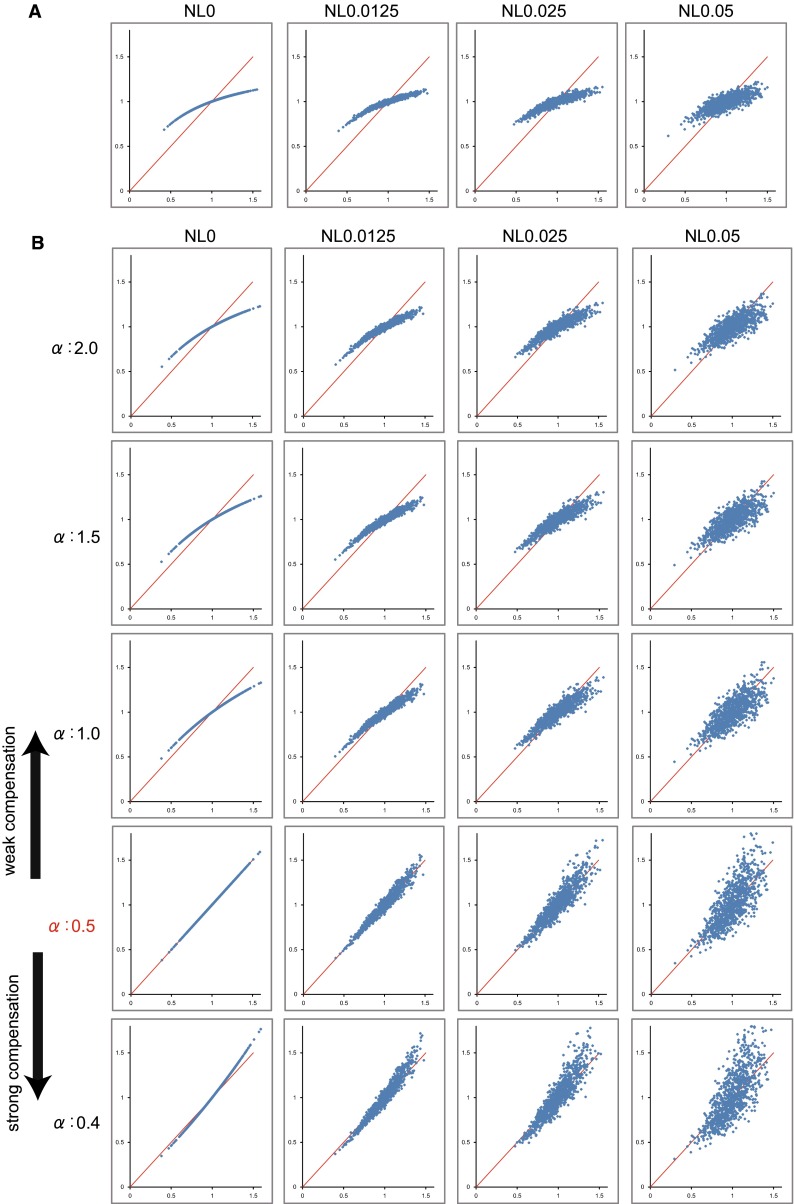
**a** Relationship between rCBF (*X* axis) and SPECT count (*Y* axis), with a variety of statistical noise levels (NL). *Numbers on the axes* are ratios to reference region. *Red lines* indicate identical lines. **b** Relationship between rCBF (*X* axis) and SPECT count corrected by Lassen’s correction algorithm (*Y* axis), with a variety of *α* and noise levels. *α* = 0.5 perfectly matches the true relationship

*F*/*F*_*r*_ was successfully generated and was found to have a distribution between approximately 0.5 and 1.5 (Fig. 2a). It is assumed that most institutions acquire images in medium noise level conditions.

Figure 2b shows the relationship between flow and tracer accumulation corrected by Lassen’s correction algorithm. If the noise is none (NL 0), linearity was regained completely at *α* = 0.5. However, if the noise is low (NL 0.025) or medium (NL 0.05), strong compensation (where *α* is small) exaggerated the noise and plotted points were scattered.

### Correlation coefficient and slope

**Fig. 3:**
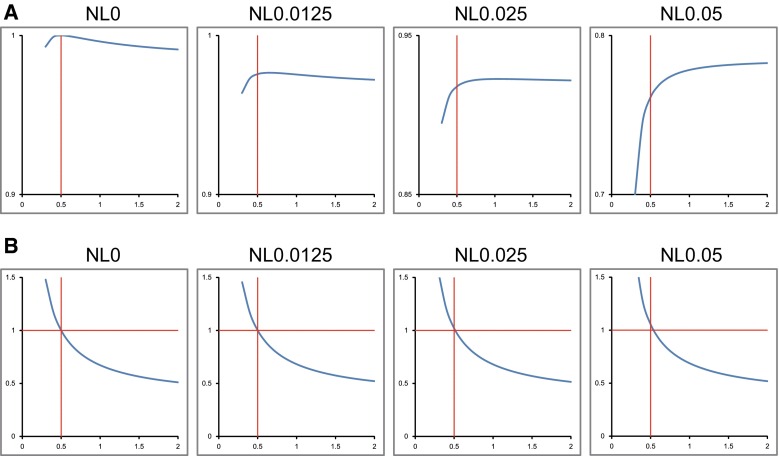
**a** Relationship between *α* of Lassen’s correction algorithm (*X* axis) and correlation coefficient (*Y* axis). The correlation coefficient peaked at the true value in no and very low noise level conditions and showed a stable value over a wide range of *α* in low noise level conditions (NL 0.025). **b** Relationship between *α* of Lassen’s correction algorithm (*X* axis) and slope (*Y* axis). The slope decreased steadily as *α* increased

The correlation coefficient remained stable over a wide range of *α* in low noise level (NL 0.025) conditions. In medium noise level condition (NL 0.05), the correlation coefficient dropped as the true *α* was approached. At no or extremely low noise level (NL 0, NL 0.0125) conditions, the correlation coefficient peaked when *α* = 0.5 (Fig. 3a).

The graph of slope, on the contrary, showed little change across noise levels (Fig. 3b). The slope decreased steadily as *α* increased.

## Discussion

Through computer simulations, this study has revealed the mechanics of the “stable correlation coefficient” presented by Inugami et al. [7] and why the *α* value of 1.5 was reported as sufficient in so many studies.

Although the correlation coefficient is generally stable, a smaller *α* enhances correction and hence the contrast of images. The purpose of Lassen’s correction algorithm is to regain the contrast between low-flow areas and high-flow areas, not to maximize the correlation coefficient. Therefore, from a clinical perspective, abandoning the old *α* value of 1.5 and adopting of the new value of 0.5 would be more useful.

Minimization of noise for images taken using ^99m^Tc-HMPAO is recommended to compensate for poor linearity of rCBF vs. tracer accumulation. The reduction of statistical noise can be achieved by longer acquisition time, higher doses of radio-pharmaceuticals and better SPECT equipment with suitable collimeters.

The slope graph (Fig. 3b) in higher noise conditions tends slightly upward. This is because the greater *C*/*C*_*r*_ (positive noise) is, the greater the effect of linearization correction also. This phenomenon may also augment *α*.

## Electronic supplementary material

Below is the link to the electronic supplementary material.

Online Resource 1. The procedure of the simulation. It was performed with Excel 2010. Simulations on other noise level were also performed (PDF 373 kb)
