# Supplementary material for: Optimal HMPAO α value for Lassen’s correction algorithm obscured by statistical noise
Source: Ann Nucl Med. 2016 Mar 26;30:445–9. doi: 10.1007/s12149-016-1073-z (PMC4925692; doi:10.1007/s12149-016-1073-z)
Supplement: Supplementary file 1 — Online Resource 1. The procedure of the simulation. It was performed with Excel 2010. Simulations on other noise level were also performed (PDF 373 kb) [file 12149_2016_1073_MOESM1_ESM.pdf]

	A	B	C	D	E	F	G	H	I	J	K	L	M	N	O	P	Q	R	S	T	U	V	W	X	Y	Z	AA
1	F/Fr	C/Cr		true_alpha		C/Cr+noise			alpha	0.3	0.4	0.5	0.6	0.7	0.8	0.9	1	1.1	1.2	1.3	1.4	1.5	1.6	1.7	1.8	1.9	2
2				0.5					correlation	0.890157	0.908276	0.91491	0.918024	0.919678	0.920621	0.921181	0.92152	0.921726	0.921849	0.921916	0.921949	0.921957	0.921949	0.921931	0.921905	0.921875	0.921841
3	"1+(NORM.S.INV(RAND()))*$D$9"					"B5+(NORM.S.INV(RAND()))*SQRT(B5)*$D$6"			slope	1.537358	1.201947	1.020698	0.905042	0.824213	0.76431	0.71804	0.681176	0.651089	0.626054	0.604888	0.586754	0.571041	0.557291	0.545157	0.53437	0.524715	0.516023
4		"A5*(1+$D$2)/(A5+$D$2)"																									
5	0.975476	0.991689		noise_level		0.987877				0.949509	0.958819	0.964493	0.968313	0.97106	0.973131	0.974748	0.976045	0.977109	0.977997	0.97875	0.979397	0.979958	0.980449	0.980883	0.981269	0.981614	0.981926
6	0.965463	0.988216		0.025		0.981876		"$F5*J$1/(1+J$1-$F5)"		0.925935	0.939314	0.947529	0.953085	0.957094	0.960123	0.962493	0.964396	0.96596	0.967266	0.968375	0.969327	0.970153	0.970878	0.971518	0.972087	0.972598	0.973057
7	0.851656	0.945125				0.914431				0.711493	0.753287	0.780806	0.800297	0.814826	0.826074	0.835039	0.842352	0.848432	0.853566	0.857959	0.86176	0.865082	0.86801	0.87061	0.872934	0.875024	0.876913
8	1.029989	1.0098		F/Fr SD (range)		0.990121				0.958556	0.966257	0.970937	0.974083	0.976342	0.978043	0.979371	0.980435	0.981308	0.982036	0.982654	0.983183	0.983643	0.984045	0.9844	0.984717	0.985	0.985254
9	1.17392	1.05195		0.18		1.047086				1.242029	1.186791	1.155945	1.136257	1.1226	1.11257	1.104893	1.098826	1.093912	1.089851	1.086438	1.083529	1.081021	1.078836	1.076915	1.075213	1.073695	1.072333
10	1.590273	1.141195				1.150978				2.31707	1.848801	1.648864	1.537982	1.467492	1.418724	1.382978	1.355653	1.334086	1.316631	1.302214	1.290106	1.279792	1.270903	1.263161	1.256358	1.250333	1.244959
11	1.190439	1.056328				1.036093				1.177794	1.138855	1.116704	1.102409	1.09242	1.085046	1.07938	1.074889	1.071243	1.068223	1.065681	1.063511	1.061638	1.060005	1.058568	1.057294	1.056156	1.055135
12	0.96785	0.989049				0.998713				0.994445	0.995509	0.996148	0.996574	0.996879	0.997108	0.997286	0.997429	0.997545	0.997642	0.997725	0.997795	0.997856	0.99791	0.997957	0.997999	0.998036	0.99807
13	0.86129	0.949052				0.959914				0.84677	0.87248	0.888669	0.8998	0.907922	0.914111	0.918983	0.922919	0.926164	0.928885	0.931201	0.933195	0.93493	0.936453	0.937801	0.939003	0.940081	0.941053
14	0.875317	0.954671				0.963175				0.857871	0.881977	0.897103	0.907478	0.915037	0.92079	0.925314	0.928966	0.931975	0.934497	0.936643	0.938489	0.940095	0.941505	0.942753	0.943865	0.944862	0.945761
15	1.277085	1.077961				1.0908				1.564247	1.411127	1.332846	1.285311	1.253382	1.230458	1.213199	1.199737	1.188942	1.180094	1.17271	1.166453	1.161085	1.156428	1.152349	1.148748	1.145545	1.142678
16	0.831281	0.936633				0.942077				0.789621	0.822914	0.844272	0.859138	0.870081	0.878473	0.885113	0.890497	0.894952	0.898698	0.901893	0.904649	0.907051	0.909164	0.911036	0.912707	0.914207	0.915561
17	1.189074	1.05597				1.053445				1.281797	1.215905	1.179524	1.156456	1.140524	1.12886	1.119952	1.112925	1.107242	1.10255	1.098611	1.095256	1.092366	1.089849	1.087638	1.085681	1.083935	1.082369
18	1.218653	1.063612				1.048366				1.249872	1.192567	1.160638	1.140285	1.126179	1.115827	1.107906	1.101649	1.096582	1.092396	1.088878	1.085881	1.083296	1.081045	1.079067	1.077314	1.075751	1.074347
19	1.084527	1.026673				1.066216				1.368209	1.277733	1.228972	1.198481	1.177612	1.162431	1.150892	1.141824	1.13451	1.128487	1.123439	1.119149	1.115457	1.112247	1.10943	1.106937	1.104717	1.102726
20	0.890938	0.960796				0.960019				0.847122	0.872782	0.888937	0.900044	0.908149	0.914324	0.919185	0.923112	0.926349	0.929065	0.931375	0.933364	0.935095	0.936614	0.93796	0.939158	0.940234	0.941204
21	1.125367	1.038566				1.023572				1.110858	1.08767	1.074216	1.06543	1.059242	1.054648	1.051102	1.048283	1.045987	1.044082	1.042475	1.041102	1.039915	1.038878	1.037965	1.037155	1.036431	1.03578
22	1.307708	1.08511				1.074744				1.431365	1.321722	1.263645	1.227681	1.203221	1.185506	1.172085	1.161564	1.153096	1.146133	1.140307	1.13536	1.131107	1.127411	1.124171	1.121306	1.118755	1.116469
23	1.154705	1.046747				1.040649				1.203756	1.158367	1.13274	1.116276	1.104806	1.096357	1.089875	1.084744	1.080581	1.077137	1.074239	1.071768	1.069636	1.067777	1.066142	1.064693	1.0634	1.062239
24	1.405986	1.106503				1.047857				1.246739	1.190261	1.158766	1.138679	1.124752	1.114529	1.106705	1.100524	1.095518	1.091382	1.087905	1.084944	1.08239	1.080165	1.078209	1.076477	1.074932	1.073545
25–270	[illegible]	[illegible]				[illegible]				[illegible]	[illegible]	[illegible]	[illegible]	[illegible]	[illegible]	[illegible]	[illegible]	[illegible]	[illegible]	[illegible]	[illegible]	[illegible]	[illegible]	[illegible]	[illegible]	[illegible]	[illegible]

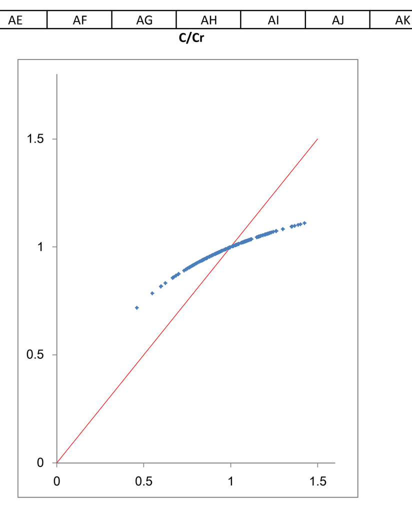

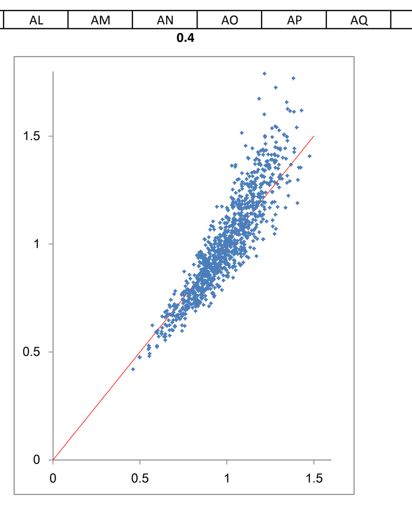

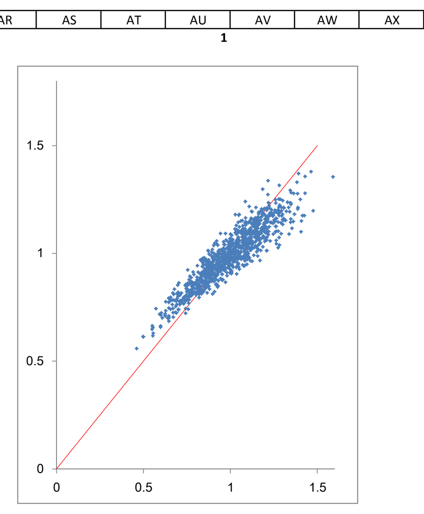

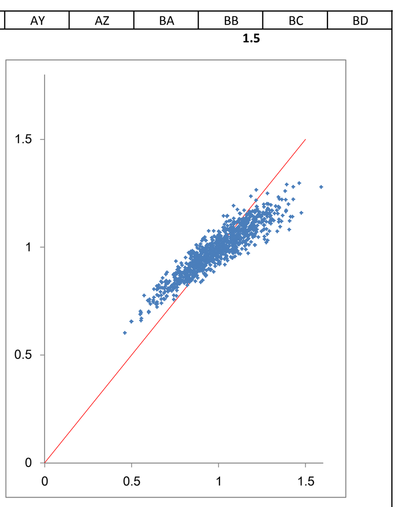

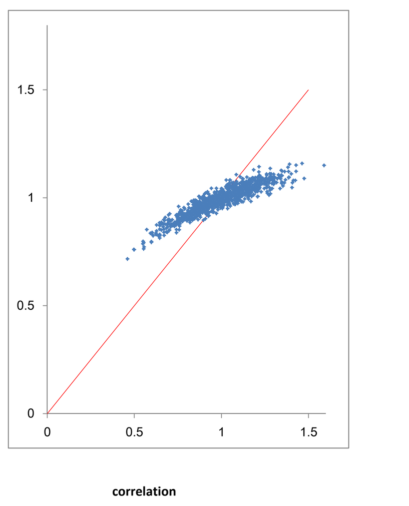

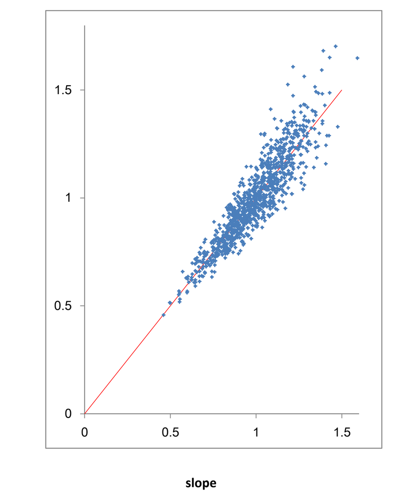

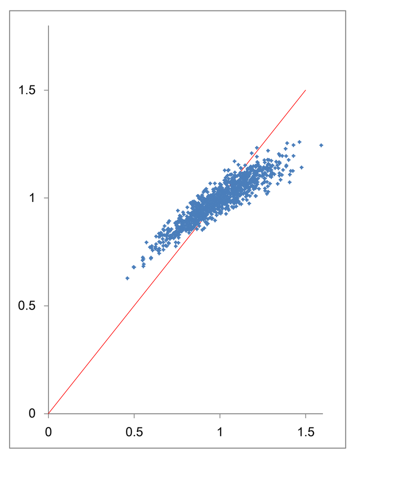

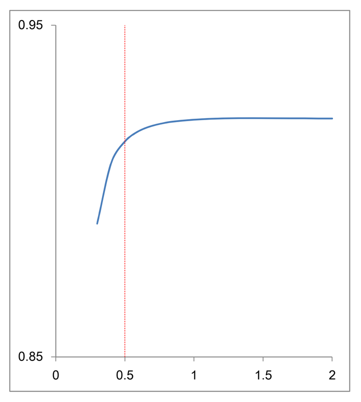

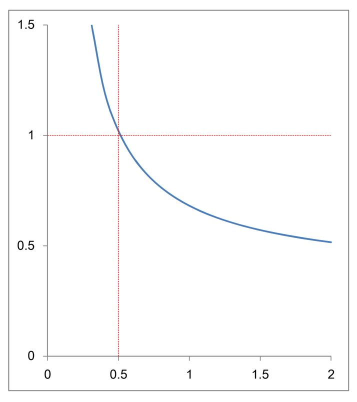

	A	B	C	D	E	F	G	H	I	J	K	L	M	N	O	P	Q	R	S	T	U	V	W	X	Y	Z	AA
271	1.082486	1.026062				1.023429				1.110127	1.087104	1.073743	1.065016	1.05887	1.054306	1.050783	1.047982	1.045702	1.043809	1.042212	1.040848	1.039668	1.038638	1.037731	1.036926	1.036207	1.03556
272	0.950105	0.982796				1.006706				1.029722	1.02387	1.02039	1.018084	1.016443	1.015215	1.014263	1.013502	1.01288	1.012363	1.011925	1.011551	1.011226	1.010943	1.010692	1.01047	1.010271	1.010092
273	1.04668	1.01509				0.996011				0.98294	0.986175	0.988127	0.989432	0.990367	0.991069	0.991615	0.992053	0.992412	0.99271	0.992964	0.993181	0.993369	0.993533	0.993679	0.993808	0.993924	0.994028
274	0.8962	0.962828				0.949447				0.812529	0.842918	0.862267	0.875668	0.885498	0.893017	0.898953	0.90376	0.907731	0.911067	0.913908	0.916358	0.918492	0.920368	0.922029	0.923511	0.92484	0.92604
275	1.068587	1.021863				1.070847				1.401917	1.301335	1.247627	1.214219	1.191431	1.174893	1.162345	1.152497	1.144563	1.138035	1.132569	1.127925	1.123931	1.12046	1.117414	1.114721	1.112323	1.110173
276	1.182726	1.054295				1.083702				1.503067	1.370482	1.301594	1.259391	1.230884	1.210336	1.194823	1.182696	1.172955	1.16496	1.158279	1.152613	1.147748	1.143524	1.139823	1.136553	1.133643	1.131037
277	1.012164	1.004022				1.021963				1.102692	1.081337	1.068917	1.060794	1.055067	1.050812	1.047526	1.044913	1.042784	1.041016	1.039526	1.038251	1.037149	1.036187	1.035339	1.034587	1.033915	1.033311
278	0.813849	0.929158				0.954331				0.828248	0.856539	0.87446	0.886831	0.895883	0.902795	0.908244	0.912652	0.91629	0.919344	0.921944	0.924184	0.926134	0.927848	0.929365	0.930718	0.931931	0.933026
279	0.97929	0.993				0.953747				0.826345	0.854894	0.872991	0.885487	0.894634	0.901619	0.907128	0.911584	0.915262	0.91835	0.92098	0.923245	0.925218	0.926951	0.928485	0.929854	0.931081	0.932189
280	1.292065	1.081488				1.043819				1.222361	1.172234	1.144084	1.126057	1.113524	1.104306	1.097241	1.091654	1.087125	1.08338	1.08023	1.077545	1.075229	1.073211	1.071436	1.069864	1.068461	1.067201
281	0.928707	0.97505				0.918374				0.721942	0.762727	0.789488	0.808396	0.822466	0.833345	0.842007	0.849067	0.854933	0.859883	0.864116	0.867778	0.870977	0.873796	0.876298	0.878534	0.880544	0.882362
282	0.997873	0.99929				1.015884				1.072677	1.057892	1.049214	1.043508	1.03947	1.036462	1.034135	1.03228	1.030768	1.029511	1.028449	1.027542	1.026756	1.02607	1.025465	1.024928	1.024448	1.024016
283	0.705139	0.877665				0.874368				0.616285	0.665385	0.698789	0.722985	0.741321	0.755695	0.767265	0.77678	0.784743	0.791504	0.797316	0.802367	0.806796	0.810712	0.814198	0.817323	0.820139	0.822691
284	1.070764	1.022525				1.042237				1.213015	1.16528	1.138401	1.12116	1.109161	1.100329	1.093557	1.088198	1.083853	1.080258	1.077236	1.074658	1.072434	1.070495	1.068791	1.06728	1.065932	1.064722
285	1.001866	1.000621				1.026242				1.124618	1.098297	1.083088	1.07318	1.066214	1.061048	1.057065	1.053899	1.051324	1.049187	1.047385	1.045846	1.044516	1.043355	1.042333	1.041425	1.040615	1.039887
286	1.322067	1.088379				1.088872				1.54722	1.399902	1.324249	1.278198	1.247219	1.224952	1.208175	1.195081	1.184577	1.175964	1.168773	1.162679	1.157448	1.15291	1.148936	1.145425	1.142303	1.139507
287	0.890942	0.960797				0.953461				0.825415	0.85409	0.872272	0.88483	0.894023	0.901044	0.906582	0.911061	0.91476	0.917864	0.920508	0.922786	0.924769	0.926512	0.928055	0.929431	0.930665	0.931779
288	1.1206	1.037208				1.020245				1.094076	1.074634	1.063297	1.055871	1.05063	1.046733	1.043723	1.041326	1.039374	1.037752	1.036384	1.035215	1.034203	1.033319	1.032541	1.03185	1.031233	1.030678
289	1.003098	1.00103				1.041367				1.207927	1.161484	1.135294	1.11848	1.106772	1.098151	1.091537	1.086304	1.082059	1.078547	1.075593	1.073074	1.0709	1.069005	1.067339	1.065862	1.064544	1.063361
290	0.90478	0.966109				0.937798				0.776746	0.81159	0.834039	0.849708	0.861265	0.870141	0.877173	0.88288	0.887605	0.891582	0.894975	0.897903	0.900457	0.902704	0.904695	0.906473	0.908069	0.909511
291	1.088333	1.027807				1.019023				1.088014	1.069905	1.059326	1.052389	1.047489	1.043844	1.041027	1.038784	1.036956	1.035437	1.034156	1.03306	1.032112	1.031284	1.030555	1.029908	1.029329	1.028809
292	0.752231	0.901069				0.90403				0.684924	0.729101	0.758453	0.77937	0.795032	0.807197	0.81692	0.824868	0.831487	0.837085	0.84188	0.846035	0.849669	0.852874	0.855722	0.85827	0.860563	0.862637
293	0.738517	0.894437				0.879597				0.627682	0.67609	0.708892	0.732588	0.750507	0.764533	0.775809	0.785073	0.792818	0.79939	0.805037	0.809941	0.81424	0.818039	0.82142	0.82445	0.827179	0.829651
294	1.08041	1.02544				1.021695				1.101341	1.080287	1.068037	1.060024	1.054373	1.050175	1.046932	1.044352	1.042251	1.040507	1.039035	1.037777	1.036689	1.035739	1.034902	1.03416	1.033496	1.0329
295	0.998059	0.999352				0.972284				0.890054	0.909279	0.921218	0.929353	0.935253	0.939726	0.943236	0.946062	0.948387	0.950334	0.951987	0.953409	0.954644	0.955728	0.956686	0.95754	0.958304	0.958994
296	0.990152	0.996696				1.008099				1.036071	1.028933	1.024698	1.021894	1.0199	1.01841	1.017254	1.016331	1.015577	1.01495	1.014419	1.013965	1.013572	1.013228	1.012925	1.012656	1.012415	1.012198
297	0.986638	0.995506				1.026106				1.123909	1.097751	1.082633	1.072783	1.065857	1.06072	1.056759	1.053612	1.051051	1.048926	1.047134	1.045604	1.044281	1.043126	1.042109	1.041207	1.040401	1.039677
298	1.083824	1.026462				1.054364				1.287712	1.220199	1.182986	1.159413	1.143142	1.131236	1.122146	1.114978	1.109181	1.104396	1.100379	1.09696	1.094013	1.091448	1.089194	1.087199	1.08542	1.083824
299	0.74145	0.895868				0.87438				0.616309	0.665408	0.69881	0.723006	0.74134	0.755713	0.767283	0.776798	0.78476	0.79152	0.797332	0.802383	0.806812	0.810727	0.814214	0.817338	0.820154	0.822705
300	1.138319	1.042214				1.057227				1.306436	1.233732	1.193869	1.168694	1.151352	1.13868	1.129015	1.121401	1.115246	1.110169	1.105909	1.102284	1.099161	1.096443	1.094055	1.091942	1.090058	1.088368
301	0.951181	0.983179				0.949339				0.812184	0.842618	0.861999	0.875422	0.885269	0.8928	0.898748	0.903563	0.907541	0.910883	0.91373	0.916185	0.918323	0.920202	0.921866	0.923351	0.924683	0.925885
302	0.778336	0.9133				0.921601				0.730658	0.770571	0.796683	0.815096	0.828779	0.839346	0.847753	0.854601	0.860287	0.865083	0.869183	0.872729	0.875825	0.878552	0.880973	0.883136	0.88508	0.886837
303	1.262547	1.074479				1.048884				1.253067	1.194914	1.162543	1.14192	1.127631	1.117147	1.109127	1.102793	1.097664	1.093426	1.089866	1.086833	1.084218	1.08194	1.079938	1.078164	1.076583	1.075163
304	1.186959	1.055413				1.015114				1.068971	1.054978	1.046757	1.041347	1.037516	1.034662	1.032453	1.030693	1.029257	1.028063	1.027055	1.026193	1.025447	1.024795	1.024221	1.02371	1.023254	1.022844
305	0.896612	0.962986				0.937112				0.774713	0.809797	0.832415	0.848209	0.859863	0.868815	0.875908	0.881666	0.886434	0.890447	0.893871	0.896827	0.899405	0.901672	0.903683	0.905477	0.907089	0.908544
306	0.857313	0.947438				0.944771				0.797884	0.83015	0.850794	0.865137	0.875681	0.88376	0.890147	0.895323	0.899604	0.903202	0.906269	0.908915	0.911221	0.913248	0.915043	0.916646	0.918084	0.919383
307	0.827335	0.934958				0.900929				0.67727	0.722085	0.751939	0.773251	0.78923	0.801654	0.81159	0.819719	0.826492	0.832222	0.837133	0.841388	0.845112	0.848397	0.851317	0.853929	0.856281	0.858408
308	0.978467	0.992718				1.011734				1.052915	1.042309	1.036047	1.031914	1.028982	1.026793	1.025098	1.023746	1.022642	1.021724	1.020948	1.020285	1.01971	1.019208	1.018765	1.018372	1.01802	1.017704
309	1.143977	1.043789				1.055857				1.297423	1.227229	1.188644	1.164241	1.147415	1.135111	1.125723	1.118323	1.11234	1.107403	1.10326	1.099734	1.096695	1.094051	1.091728	1.089671	1.087837	1.086192
310	1.236892	1.068194				1.091585				1.571266	1.415735	1.336367	1.288221	1.255902	1.232707	1.21525	1.201637	1.190723	1.181779	1.174315	1.167992	1.162567	1.157862	1.153741	1.150103	1.146867	1.14397
311	1.034092	1.011112				1.047796				1.24637	1.18999	1.158545	1.138489	1.124584	1.114375	1.106563	1.100391	1.095393	1.091262	1.087791	1.084833	1.082283	1.080061	1.078108	1.076378	1.074835	1.07345
312	0.811709	0.928227				0.915253				0.713652	0.755241	0.782605	0.801977	0.816412	0.827583	0.836486	0.843747	0.849783	0.854879	0.859239	0.863011	0.866308	0.869213	0.871793	0.874099	0.876172	0.878047
313	1.207238	1.060694				1.006282				1.027804	1.022338	1.019086	1.016929	1.015395	1.014246	1.013355	1.012644	1.012062	1.011578	1.011168	1.010818	1.010514	1.010249	1.010014	1.009806	1.00962	1.009453
314	1.048633	1.015702				1.019674				1.091237	1.07242	1.061439	1.054242	1.049161	1.045382	1.042462	1.040137	1.038243	1.03667	1.035342	1.034207	1.033226	1.032368	1.031613	1.030942	1.030343	1.029804
315	0.929433	0.975316				0.967784				0.873933	0.895647	0.909201	0.918467	0.925203	0.930319	0.934338	0.937578	0.940246	0.942481	0.94438	0.946014	0.947435	0.948682	0.949784	0.950767	0.951647	0.952441
316	0.753194	0.901529				0.907633				0.693968	0.737364	0.766108	0.786549	0.80183	0.813687	0.823154	0.830887	0.837324	0.842764	0.847423	0.851458	0.854985	0.858096	0.86086	0.863332	0.865555	0.867566
317	1.210058	1.061418				1.048794				1.252508	1.194504	1.162211	1.141634	1.127378	1.116917	1.108914	1.102593	1.097475	1.093247	1.089694	1.086667	1.084057	1.081784	1.079786	1.078016	1.076437	1.075021
318	0.93217	0.976319				1.004223				1.018559	1.014937	1.012776	1.01134	1.010317	1.009551	1.008956	1.008481	1.008092	1.007769	1.007495	1.007261	1.007058	1.00688	1.006723	1.006584	1.006459	1.006347
319	0.921323	0.972323				0.946315				0.802676	0.834337	0.854561	0.868597	0.878909	0.886805	0.893045	0.898101	0.90228	0.905792	0.908786	0.911367	0.913617	0.915594	0.917346	0.918909	0.920311	0.921578
320	0.861261	0.94904				0.939773				0.78265	0.816791	0.838743	0.854045	0.865322	0.873977	0.880829	0.886388	0.890989	0.89486	0.898162	0.901012	0.903496	0.905681	0.907618	0.909347	0.910899	0.9123
321	0.877808	0.955657				0.99866				0.994218	0.995325	0.99599	0.996434	0.996751	0.99699	0.997175	0.997323	0.997445	0.997546	0.997631	0.997705	0.997768	0.997824	0.997873	0.997917	0.997956	0.997991
322	1.088537	1.027868				1.049845				1.259032	1.199291	1.166092	1.144962	1.130332	1.119603	1.111398	1.104919	1.099675	1.095342	1.091703	1.088603	1.08593	1.083602	1.081557	1.079745	1.078129	1.076678
323	0.924616	0.973542				0.977204				0.908193	0.924516	0.934594	0.941436	0.946384	0.95013	0.953064	0.955424	0.957364	0.958986	0.960364	0.961547	0.962575	0.963477	0.964274	0.964983	0.965619	0.966191
324	0.81915	0.931452				0.902568				0.681299	0.725781	0.755372	0.776478	0.79229	0.804578	0.814402	0.822436	0.829128	0.834788	0.839639	0.843841	0.847517	0.85076	0.853643	0.856221	0.858542	0.860641
325	1.120067	1.037056				1.044594				1.226979	1.175661	1.146881	1.128464	1.115668	1.106259	1.09905	1.09335	1.08873	1.08491	1.081699	1.078961	1.0766	1.074542	1.072733	1.07113	1.0697	1.068416
326	1.299458	1.083208				1.083348				1.500126	1.368506	1.300065	1.258119	1.229777	1.209345	1.193916	1.181854	1.172165	1.164211	1.157565	1.151928	1.147087	1.142885	1.139202	1.135948	1.133053	1.13046
327	0.628107	0.83517				0.846647				0.560256	0.612012	0.647924	0.674303	0.694499	0.710458	0.723387	0.734074	0.743056	0.75071	0.757311	0.763062	0.768118	0.772597	0.776592	0.780178	0.783416	0.786352
328	0.913109	0.969255				0.9717				0.887937	0.907494	0.919648	0.927932	0.933942	0.9385	0.942077	0.944957	0.947328	0.949312	0.950997	0.952447	0.953706	0.954812	0.955789	0.956659	0.957439	0.958142
329	1.036065	1.011739				1.048996				1.25376	1.195424	1.162957	1.142274	1.127946	1.117433	1.109391	1.103041	1.097898	1.09365	1.09008	1.087039	1.084417	1.082134	1.080127	1.078349	1.076763	1.07534
330	0.884124	0.958141				0.958863				0.843234	0.869446	0.885969	0.897339	0.90564	0.911967	0.91695	0.920976	0.924296	0.927081	0.929451	0.931492	0.933268	0.934827	0.936208	0.937438	0.938542	0.939537
331	1.362074	1.097223				1.069				1.388312	1.291843	1.240139	1.20791	1.185895	1.169904	1.157762	1.148228	1.140543	1.134217	1.12892	1.124418	1.120545	1.117178	1.114224	1.111612	1.109285	1.107198
332	1.15645	1.047225				1.017901				1.082492	1.065588	1.055696	1.049203	1.044614	1.041199	1.038557	1.036454	1.034739	1.033315	1.032113	1.031084	1.030195	1.029418	1.028733	1.028125	1.027582	1.027094
333	1.049653	1.016021				1.01458				1.066409	1.052961	1.045055	1.039849	1.036162	1.033415	1.031287	1.029592	1.028209	1.027059	1.026088	1.025258	1.024539	1.023911	1.023357	1.022866	1.022426	1.022031
334	0.913347	0.969345				0.965339				0.86536	0.888362	0.902759	0.91262	0.919796	0.925252	0.929541	0.933001	0.935851	0.938239	0.94027	0.942017	0.943537	0.944871	0.946051	0.947102	0.948045	0.948895
335	0.70368	0.876911				0.926771				0.744934	0.783359	0.808377	0.825963	0.839	0.849051	0.857037	0.863535	0.868925	0.873468	0.877349	0.880704	0.883632	0.88621	0.888498	0.890541	0.892377	0.894036
336	1.019625	1.006457				1.02265				1.106163	1.084031	1.071173	1.062768	1.056845	1.052446	1.04905	1.046349	1.044149	1.042323	1.040783	1.039466	1.038328	1.037334	1.036459	1.035682	1.034987	1.034363
337	0.878305	0.955853				0.97797				0.911067	0.926919	0.936699	0.943334	0.948131	0.951761	0.954603	0.956889	0.958768	0.960339	0.961673	0.962819	0.963815	0.964687	0.965459	0.966145	0.96676	0.967315
338	0.646008	0.845555				0.830496				0.530663	0.583311	0.620232	0.647556	0.668596	0.685295	0.698872	0.710126	0.719608	0.727705	0.7347	0.740803	0.746176	0.750941	0.755196	0.75902	0.762473	0.765609
339	1.18553	1.055036				1.129891				1.992651	1.673239	1.526431	1.442081	1.387321	1.348905	1.320466	1.298563	1.281176	1.267039	1.255318	1.245443	1.237009	1.229723	1.223364	1.217767	1.212803	1.20837
340	1.011892	1.003933				1.029337				1.140906	1.110806	1.093497	1.082254	1.074364	1.068521	1.064021	1.060447	1.057542	1.055132	1.053102	1.051368	1.04987	1.048563	1.047412	1.046391	1.04548	1.044661
341	0.846631	0.943055				0.930738				0.756162	0.793364	0.817496	0.834417	0.846938	0.856579	0.86423	0.87045	0.875606	0.87995	0.883659	0.886863	0.889659	0.89212	0.894303	0.896252	0.898003	0.899585
342	0.71026	0.880298				0.893147				0.658578	0.704858	0.735885	0.758133	0.774867	0.78791	0.798362	0.806925	0.81407	0.820121	0.825311	0.829813	0.833755	0.837234	0.840329	0.843099	0.845593	0.84785
343	1.144992	1.044071				1.036618				1.180737	1.141076	1.118534	1.103994	1.093837	1.086342	1.080582	1.076019	1.072314	1.069245	1.066663	1.064459	1.062556	1.060897	1.059438	1.058144	1.056988	1.055951
344	0.882347	0.957444				0.954881				0.830046	0.858091	0.875847	0.888098	0.897061	0.903902	0.909296	0.913658	0.917258	0.92028	0.922852	0.925068	0.926998	0.928693	0.930193	0.931531	0.932732	0.933815
345	0.959298	0.986054				0.979186				0.915657	0.930754	0.940053	0.946357	0.950911	0.954356	0.957052	0.959221	0.961002	0.962491	0.963755	0.964841	0.965785	0.966611	0.967342	0.967993	0.968575	0.9691
346	0.817743	0.930845				0.913442				0.708905	0.750943	0.778647	0.79828	0.812922	0.82426	0.8333	0.840676	0.846808	0.851987	0.85642	0.860256	0.863608	0.866563	0.869187	0.871533	0.873642	0.87555
347	1.144505	1.043936				1.02636				1.125232	1.09877	1.083482	1.073524	1.066523	1.061332	1.057329	1.054148	1.05156	1.049413	1.047603	1.046056	1.04472	1.043553	1.042526	1.041614	1.0408	1.040069
348	1.044163	1.0143				1.036567				1.180453	1.140862	1.118358	1.103841	1.093701	1.086217	1.080467	1.07591	1.072211	1.069147	1.066568	1.064368	1.062468	1.060811	1.059354	1.058062	1.056908	1.055872
349	1.118807	1.036696				1.033649				1.164234	1.128589	1.108231	1.095062	1.085846	1.079035	1.073796	1.069642	1.066266	1.06347	1.061115	1.059105	1.057369	1.055854	1.054522	1.05334	1.052285	1.051337
350	0.968231	0.989181				1.019788				1.091802	1.072861	1.061809	1.054567	1.049454	1.045651	1.042713	1.040374	1.038468	1.036886	1.03555	1.034408	1.03342	1.032558	1.031798	1.031123	1.03052	1.029978
351	0.984995	0.994948				1.011533				1.051976	1.041565	1.035417	1.031358	1.028479	1.02633	1.024664	1.023336	1.022252	1.02135	1.020588	1.019936	1.019371	1.018878	1.018443	1.018056	1.017711	1.0174
352	0.996349	0.99878				0.999025				0.995789	0.996596	0.997081	0.997404	0.997635	0.997809	0.997944	0.998052	0.99814	0.998214	0.998276	0.99833	0.998376	0.998417	0.998452	0.998484	0.998513	0.998538
353	0.623532	0.832462				0.831391				0.532251	0.58486	0.621732	0.64901	0.670007	0.686669	0.700212	0.711437	0.720893	0.728966	0.735941	0.742026	0.747381	0.752131	0.756373	0.760184	0.763626	0.766751
354	1.001061	1.000353				0.985454				0.939881	0.950874	0.957595	0.962128	0.965392	0.967855	0.96978	0.971324	0.972592	0.973651	0.974549	0.97532	0.975989	0.976575	0.977093	0.977554	0.977966	0.978338
355	1.363479	1.097527				1.122335				1.895139	1.616817	1.485885	1.409776	1.360016	1.324943	1.298889	1.278773	1.262772	1.24974	1.238922	1.229797	1.221996	1.215252	1.209362	1.204175	1.199571	1.195458
356	0.912287	0.968947				0.96761				0.873321	0.895128	0.908742	0.918051	0.924818	0.929959	0.933997	0.937253	0.939934	0.942179	0.944088	0.94573	0.947158	0.948411	0.949519	0.950506	0.951392	0.95219
357	1.103281	1.032209				1.015372				1.070207	1.05595	1.047577	1.042068	1.038169	1.035263	1.033015	1.031223	1.029762	1.028547	1.027521	1.026644	1.025884	1.025221	1.024636	1.024117	1.023653	1.023236
358	0.6252	0.833452				0.830643				0.530924	0.583566	0.620479	0.647796	0.668828	0.685521	0.699092	0.710342	0.719819	0.727912	0.734904	0.741005	0.746374	0.751137	0.75539	0.759211	0.762663	0.765797
359	0.920076	0.971859				1.028019				1.133923	1.105453	1.089047	1.078377	1.070883	1.065331	1.061052	1.057653	1.054889	1.052596	1.050664	1.049013	1.047587	1.046342	1.045246	1.044274	1.043406	1.042625
360	1.192145	1.056776				1.04969				1.258067	1.198584	1.165519	1.144471	1.129896	1.119206	1.111031	1.104576	1.09935	1.095033	1.091407	1.088317	1.085654	1.083334	1.081295	1.07949	1.077879	1.076434
361	0.797165	0.921816				0.910733				0.701882	0.744569	0.772767	0.792783	0.807727	0.819311	0.828552	0.836097	0.842372	0.847675	0.852214	0.856143	0.859578	0.862606	0.865296	0.867701	0.869864	0.87182
362	1.08557	1.026984				1.107642				1.72747	1.51546	1.41152	1.349801	1.30892	1.279849	1.258115	1.241253	1.227789	1.21679	1.207636	1.199899	1.193273	1.187535	1.182518	1.178093	1.174163	1.170647
363	1.382832	1.101664				1.141758				2.164581	1.768509	1.593557	1.494963	1.431692	1.387646	1.355217	1.330345	1.310665	1.294704	1.281499	1.270393	1.260922	1.25275	1.245627	1.239364	1.233812	1.228858
364	1.100416	1.031372				1.020412				1.094907	1.075282	1.063841	1.056348	1.05106	1.047128	1.044091	1.041674	1.039704	1.038069	1.036689	1.035509	1.034489	1.033597	1.032812	1.032115	1.031493	1.030933
365	1.078413	1.024839				1.005735				1.025336	1.020365	1.017405	1.015441	1.014043	1.012997	1.012185	1.011536	1.011006	1.010565	1.010192	1.009872	1.009595	1.009353	1.009139	1.00895	1.00878	1.008627
366	1.036081	1.011744				1.043311				1.219349	1.169996	1.142256	1.124482	1.112122	1.103028	1.096057	1.090544	1.086074	1.082377	1.079268	1.076618	1.074332	1.072339	1.070587	1.069034	1.067649	1.066405
367	1.074527	1.023666				1.012292				1.055543	1.044388	1.037807	1.033466	1.030387	1.02809	1.02631	1.024891	1.023733	1.022769	1.021956	1.02126	1.020657	1.02013	1.019666	1.019253	1.018884	1.018553
368	1.129558	1.039753				1.023674				1.111378	1.088073	1.074553	1.065725	1.059507	1.054892	1.051329	1.048497	1.046191	1.044276	1.042662	1.041283	1.04009	1.039049	1.038131	1.037318	1.03659	1.035937
369	1.087509	1.027562				1.046629				1.239242	1.184735	1.154273	1.13482	1.121322	1.111408	1.103817	1.097818	1.092959	1.088942	1.085566	1.082689	1.080208	1.078046	1.076146	1.074462	1.07296	1.071612
370	1.343658	1.0932				1.127825				1.965135	1.657499	1.51518	1.433144	1.379783	1.342299	1.314523	1.293117	1.276115	1.262284	1.250813	1.241146	1.232887	1.225751	1.219522	1.214038	1.209173	1.204828
371	0.873711	0.954034				0.952676				0.822872	0.85189	0.870304	0.883029	0.892349	0.899468	0.905085	0.909629	0.913381	0.916532	0.919214	0.921526	0.923539	0.925308	0.926874	0.928271	0.929524	0.930655
372	1.154832	1.046782				1.048331				1.249657	1.192409	1.16051	1.140175	1.126082	1.115738	1.107823	1.101572	1.09651	1.092326	1.088811	1.085817	1.083234	1.080985	1.079008	1.077257	1.075695	1.074293
373	0.808768	0.926942				0.925352				0.740978	0.779822	0.805147	0.822965	0.836182	0.846377	0.85448	0.861075	0.866547	0.87116	0.875103	0.87851	0.881485	0.884104	0.886429	0.888505	0.890371	0.892057
374	0.939693	0.979056				0.959038				0.843822	0.86995	0.886419	0.897748	0.90602	0.912324	0.917289	0.921299	0.924607	0.927382	0.929742	0.931775	0.933545	0.935098	0.936473	0.937699	0.938798	0.93979
375	0.644682	0.844796				0.883983				0.637461	0.685235	0.717498	0.74075	0.758302	0.772023	0.783042	0.792087	0.799644	0.806053	0.811556	0.816333	0.82052	0.824218	0.827509	0.830456	0.833111	0.835516
376	0.868438	0.95193				0.925971				0.742699	0.781362	0.806554	0.824271	0.83741	0.847542	0.855594	0.862147	0.867583	0.872166	0.876082	0.879466	0.882421	0.885022	0.88733	0.889392	0.891245	0.89292
377	1.29501	1.082175				1.080035				1.473008	1.35019	1.285862	1.246277	1.219462	1.200097	1.185454	1.173995	1.164783	1.157216	1.15089	1.145522	1.14091	1.136905	1.133394	1.130292	1.12753	1.125057
378	0.984216	0.994683				0.973941				0.896101	0.914371	0.925695	0.933401	0.938985	0.943216	0.946534	0.949205	0.951402	0.95324	0.954801	0.956143	0.957309	0.958332	0.959237	0.960042	0.960763	0.961414
379	1.057389	1.018425				1.001126				1.004897	1.003952	1.003385	1.003008	1.002739	1.002537	1.00238	1.002254	1.002152	1.002066	1.001994	1.001932	1.001878	1.001831	1.001789	1.001752	1.001719	1.00169
380	1.014691	1.00485				1.005804				1.025647	1.020613	1.017617	1.015629	1.014213	1.013155	1.012332	1.011676	1.011139	1.010692	1.010315	1.009991	1.009711	1.009466	1.00925	1.009058	1.008886	1.008731
381	1.085729	1.027031				1.034599				1.169477	1.132565	1.111515	1.097911	1.088396	1.081368	1.075964	1.071679	1.068199	1.065316	1.062888	1.060816	1.059027	1.057467	1.056094	1.054876	1.053789	1.052813
382	0.876141	0.954998				0.928075				0.748597	0.786628	0.81136	0.82873	0.8416	0.851517	0.859394	0.865801	0.871115	0.875593	0.879419	0.882724	0.885609	0.888149	0.890402	0.892415	0.894223	0.895857
383	0.550579	0.786108				0.798127				0.477088	0.530428	0.568569	0.597196	0.619476	0.637307	0.651902	0.664069	0.674366	0.683194	0.690847	0.697544	0.703454	0.708708	0.71341	0.717642	0.721471	0.724952
384	0.927388	0.974565				1.018584				1.085849	1.068213	1.057904	1.051141	1.046364	1.042809	1.04006	1.037872	1.036088	1.034607	1.033356	1.032287	1.031362	1.030554	1.029842	1.02921	1.028645	1.028138
385	1.285957	1.080057				1.111602				1.77009	1.541764	1.431011	1.365612	1.322442	1.291814	1.268956	1.251244	1.237116	1.225584	1.215993	1.207891	1.200955	1.194952	1.189705	1.185079	1.18097	1.177297
386	0.962496	0.987178				1.004166				1.018306	1.014734	1.012602	1.011186	1.010178	1.009422	1.008835	1.008366	1.007983	1.007664	1.007394	1.007163	1.006962	1.006787	1.006633	1.006495	1.006372	1.006262
387	0.75419	0.902004				0.925882				0.742451	0.78114	0.806351	0.824083	0.837233	0.847375	0.855434	0.861993	0.867434	0.872021	0.875941	0.879329	0.882286	0.88489	0.887201	0.889265	0.89112	0.892796
388	0.980582	0.993443				0.991634				0.964731	0.971319	0.975315	0.977998	0.979923	0.981372	0.982501	0.983407	0.984149	0.984769	0.985293	0.985744	0.986134	0.986476	0.986778	0.987047	0.987287	0.987503
389	0.967517	0.988933				0.993267				0.971464	0.976825	0.980069	0.982245	0.983804	0.984977	0.985891	0.986624	0.987224	0.987725	0.988149	0.988513	0.988828	0.989105	0.989349	0.989565	0.98976	0.989934
390	0.904629	0.966051				0.971518				0.887278	0.906938	0.919158	0.927489	0.933533	0.938118	0.941715	0.944613	0.946997	0.948993	0.950688	0.952147	0.953414	0.954526	0.955509	0.956384	0.957169	0.957876
391	0.88221	0.957391				0.978401				0.912689	0.928275	0.937885	0.944403	0.949115	0.952679	0.95547	0.957715	0.959559	0.961101	0.96241	0.963535	0.964512	0.965369	0.966126	0.966799	0.967403	0.967947
392	1.014953	1.004935				0.989566				0.956306	0.964409	0.969338	0.972652	0.975032	0.976826	0.978225	0.979347	0.980268	0.981036	0.981687	0.982245	0.98273	0.983155	0.983529	0.983863	0.984161	0.98443
393	1.327874	1.089687				1.10653				1.715816	1.508202	1.406117	1.345407	1.305156	1.276514	1.255091	1.238463	1.225183	1.214332	1.2053	1.197664	1.191123	1.185459	1.180506	1.176138	1.172256	1.168785
394	0.947654	0.981921				0.991938				0.96598	0.972341	0.976199	0.978787	0.980644	0.982042	0.983132	0.984005	0.984721	0.985319	0.985825	0.986259	0.986636	0.986965	0.987256	0.987515	0.987747	0.987956
395	0.896553	0.962964				0.968613				0.876872	0.898138	0.911401	0.920462	0.927045	0.932045	0.935971	0.939136	0.941742	0.943924	0.945778	0.947373	0.94876	0.949977	0.951054	0.952012	0.952872	0.953647
396	0.993754	0.997909				0.976667				0.906187	0.922836	0.933122	0.940108	0.945162	0.948989	0.951986	0.954398	0.956381	0.958039	0.959447	0.960656	0.961707	0.962629	0.963444	0.964169	0.964819	0.965404
397	1.060736	1.019457				1.004264				1.018745	1.015086	1.012903	1.011453	1.01042	1.009646	1.009045	1.008565	1.008173	1.007846	1.007569	1.007333	1.007127	1.006948	1.00679	1.006649	1.006523	1.00641
398	0.819467	0.931589				0.905237				0.687934	0.731854	0.761006	0.781766	0.797301	0.809364	0.819002	0.826879	0.833437	0.838983	0.843733	0.847848	0.851446	0.85462	0.85744	0.859963	0.862232	0.864285
399	0.848165	0.943688				0.981581				0.924802	0.938372	0.946707	0.952346	0.956416	0.95949	0.961896	0.963829	0.965416	0.966743	0.967868	0.968835	0.969674	0.97041	0.97106	0.971639	0.972157	0.972624
400	0.692617	0.871131				0.897532				0.669021	0.714499	0.744879	0.76661	0.782925	0.795624	0.80579	0.814111	0.821049	0.826921	0.831956	0.83632	0.84014	0.843511	0.846508	0.84919	0.851604	0.853789
401	1.11369	1.035227				1.070799				1.40156	1.301086	1.247431	1.214054	1.191286	1.174763	1.162225	1.152386	1.144459	1.137935	1.132474	1.127834	1.123843	1.120374	1.117331	1.11464	1.112243	1.110095
402	1.028274	1.00925				1.002299				1.010041	1.008094	1.00693	1.006155	1.005603	1.005188	1.004867	1.004609	1.004399	1.004224	1.004075	1.003948	1.003838	1.003742	1.003657	1.003581	1.003514	1.003453
403	0.896092	0.962786				1.008938				1.039919	1.031997	1.027301	1.024194	1.021986	1.020337	1.019058	1.018036	1.017202	1.016509	1.015922	1.01542	1.014985	1.014605	1.01427	1.013972	1.013706	1.013467
404	1.164488	1.049411				1.06309				1.346193	1.262163	1.216599	1.188008	1.168395	1.154105	1.14323	1.134676	1.127772	1.122083	1.117313	1.113258	1.109766	1.106729	1.104063	1.101704	1.099602	1.097717
405	0.7903	0.91874				0.92545				0.74125	0.780066	0.80537	0.823172	0.836376	0.846562	0.854656	0.861245	0.866711	0.87132	0.875258	0.878662	0.881633	0.88425	0.886572	0.888646	0.89051	0.892194
406	1.032215	1.010513				1.02323				1.109112	1.086318	1.073085	1.064441	1.058352	1.05383	1.05034	1.047565	1.045305	1.043429	1.041847	1.040495	1.039326	1.038305	1.037406	1.036608	1.035895	1.035254
407	0.744924	0.897554				0.921272				0.729764	0.769767	0.795946	0.814411	0.828134	0.838733	0.847166	0.854036	0.85974	0.864552	0.868666	0.872224	0.87533	0.878067	0.880496	0.882667	0.884618	0.886381
408	1.16781	1.050308				1.021319				1.099449	1.078817	1.066805	1.058945	1.053401	1.049281	1.046098	1.043566	1.041504	1.039791	1.038347	1.037112	1.036044	1.035111	1.034289	1.03356	1.032909	1.032323
409	1.275064	1.07748				1.084797				1.512246	1.376635	1.306347	1.263345	1.234322	1.213415	1.197638	1.185308	1.175408	1.167283	1.160495	1.154739	1.149797	1.145507	1.141749	1.138428	1.135474	1.132828
410	0.783386	0.915608				0.921411				0.730141	0.770106	0.796257	0.8147	0.828406	0.838991	0.847414	0.854274	0.859971	0.864776	0.868884	0.872437	0.875539	0.878272	0.880697	0.882864	0.884813	0.886573
411	0.972981	0.990828				0.976048				0.903882	0.920904	0.931429	0.93858	0.943755	0.947675	0.950745	0.953217	0.955248	0.956947	0.95839	0.95963	0.960707	0.961652	0.962487	0.963231	0.963897	0.964497
412	1.277545	1.07807				1.086811				1.529362	1.388057	1.31515	1.270656	1.240674	1.2191	1.202832	1.190127	1.17993	1.171565	1.164579	1.158657	1.153573	1.149161	1.145296	1.141882	1.138845	1.136125
413	1.027241	1.008918				0.981663				0.925117	0.938634	0.946935	0.952552	0.956604	0.959666	0.962062	0.963986	0.965567	0.966888	0.968009	0.968972	0.969808	0.97054	0.971187	0.971764	0.97228	0.972744
414	1.306209	1.084766				1.071341				1.405593	1.303891	1.249641	1.215914	1.192917	1.176232	1.163574	1.153642	1.145641	1.139058	1.133547	1.128865	1.124838	1.121339	1.118269	1.115554	1.113136	1.110969
415	0.937779	0.978362				1.000801				1.003478	1.002808	1.002406	1.002138	1.001946	1.001803	1.001692	1.001602	1.001529	1.001469	1.001417	1.001373	1.001335	1.001302	1.001272	1.001246	1.001222	1.001201
416	0.860436	0.948706				0.92229				0.732537	0.772259	0.798228	0.816534	0.830133	0.840632	0.848984	0.855786	0.861433	0.866196	0.870268	0.873788	0.876862	0.87957	0.881973	0.88412	0.88605	0.887794
417	1.092558	1.02906				1.030679				1.148085	1.116295	1.098052	1.086218	1.077921	1.07178	1.067052	1.063299	1.060249	1.05772	1.05559	1.05377	1.052199	1.050828	1.049621	1.04855	1.047594	1.046735
418	1.107515	1.033441				1.021857				1.102157	1.080922	1.068569	1.060489	1.054793	1.05056	1.047291	1.044691	1.042573	1.040815	1.039332	1.038064	1.036967	1.03601	1.035167	1.034418	1.033749	1.033148
419	0.905999	0.966572				0.946127				0.80209	0.833825	0.854101	0.868175	0.878515	0.886434	0.892692	0.897762	0.901953	0.905476	0.908479	0.911068	0.913325	0.915308	0.917065	0.918633	0.92004	0.92131
420	0.990651	0.996864				1.017541				1.080729	1.064208	1.054535	1.048183	1.043693	1.040351	1.037766	1.035707	1.034029	1.032635	1.031458	1.030451	1.02958	1.028819	1.028149	1.027554	1.027022	1.026544
421	0.778262	0.913266				0.906009				0.68987	0.733624	0.762645	0.783303	0.798757	0.810754	0.820337	0.828168	0.834688	0.8402	0.84492	0.849009	0.852585	0.855739	0.858541	0.861047	0.863302	0.865342
422	1.031248	1.010203				1.000272				1.00118	1.000953	1.000816	1.000726	1.000661	1.000612	1.000574	1.000544	1.000519	1.000499	1.000481	1.000466	1.000453	1.000442	1.000432	1.000423	1.000415	1.000408
423	0.830091	0.936129				0.948254				0.808755	0.839635	0.859322	0.872967	0.882982	0.890645	0.896698	0.9016	0.905651	0.909054	0.911954	0.914455	0.916633	0.918547	0.920243	0.921756	0.923114	0.924339
424	1.035974	1.01171				0.968288				0.875718	0.89716	0.910537	0.919679	0.926322	0.931368	0.93533	0.938525	0.941155	0.943358	0.94523	0.94684	0.94824	0.949469	0.950556	0.951524	0.952392	0.953174
425	0.928064	0.974813				0.985006				0.938121	0.949419	0.956329	0.960992	0.964351	0.966885	0.968866	0.970456	0.971761	0.972851	0.973775	0.974569	0.975258	0.975862	0.976395	0.976869	0.977294	0.977677
426	0.842596	0.941381				0.975006				0.900023	0.917666	0.928588	0.936015	0.941393	0.945468	0.948661	0.951231	0.953345	0.955113	0.956614	0.957905	0.959026	0.96001	0.960879	0.961653	0.962347	0.962972
427	1.161759	1.048671				1.087104				1.531882	1.389733	1.316439	1.271725	1.241603	1.219931	1.203591	1.190831	1.18059	1.17219	1.165175	1.159228	1.154124	1.149694	1.145813	1.142386	1.139336	1.136606
428	1.174711	1.052162				1.077136				1.449944	1.334475	1.273619	1.236041	1.210529	1.192075	1.178107	1.167166	1.158364	1.15113	1.145079	1.139943	1.135529	1.131695	1.128333	1.125361	1.122716	1.120345
429	0.700644	0.875335				0.851498				0.569561	0.620963	0.656512	0.682562	0.702472	0.718183	0.730898	0.741399	0.750217	0.757728	0.764202	0.769839	0.774793	0.779179	0.783092	0.786602	0.78977	0.792643
430	0.85527	0.946605				0.912009				0.705179	0.747563	0.77553	0.795368	0.81017	0.821639	0.830786	0.838251	0.84446	0.849704	0.854193	0.858079	0.861475	0.864469	0.867127	0.869505	0.871643	0.873576
431	1.110368	1.034268				1.042818				1.216435	1.167827	1.140484	1.122955	1.110761	1.101788	1.094909	1.089466	1.085054	1.081404	1.078335	1.075718	1.07346	1.071492	1.069762	1.068229	1.06686	1.065632
432	1.178564	1.05319				1.054312				1.287378	1.219957	1.182791	1.159247	1.142995	1.131102	1.122022	1.114862	1.109072	1.104292	1.10028	1.096864	1.09392	1.091358	1.089107	1.087114	1.085336	1.083742
433	0.708607	0.879451				0.86558				0.597749	0.647865	0.682182	0.707154	0.72614	0.741063	0.7531	0.763015	0.771324	0.778387	0.784466	0.789752	0.794392	0.798496	0.802153	0.805432	0.808388	0.811068
434	0.890043	0.960448				0.994581				0.976936	0.981288	0.983918	0.98568	0.986942	0.98789	0.988629	0.989221	0.989706	0.990111	0.990453	0.990747	0.991001	0.991224	0.991421	0.991596	0.991753	0.991894
435	0.5718	0.800243				0.85305				0.572581	0.623859	0.659286	0.685226	0.705041	0.720671	0.733316	0.743755	0.75252	0.759984	0.766415	0.772016	0.776936	0.781293	0.785178	0.788664	0.79181	0.794662
436	0.955519	0.98472				1.004401				1.019355	1.015575	1.01332	1.011823	1.010756	1.009957	1.009337	1.008841	1.008436	1.008098	1.007813	1.007568	1.007357	1.007171	1.007008	1.006863	1.006733	1.006616
437	0.970988	0.990139				1.044337				1.225447	1.174526	1.145954	1.127667	1.114958	1.105612	1.098451	1.092789	1.088199	1.084404	1.081213	1.078493	1.076146	1.074102	1.072304	1.070711	1.06929	1.068014
438	1.005235	1.001739				1.031633				1.153235	1.120224	1.101309	1.08905	1.080459	1.074105	1.069214	1.065333	1.062179	1.059564	1.057362	1.055482	1.053858	1.052441	1.051194	1.050087	1.0491	1.048212
439	1.217706	1.063371				1.04122				1.207071	1.160844	1.13477	1.118028	1.106369	1.097783	1.091196	1.085984	1.081756	1.078258	1.075315	1.072806	1.070641	1.068753	1.067094	1.065622	1.06431	1.063131
440	1.219632	1.06386				1.10465				1.696412	1.496053	1.397051	1.338022	1.298823	1.270898	1.249996	1.233762	1.220791	1.210188	1.201359	1.193893	1.187497	1.181957	1.177111	1.172837	1.169039	1.165642
441	0.870289	0.95267				0.980999				0.922568	0.936514	0.945085	0.950887	0.955075	0.958241	0.960717	0.962707	0.964342	0.965709	0.966868	0.967864	0.968729	0.969486	0.970156	0.970752	0.971286	0.971767
442	1.051166	1.016493				1.029819				1.143475	1.112772	1.095129	1.083675	1.075639	1.069689	1.065108	1.06147	1.058513	1.056061	1.053995	1.05223	1.050706	1.049375	1.048205	1.047166	1.046238	1.045405
443	0.871961	0.953337				0.973599				0.894849	0.913317	0.924769	0.932564	0.938213	0.942495	0.945853	0.948556	0.950779	0.95264	0.95422	0.955579	0.956759	0.957795	0.95871	0.959525	0.960256	0.960914
444	0.933125	0.976668				0.981032				0.922694	0.936618	0.945176	0.950969	0.95515	0.958311	0.960783	0.96277	0.964402	0.965767	0.966924	0.967918	0.968782	0.969538	0.970207	0.970802	0.971335	0.971816
445	1.128495	1.039452				1.026824				1.12765	1.100631	1.085033	1.074878	1.067739	1.062447	1.058368	1.055126	1.052489	1.050301	1.048457	1.046882	1.04552	1.044332	1.043285	1.042357	1.041528	1.040783
446	0.94307	0.980275				1.044159				1.224382	1.173735	1.145309	1.127111	1.114463	1.105162	1.098034	1.092397	1.087829	1.08405	1.080874	1.078166	1.07583	1.073794	1.072005	1.070419	1.069004	1.067733
447	1.22213	1.064493				1.099716				1.647237	1.464903	1.373671	1.318911	1.282396	1.256309	1.236742	1.221522	1.209345	1.199381	1.191078	1.184051	1.178029	1.172809	1.168241	1.164211	1.160629	1.157423
448	1.046472	1.015025				0.998886				0.995189	0.99611	0.996664	0.997034	0.997298	0.997496	0.99765	0.997774	0.997875	0.997959	0.99803	0.998091	0.998144	0.99819	0.998231	0.998268	0.9983	0.998329
449	0.866083	0.950985				0.899438				0.673633	0.718743	0.748831	0.770329	0.786456	0.799002	0.80904	0.817254	0.824099	0.829892	0.834858	0.839162	0.842928	0.846251	0.849205	0.851848	0.854227	0.856379
450	0.870896	0.952912				1.006453				1.028577	1.022956	1.019612	1.017395	1.015817	1.014637	1.013721	1.01299	1.012392	1.011894	1.011474	1.011113	1.010801	1.010528	1.010288	1.010074	1.009883	1.009711
451	1.091023	1.028605				1.017183				1.078983	1.062839	1.053383	1.047172	1.04278	1.03951	1.036981	1.034966	1.033324	1.031959	1.030808	1.029822	1.02897	1.028225	1.027569	1.026986	1.026466	1.025998
452	1.083022	1.026222				1.001977				1.008625	1.006955	1.005956	1.00529	1.004816	1.00446	1.004184	1.003962	1.003782	1.003631	1.003504	1.003394	1.0033	1.003217	1.003144	1.003079	1.003021	1.002969
453	0.996198	0.998729				0.997785				0.99047	0.992289	0.993383	0.994114	0.994637	0.995029	0.995334	0.995579	0.995779	0.995946	0.996087	0.996208	0.996313	0.996405	0.996486	0.996558	0.996622	0.99668
454	0.998481	0.999493				1.033625				1.164102	1.128489	1.108148	1.09499	1.085781	1.078976	1.073741	1.06959	1.066217	1.063423	1.06107	1.059062	1.057327	1.055814	1.054482	1.053301	1.052247	1.0513
455	1.045124	1.014602				0.999469				0.997703	0.998144	0.998408	0.998585	0.998711	0.998806	0.998879	0.998938	0.998987	0.999027	0.999061	0.99909	0.999115	0.999137	0.999157	0.999174	0.99919	0.999204
456	0.956616	0.985108				0.987789				0.949154	0.958526	0.964239	0.968086	0.970852	0.972937	0.974565	0.975872	0.976943	0.977838	0.978596	0.979247	0.979812	0.980307	0.980744	0.981132	0.981481	0.981794
457	0.669848	0.858891				0.840795				0.549294	0.601422	0.637734	0.664481	0.685001	0.701243	0.714418	0.72532	0.734491	0.742312	0.749061	0.754944	0.760119	0.764705	0.768797	0.772472	0.77579	0.778801
458	1.209383	1.061245				1.006474				1.028672	1.023031	1.019676	1.017452	1.015869	1.014685	1.013766	1.013032	1.012432	1.011933	1.011511	1.01115	1.010837	1.010563	1.010321	1.010107	1.009915	1.009742
459	0.833571	0.9376				0.907631				0.693962	0.737358	0.766102	0.786544	0.801825	0.813682	0.823149	0.830883	0.83732	0.84276	0.847419	0.851454	0.854982	0.858093	0.860856	0.863328	0.865552	0.867563
460	1.17135	1.051261				1.045392				1.231764	1.179207	1.149771	1.130951	1.117881	1.108274	1.100916	1.0951	1.090386	1.086489	1.083214	1.080422	1.078013	1.075915	1.07407	1.072436	1.070978	1.069668
461	0.746807	0.898463				0.89359				0.659622	0.705823	0.736787	0.758984	0.775676	0.788685	0.799108	0.807648	0.814772	0.820805	0.82598	0.830468	0.834397	0.837866	0.840951	0.843712	0.846198	0.848448
462	1.123848	1.038134				1.088376				1.542892	1.397038	1.322052	1.276378	1.24564	1.223541	1.206887	1.193887	1.183457	1.174904	1.167763	1.16171	1.156515	1.152007	1.148059	1.144572	1.14147	1.138693
463	1.044823	1.014507				1.005576				1.02462	1.019792	1.016916	1.015009	1.01365	1.012634	1.011845	1.011214	1.010699	1.01027	1.009908	1.009597	1.009328	1.009093	1.008885	1.008701	1.008536	1.008387
464	1.045071	1.014585				1.067441				1.376994	1.283912	1.233867	1.202617	1.181247	1.165712	1.153908	1.144636	1.13716	1.131004	1.125847	1.121464	1.117693	1.114414	1.111537	1.108992	1.106724	1.104692
465	1.167513	1.050228				1.042802				1.216344	1.16776	1.140429	1.122908	1.110719	1.101749	1.094873	1.089433	1.085022	1.081374	1.078306	1.07569	1.073433	1.071466	1.069736	1.068203	1.066836	1.065608
466	0.925501	0.973869				0.915616				0.714609	0.756107	0.783402	0.802721	0.817113	0.828251	0.837126	0.844364	0.85038	0.855459	0.859805	0.863565	0.86685	0.869745	0.872316	0.874614	0.87668	0.878548
467	0.809345	0.927195				0.932813				0.76213	0.798665	0.822316	0.838878	0.851122	0.860542	0.868015	0.874087	0.879118	0.883355	0.886973	0.890097	0.892823	0.895222	0.897349	0.899248	0.900954	0.902496
468	1.094913	1.029755				0.974203				0.897065	0.915181	0.926406	0.934044	0.939577	0.94377	0.947057	0.949704	0.95188	0.953701	0.955247	0.956577	0.957732	0.958745	0.959641	0.960438	0.961153	0.961797
469	0.826792	0.934727				0.930445				0.755323	0.792618	0.816817	0.833788	0.846348	0.856019	0.863696	0.869936	0.87511	0.879469	0.883191	0.886406	0.889212	0.891682	0.893872	0.895829	0.897586	0.899174
470	0.697359	0.873621				0.889037				0.64899	0.695969	0.72757	0.750281	0.767391	0.780744	0.791456	0.80024	0.807572	0.813786	0.81912	0.823747	0.8278	0.831379	0.834563	0.837413	0.83998	0.842304
471	0.988941	0.996286				0.991464				0.964032	0.970747	0.974821	0.977556	0.979518	0.980996	0.982148	0.983072	0.983829	0.98446	0.984996	0.985455	0.985853	0.986202	0.98651	0.986784	0.987029	0.98725
472	1.191502	1.056607				1.063131				1.346478	1.262366	1.216761	1.188145	1.168515	1.154214	1.14333	1.13477	1.12786	1.122167	1.117394	1.113335	1.109841	1.106801	1.104134	1.101773	1.099669	1.097783
473	1.129822	1.039827				1.059748				1.323293	1.245837	1.203569	1.176947	1.158642	1.145283	1.135103	1.127089	1.120615	1.115277	1.1108	1.106991	1.10371	1.100856	1.09835	1.096132	1.094155	1.092381
474	1.234959	1.067713				1.066877				1.372934	1.281059	1.231608	1.200709	1.179571	1.164199	1.152517	1.143339	1.135938	1.129843	1.124737	1.120397	1.116662	1.113415	1.110565	1.108045	1.105799	1.103785
475	1.103121	1.032163				0.990533				0.960232	0.967632	0.972127	0.975147	0.977316	0.978949	0.980222	0.981244	0.982081	0.98278	0.983372	0.98388	0.984321	0.984707	0.985048	0.985351	0.985622	0.985867
476	1.246481	1.070565				1.04309				1.218041	1.169023	1.141461	1.123797	1.111511	1.102472	1.095542	1.090061	1.085617	1.081941	1.07885	1.076214	1.073941	1.071959	1.070217	1.068673	1.067295	1.066058
477	1.04608	1.014902				1.015423				1.070454	1.056145	1.047741	1.042213	1.038299	1.035383	1.033127	1.031329	1.029862	1.028643	1.027614	1.026734	1.025972	1.025306	1.024719	1.024198	1.023733	1.023314
478	0.916058	0.97036				0.936438				0.772721	0.808038	0.830822	0.846738	0.858486	0.867513	0.874666	0.880474	0.885284	0.889332	0.892787	0.895769	0.89837	0.900659	0.902688	0.904499	0.906125	0.907594
479	1.075063	1.023829				1.035264				1.173167	1.135358	1.11382	1.09991	1.090185	1.083003	1.077483	1.073106	1.069552	1.066608	1.06413	1.062015	1.060189	1.058596	1.057194	1.055952	1.054842	1.053846
480	1.407932	1.106904				1.070163				1.396852	1.297806	1.244846	1.211877	1.189377	1.173042	1.160645	1.150914	1.143073	1.136619	1.131216	1.126625	1.122676	1.119243	1.116232	1.113569	1.111197	1.10907
481	0.847628	0.943467				0.973493				0.894463	0.912993	0.924484	0.932306	0.937976	0.942273	0.945643	0.948356	0.950587	0.952455	0.954041	0.955405	0.95659	0.957629	0.958548	0.959366	0.960099	0.96076
482	1.214843	1.062642				1.078147				1.45792	1.339924	1.27787	1.239598	1.213635	1.194866	1.180664	1.169543	1.160599	1.153249	1.147103	1.141886	1.137403	1.133509	1.130096	1.127079	1.124393	1.121987
483	1.226059	1.065484				1.094767				1.600283	1.434667	1.350789	1.300115	1.266186	1.24188	1.22361	1.209377	1.197976	1.188638	1.180849	1.174254	1.168597	1.163693	1.159399	1.155609	1.152238	1.149222
484	0.999449	0.999816				0.996084				0.98325	0.986428	0.988344	0.989626	0.990543	0.991232	0.991769	0.992199	0.992551	0.992844	0.993093	0.993306	0.993491	0.993652	0.993795	0.993922	0.994036	0.994138
485	0.890874	0.960771				0.93401				0.765602	0.801741	0.825111	0.841462	0.853544	0.862836	0.870204	0.876189	0.881148	0.885324	0.888888	0.891966	0.894651	0.897013	0.899108	0.900979	0.902659	0.904176
486	1.148185	1.044954				1.053239				1.280475	1.214944	1.178749	1.155794	1.139937	1.128327	1.11946	1.112465	1.106807	1.102136	1.098214	1.094874	1.091996	1.089491	1.087289	1.08534	1.083602	1.082042
487	0.888787	0.95996				0.95849				0.841988	0.868375	0.885017	0.89647	0.904834	0.91121	0.916232	0.920289	0.923636	0.926443	0.928832	0.93089	0.93268	0.934252	0.935644	0.936885	0.937998	0.939001
488	1.13899	1.042401				1.059975				1.324831	1.246938	1.204449	1.177696	1.159303	1.14588	1.135654	1.127603	1.121101	1.115739	1.111242	1.107416	1.104122	1.101255	1.098738	1.09651	1.094525	1.092744
489	1.158693	1.047837				1.062763				1.343927	1.260554	1.215317	1.186921	1.167437	1.153239	1.142433	1.133932	1.127071	1.121416	1.116675	1.112644	1.109173	1.106154	1.103504	1.101159	1.099069	1.097195
490	1.180678	1.053752				1.056855				1.303984	1.231965	1.19245	1.167485	1.150284	1.137712	1.128122	1.120566	1.114458	1.109419	1.105191	1.101592	1.098492	1.095794	1.093424	1.091326	1.089456	1.087778
491	1.191201	1.056528				1.053709				1.283495	1.217139	1.180519	1.157306	1.141277	1.129543	1.120582	1.113516	1.1078	1.103081	1.099119	1.095746	1.09284	1.090309	1.088086	1.086118	1.084362	1.082787
492	0.87022	0.952642				0.945179				0.799147	0.831255	0.851788	0.86605	0.876534	0.884564	0.890912	0.896057	0.900311	0.903886	0.906934	0.909563	0.911854	0.913868	0.915652	0.917244	0.918673	0.919963
493	1.259547	1.073754				1.059049				1.318588	1.242466	1.20087	1.174653	1.156617	1.143449	1.133413	1.12551	1.119125	1.11386	1.109443	1.105685	1.102449	1.099632	1.097159	1.09497	1.093019	1.091269
494	0.908135	0.96738				0.964207				0.861431	0.885015	0.899795	0.909926	0.917303	0.922915	0.927328	0.930888	0.933822	0.936281	0.938371	0.940171	0.941736	0.94311	0.944325	0.945408	0.946379	0.947255
495	1.00761	1.002524				0.972537				0.890976	0.910056	0.921902	0.929972	0.935823	0.94026	0.94374	0.946543	0.948849	0.950778	0.952418	0.953827	0.955052	0.956126	0.957076	0.957922	0.958681	0.959364
496	0.657315	0.851948				0.879595				0.627677	0.676085	0.708888	0.732584	0.750503	0.764529	0.775805	0.785069	0.792814	0.799387	0.805033	0.809938	0.814236	0.818035	0.821417	0.824446	0.827176	0.829648
497	1.17466	1.052148				1.00675				1.029924	1.024031	1.020528	1.018205	1.016553	1.015317	1.014358	1.013592	1.012966	1.012445	1.012005	1.011628	1.011301	1.011016	1.010764	1.01054	1.01034	1.01016
498	0.778291	0.913279				0.92953				0.752717	0.790299	0.814706	0.831832	0.844512	0.854279	0.862033	0.868338	0.873566	0.877971	0.881733	0.884984	0.88782	0.890317	0.892532	0.89451	0.896287	0.897893
499	0.992279	0.997413				0.980784				0.921744	0.935827	0.944486	0.950348	0.95458	0.957778	0.960281	0.962293	0.963945	0.965326	0.966498	0.967505	0.968379	0.969145	0.969822	0.970424	0.970964	0.971451
500	0.964085	0.987735				1.009592				1.042938	1.034396	1.029339	1.025994	1.023618	1.021844	1.020468	1.01937	1.018473	1.017727	1.017096	1.016557	1.016089	1.015681	1.015321	1.015001	1.014715	1.014457
501	1.059327	1.019023				1.046322				1.23738	1.18336	1.153154	1.133859	1.120467	1.11063	1.103097	1.097143	1.09232	1.088333	1.084982	1.082126	1.079663	1.077517	1.075631	1.073959	1.072468	1.07113
502	0.933436	0.976782				0.983579				0.932536	0.944794	0.952304	0.957378	0.961035	0.963796	0.965955	0.967689	0.969112	0.970302	0.97131	0.972176	0.972928	0.973587	0.974169	0.974688	0.975152	0.975569
503	1.109904	1.034134				1.043239				1.21892	1.169676	1.141995	1.124257	1.111921	1.102845	1.095888	1.090385	1.085924	1.082234	1.079131	1.076485	1.074203	1.072214	1.070465	1.068915	1.067533	1.066291
504	0.754545	0.902174				0.960297				0.848062	0.873587	0.889653	0.900696	0.908754	0.914892	0.919724	0.923626	0.926844	0.929542	0.931838	0.933815	0.935535	0.937045	0.938381	0.939573	0.940641	0.941605
505	1.033888	1.011046				1.021722				1.101474	1.080391	1.068124	1.0601	1.054442	1.050238	1.046991	1.044408	1.042304	1.040557	1.039084	1.037824	1.036735	1.035783	1.034946	1.034202	1.033537	1.03294
506	0.951494	0.983291				0.985592				0.940425	0.951324	0.957986	0.962479	0.965714	0.968155	0.970062	0.971593	0.972849	0.973898	0.974788	0.975552	0.976215	0.976795	0.977308	0.977765	0.978174	0.978542
507	1.078037	1.024726				0.969793				0.881079	0.9017	0.914543	0.92331	0.929676	0.934508	0.938301	0.941358	0.943874	0.945981	0.947771	0.949311	0.950649	0.951824	0.952862	0.953787	0.954617	0.955364
508	0.87419	0.954224				0.981568				0.92475	0.938329	0.946669	0.952312	0.956384	0.959461	0.961868	0.963803	0.965391	0.966719	0.967845	0.968812	0.969652	0.970389	0.971039	0.971618	0.972137	0.972604
509	1.048979	1.01581				1.028225				1.135011	1.106287	1.089741	1.078982	1.071426	1.065829	1.061515	1.05809	1.055303	1.052992	1.051045	1.049381	1.047944	1.046689	1.045585	1.044605	1.04373	1.042944
510	0.92447	0.973488				0.97477				0.899151	0.916934	0.927946	0.935435	0.940859	0.944968	0.948189	0.950782	0.952914	0.954697	0.956212	0.957514	0.958646	0.959638	0.960515	0.961296	0.961996	0.962626
511	0.988048	0.995984				1.000659				1.002863	1.002311	1.00198	1.00176	1.001602	1.001484	1.001393	1.001319	1.001259	1.001209	1.001167	1.001131	1.001099	1.001072	1.001047	1.001026	1.001007	1.000989
512	0.744821	0.897504				0.89121				0.654035	0.70065	0.731952	0.754421	0.771333	0.784524	0.7951	0.803768	0.811002	0.81713	0.822388	0.82695	0.830944	0.834471	0.837608	0.840416	0.842945	0.845233
513	1.094598	1.029662				1.030114				1.145055	1.11398	1.096132	1.084547	1.076422	1.070407	1.065775	1.062098	1.059109	1.05663	1.054542	1.052759	1.051218	1.049874	1.048691	1.047641	1.046704	1.045862
514	0.598449	0.817219				0.7976				0.476274	0.529615	0.567767	0.596411	0.618705	0.636552	0.651161	0.66334	0.673648	0.682487	0.690149	0.696855	0.702772	0.708034	0.712742	0.716979	0.720814	0.724301
515	1.140158	1.042727				1.068762				1.386575	1.290628	1.239179	1.2071	1.185185	1.169263	1.157173	1.147679	1.140026	1.133727	1.12845	1.123967	1.12011	1.116756	1.113814	1.111212	1.108894	1.106816
516	0.807595	0.926428				0.941772				0.788693	0.8221	0.843538	0.858462	0.869449	0.877876	0.884544	0.889952	0.894426	0.898189	0.901398	0.904167	0.90658	0.908702	0.910583	0.912262	0.913769	0.915129
517	1.28095	1.078876				1.136529				2.08575	1.725474	1.56344	1.471329	1.411911	1.370405	1.339772	1.316234	1.297582	1.282438	1.269897	1.259342	1.250334	1.242558	1.235776	1.22981	1.22452	1.219799
518	1.070361	1.022403				0.972627				0.891303	0.910332	0.922144	0.930191	0.936025	0.940449	0.943919	0.946713	0.949012	0.950936	0.95257	0.953975	0.955196	0.956268	0.957215	0.958058	0.958814	0.959495
519	0.95812	0.985639				0.974014				0.89637	0.914597	0.925893	0.933581	0.93915	0.943371	0.94668	0.949344	0.951535	0.953369	0.954926	0.956264	0.957428	0.958448	0.95935	0.960153	0.960872	0.961521
520	0.717242	0.883853				0.877546				0.623177	0.671864	0.704908	0.728804	0.746889	0.761053	0.772447	0.78181	0.789641	0.796289	0.802001	0.806963	0.811313	0.815159	0.818582	0.821649	0.824413	0.826916
521	0.995483	0.99849				0.987207				0.94683	0.956611	0.962578	0.966597	0.969488	0.971668	0.973371	0.974737	0.975857	0.976793	0.977586	0.978267	0.978858	0.979376	0.979833	0.98024	0.980604	0.980932
522	1.300258	1.083393				1.109439				1.746592	1.527308	1.420316	1.356945	1.315034	1.285262	1.263022	1.245776	1.232013	1.220773	1.211422	1.20352	1.196754	1.190896	1.185775	1.181259	1.177248	1.173662
523	0.818092	0.930996				0.907289				0.693096	0.736568	0.765372	0.785859	0.801177	0.813064	0.822555	0.83031	0.836764	0.842219	0.846891	0.850938	0.854476	0.857596	0.860368	0.862847	0.865077	0.867094
524	1.340888	1.092588				1.088094				1.540437	1.395411	1.320803	1.275343	1.244742	1.222738	1.206155	1.193208	1.18282	1.174301	1.167188	1.161159	1.155984	1.151493	1.14756	1.144086	1.140996	1.138229
525	0.651228	0.848522				0.874714				0.61703	0.666086	0.699451	0.723616	0.741924	0.756275	0.767827	0.777326	0.785274	0.792023	0.797824	0.802865	0.807286	0.811194	0.814674	0.817793	0.820603	0.823149
526	1.102103	1.031865				0.999253				0.996769	0.997389	0.997761	0.998009	0.998187	0.99832	0.998423	0.998506	0.998574	0.99863	0.998678	0.998719	0.998755	0.998786	0.998813	0.998838	0.99886	0.998879
527	0.929629	0.975388				0.995552				0.981008	0.984604	0.986774	0.988227	0.989267	0.990048	0.990657	0.991144	0.991543	0.991876	0.992158	0.992399	0.992609	0.992792	0.992954	0.993098	0.993227	0.993343
528	1.274121	1.077255				1.068144				1.382076	1.287477	1.236689	1.204999	1.183339	1.167599	1.155643	1.146254	1.138684	1.132451	1.127231	1.122795	1.118978	1.115659	1.112748	1.110172	1.107878	1.105821
529	1.136246	1.041634				1.045105				1.230042	1.177932	1.148732	1.130057	1.117085	1.10755	1.100246	1.094471	1.089792	1.085922	1.08267	1.079897	1.077506	1.075422	1.07359	1.071967	1.070519	1.069219
530	0.721696	0.886099				0.871639				0.610446	0.659881	0.693581	0.718027	0.736571	0.75112	0.76284	0.772482	0.780554	0.787411	0.793308	0.798433	0.802929	0.806904	0.810445	0.813618	0.816479	0.81907
531	0.667349	0.857519				0.878896				0.626137	0.674641	0.707527	0.731292	0.749268	0.763341	0.774658	0.783956	0.791731	0.798329	0.803998	0.808922	0.813238	0.817053	0.820449	0.823491	0.826233	0.828716
532	0.961671	0.986889				1.00406				1.017833	1.014355	1.012279	1.0109	1.009917	1.009181	1.008609	1.008152	1.007779	1.007468	1.007205	1.00698	1.006784	1.006614	1.006463	1.006329	1.00621	1.006102
533	1.071386	1.022714				1.049925				1.25953	1.199656	1.166388	1.145216	1.130557	1.119807	1.111586	1.105096	1.099842	1.095502	1.091856	1.08875	1.086073	1.083741	1.081691	1.079876	1.078257	1.076804
534	1.219404	1.063802				1.078531				1.460966	1.342001	1.279489	1.240952	1.214817	1.195927	1.181636	1.170447	1.161448	1.154055	1.147871	1.142624	1.138115	1.134199	1.130766	1.127731	1.12503	1.12261
535	1.142907	1.043492				1.066487				1.370143	1.279096	1.230052	1.199394	1.178415	1.163156	1.151558	1.142445	1.135096	1.129043	1.123971	1.119661	1.115951	1.112726	1.109895	1.107391	1.10516	1.10316
536	1.165035	1.049559				1.070917				1.402437	1.301697	1.247912	1.214459	1.191641	1.175083	1.162519	1.152659	1.144716	1.13818	1.132707	1.128058	1.12406	1.120584	1.117535	1.114839	1.112438	1.110286
537	1.26339	1.074683				1.087431				1.534697	1.391604	1.317877	1.272918	1.242638	1.220857	1.204436	1.191615	1.181326	1.172886	1.165839	1.159865	1.154737	1.150288	1.14639	1.142947	1.139884	1.137141
538	1.105088	1.032736				1.013524				1.061372	1.048992	1.041701	1.036897	1.033492	1.030953	1.028987	1.027419	1.026141	1.025077	1.024179	1.023411	1.022746	1.022164	1.021652	1.021197	1.02079	1.020425
539	0.958324	0.985711				1.000542				1.002353	1.0019	1.001628	1.001447	1.001318	1.001221	1.001145	1.001085	1.001035	1.000994	1.000959	1.00093	1.000904	1.000881	1.000861	1.000844	1.000828	1.000813
540	1.092577	1.029065				1.024542				1.115825	1.091512	1.077427	1.068237	1.061768	1.056967	1.053264	1.050319	1.047922	1.045933	1.044256	1.042823	1.041584	1.040502	1.03955	1.038704	1.037949	1.037271

	A	B	C	D	E	F	G	H	I	J	K	L	M	N	O	P	Q	R	S	T	U	V	W	X	Y	Z	AA
541	0.655368	0.850856				0.887008				0.644328	0.691635	0.723507	0.746438	0.763729	0.777232	0.788068	0.796958	0.804382	0.810675	0.816077	0.820765	0.824872	0.828499	0.831726	0.834616	0.837219	0.839575
542	0.869241	0.952251				0.972394				0.890454	0.909617	0.921515	0.929622	0.935501	0.939958	0.943455	0.946271	0.948588	0.950527	0.952174	0.953591	0.954821	0.955901	0.956856	0.957706	0.958468	0.959155
543	1.3917	1.103531				1.156406				2.415995	1.898909	1.68281	1.564142	1.489135	1.437436	1.399643	1.370809	1.348087	1.32972	1.314565	1.301847	1.291022	1.281697	1.27358	1.266451	1.26014	1.254513
544	1.429111	1.11122				1.122645				1.89898	1.619073	1.487518	1.411082	1.361124	1.325916	1.299767	1.279579	1.263522	1.250446	1.239591	1.230436	1.22261	1.215843	1.209935	1.204731	1.200112	1.195986
545	1.130351	1.039976				1.066234				1.368336	1.277823	1.229043	1.198541	1.177665	1.162479	1.150936	1.141865	1.134549	1.128523	1.123474	1.119183	1.11549	1.112278	1.10946	1.106967	1.104746	1.102754
546	0.772002	0.910378				0.930021				0.754114	0.791542	0.815838	0.83288	0.845496	0.855212	0.862925	0.869195	0.874394	0.878774	0.882515	0.885747	0.888567	0.891049	0.893251	0.895217	0.896984	0.89858
547	1.278534	1.078304				1.08671				1.528494	1.387479	1.314705	1.270287	1.240353	1.218813	1.20257	1.189884	1.179702	1.171349	1.164373	1.158459	1.153383	1.148977	1.145117	1.141708	1.138675	1.135959
548	0.927944	0.974769				0.9802				0.919512	0.933968	0.942862	0.948887	0.953237	0.956526	0.9591	0.961169	0.962868	0.964289	0.965495	0.96653	0.96743	0.968218	0.968915	0.969535	0.970091	0.970591
549	0.956439	0.985045				0.935507				0.76998	0.805616	0.828627	0.844711	0.856588	0.865717	0.872953	0.878829	0.883696	0.887794	0.891291	0.89431	0.896943	0.89926	0.901314	0.903148	0.904795	0.906283
550	1.032832	1.010709				1.000738				1.003206	1.002588	1.002217	1.00197	1.001794	1.001662	1.001559	1.001477	1.00141	1.001354	1.001306	1.001266	1.001231	1.0012	1.001173	1.001148	1.001127	1.001107
551	0.952639	0.983698				0.948964				0.810997	0.841586	0.861072	0.874572	0.884478	0.892055	0.898039	0.902884	0.906887	0.910251	0.913116	0.915587	0.917739	0.91963	0.921305	0.922799	0.92414	0.925351
552	0.914627	0.969825				0.995624				0.981308	0.984848	0.986985	0.988414	0.989438	0.990207	0.990806	0.991285	0.991678	0.992006	0.992283	0.992521	0.992727	0.992908	0.993067	0.993209	0.993336	0.99345
553	0.967439	0.988905				0.980014				0.918805	0.933379	0.942348	0.948423	0.952811	0.956129	0.958725	0.960812	0.962527	0.96396	0.965176	0.966221	0.967129	0.967924	0.968627	0.969253	0.969813	0.970318
554	0.812427	0.92854				0.904491				0.686071	0.730151	0.759427	0.780284	0.795897	0.808024	0.817714	0.825635	0.832231	0.837809	0.842587	0.846727	0.850347	0.85354	0.856378	0.858916	0.8612	0.863266
555	1.255826	1.072851				1.091249				1.568251	1.413757	1.334856	1.286973	1.254821	1.231742	1.21437	1.200822	1.18996	1.181057	1.173627	1.167332	1.161932	1.157247	1.153144	1.149522	1.1463	1.143416
556	1.143278	1.043595				1.07635				1.443796	1.330264	1.270329	1.233285	1.208121	1.189912	1.176124	1.165322	1.156631	1.149486	1.143509	1.138435	1.134074	1.130286	1.126964	1.124027	1.121413	1.11907
557	0.870394	0.952712				0.969489				0.879993	0.900781	0.913732	0.922576	0.928998	0.933873	0.937701	0.940786	0.943325	0.945451	0.947258	0.948812	0.950163	0.951348	0.952396	0.95333	0.954167	0.954922
558	0.960722	0.986555				1.026383				1.125352	1.098862	1.083559	1.073592	1.066583	1.061387	1.05738	1.054197	1.051606	1.049457	1.047645	1.046097	1.044759	1.043592	1.042564	1.041651	1.040836	1.040104
559	0.999923	0.999974				0.991061				0.962387	0.969399	0.973655	0.976514	0.978566	0.980111	0.981315	0.982281	0.983073	0.983734	0.984294	0.984774	0.985191	0.985556	0.985878	0.986164	0.986421	0.986652
560	1.201891	1.059314				1.073123				1.418996	1.313184	1.256948	1.222058	1.1983	1.181079	1.168023	1.157784	1.14954	1.142759	1.137083	1.132263	1.128118	1.124516	1.121357	1.118564	1.116077	1.113847
561	1.087203	1.027471				0.992755				0.969347	0.975095	0.978576	0.980911	0.982586	0.983846	0.984828	0.985615	0.98626	0.986798	0.987254	0.987645	0.987984	0.98828	0.988543	0.988776	0.988984	0.989172
562	1.117193	1.036233				1.007976				1.035506	1.028483	1.024315	1.021555	1.019593	1.018126	1.016988	1.01608	1.015338	1.01472	1.014198	1.013751	1.013364	1.013026	1.012727	1.012462	1.012225	1.012012
563	0.758039	0.903834				0.851734				0.57002	0.621403	0.656933	0.682967	0.702862	0.718562	0.731266	0.741757	0.750568	0.758071	0.764538	0.77017	0.775119	0.779501	0.783409	0.786916	0.790081	0.792951
564	0.774833	0.911688				0.891336				0.654329	0.700923	0.732207	0.754661	0.771563	0.784744	0.795312	0.803973	0.811201	0.817324	0.822578	0.827136	0.831127	0.83465	0.837785	0.84059	0.843117	0.845403
565	0.910055	0.968106				0.945308				0.799546	0.831603	0.852102	0.866339	0.876802	0.884818	0.891154	0.896288	0.900534	0.904102	0.907144	0.909767	0.912053	0.914063	0.915844	0.917432	0.918859	0.920146
566	1.194739	1.057454				1.066318				1.368937	1.278246	1.229379	1.198825	1.177914	1.162704	1.151143	1.142058	1.134731	1.128696	1.12364	1.119342	1.115643	1.112427	1.109605	1.107108	1.104884	1.102889
567	0.882949	0.957681				0.952472				0.822212	0.851319	0.869793	0.882562	0.891914	0.899059	0.904696	0.909257	0.913023	0.916185	0.918878	0.921199	0.92322	0.924995	0.926567	0.92797	0.929228	0.930363
568	1.016938	1.005583				0.979352				0.916287	0.93128	0.940513	0.946771	0.951292	0.954711	0.957388	0.95954	0.961308	0.962786	0.96404	0.965118	0.966054	0.966875	0.9676	0.968245	0.968824	0.969345
569	0.963471	0.98752				0.97465				0.89871	0.916564	0.92762	0.935141	0.940588	0.944715	0.94795	0.950554	0.952695	0.954487	0.956008	0.957316	0.958453	0.959449	0.96033	0.961115	0.961818	0.962451
570	0.901464	0.964845				0.965803				0.866976	0.889737	0.903976	0.913725	0.920818	0.926211	0.930449	0.933867	0.936683	0.939042	0.941048	0.942774	0.944275	0.945592	0.946758	0.947796	0.948727	0.949567
571	0.914287	0.969698				0.991955				0.966048	0.972397	0.976247	0.97883	0.980684	0.982079	0.983166	0.984038	0.984753	0.985349	0.985854	0.986287	0.986663	0.986992	0.987283	0.987541	0.987772	0.987981
572	1.022098	1.007259				1.004878				1.021487	1.017284	1.014778	1.013115	1.01193	1.011043	1.010354	1.009804	1.009354	1.008979	1.008663	1.008391	1.008156	1.007951	1.00777	1.007609	1.007464	1.007335
573	1.278922	1.078396				1.112159				1.776222	1.545517	1.433781	1.367854	1.324356	1.293507	1.270488	1.252655	1.238433	1.226825	1.217172	1.209017	1.202038	1.195997	1.190717	1.186063	1.181929	1.178233
574	0.763693	0.906502				0.889836				0.650839	0.697686	0.729177	0.7518	0.768838	0.782132	0.792794	0.801535	0.808832	0.815014	0.82032	0.824923	0.828955	0.832515	0.835681	0.838517	0.841069	0.843381
575	0.896232	0.96284				0.956088				0.834012	0.861512	0.8789	0.890887	0.899652	0.90634	0.91161	0.915871	0.919387	0.922337	0.924848	0.927012	0.928895	0.930549	0.932014	0.933319	0.934491	0.935547
576	1.12688	1.038995				1.023849				1.112271	1.088764	1.075131	1.06623	1.059962	1.055309	1.051719	1.048864	1.046539	1.04461	1.042983	1.041593	1.040391	1.039341	1.038417	1.037597	1.036864	1.036205
577	1.244063	1.06997				1.0815				1.484896	1.358241	1.292114	1.251494	1.224009	1.204175	1.189187	1.177463	1.168041	1.160304	1.153836	1.14835	1.143637	1.139545	1.135959	1.13279	1.12997	1.127443
578	0.866503	0.951154				0.954742				0.82959	0.857697	0.875495	0.887777	0.896762	0.903622	0.90903	0.913403	0.917012	0.920042	0.922622	0.924844	0.926779	0.928479	0.929983	0.931325	0.932529	0.933615
579	1.118345	1.036564				1.075539				1.437499	1.325942	1.266949	1.230452	1.205645	1.187686	1.174083	1.163424	1.154845	1.147792	1.141892	1.136882	1.132576	1.128834	1.125553	1.122653	1.120071	1.117757
580	1.274279	1.077293				1.013185				1.059759	1.047719	1.040625	1.035949	1.032634	1.030162	1.028248	1.026721	1.025476	1.02444	1.023565	1.022817	1.022169	1.021603	1.021104	1.020661	1.020264	1.019908
581	1.348137	1.094186				1.101995				1.669647	1.479163	1.384398	1.327692	1.28995	1.263022	1.242844	1.227159	1.214618	1.204361	1.195816	1.188588	1.182394	1.177027	1.172331	1.168189	1.164508	1.161214
582	0.993011	0.997659				0.971777				0.888217	0.90773	0.919855	0.92812	0.934115	0.938662	0.94223	0.945104	0.947468	0.949447	0.951128	0.952574	0.953831	0.954933	0.955907	0.956775	0.957553	0.958255
583	0.854702	0.946373				0.940314				0.78428	0.818223	0.840038	0.855238	0.866437	0.875031	0.881833	0.887352	0.891919	0.895761	0.899037	0.901865	0.90433	0.906499	0.90842	0.910135	0.911675	0.913066
584	0.866626	0.951203				0.964263				0.861623	0.885179	0.89994	0.910058	0.917426	0.92303	0.927436	0.930992	0.933921	0.936377	0.938464	0.940261	0.941824	0.943196	0.94441	0.945491	0.946461	0.947335
585	0.860259	0.948634				0.980288				0.919849	0.934249	0.943108	0.949108	0.95344	0.956715	0.959278	0.961339	0.963031	0.964446	0.965646	0.966678	0.967573	0.968358	0.969052	0.96967	0.970223	0.970721
586	1.105681	1.032908				1.034642				1.169715	1.132745	1.111664	1.09804	1.088512	1.081474	1.076062	1.071771	1.068286	1.065399	1.062968	1.060894	1.059102	1.05754	1.056165	1.054946	1.053857	1.05288
587	0.987947	0.99595				0.979724				0.917698	0.932456	0.941541	0.947697	0.952143	0.955506	0.958137	0.960253	0.961991	0.963444	0.964677	0.965737	0.966657	0.967463	0.968176	0.96881	0.969378	0.969891
588	0.836712	0.938922				0.954468				0.828694	0.856924	0.874805	0.887145	0.896175	0.90307	0.908506	0.912902	0.91653	0.919576	0.922169	0.924404	0.926349	0.928058	0.929571	0.93092	0.93213	0.933222
589	0.900921	0.964638				0.955613				0.832448	0.860163	0.877697	0.889788	0.898631	0.90538	0.910699	0.914999	0.918548	0.921527	0.924062	0.926247	0.928148	0.929818	0.931297	0.932616	0.933798	0.934865
590	1.38609	1.102352				1.121793				1.888471	1.612892	1.483043	1.4075	1.358088	1.323247	1.29736	1.277368	1.261464	1.24851	1.237755	1.228683	1.220927	1.214221	1.208364	1.203206	1.198628	1.194537
591	0.892413	0.961367				0.976921				0.907135	0.92363	0.933818	0.940736	0.94574	0.949529	0.952496	0.954883	0.956846	0.958487	0.95988	0.961078	0.962118	0.96303	0.963836	0.964554	0.965197	0.965777
592	1.010068	1.003334				0.971433				0.886974	0.906681	0.918932	0.927284	0.933344	0.937941	0.941548	0.944453	0.946844	0.948846	0.950546	0.952008	0.953279	0.954393	0.955379	0.956257	0.957044	0.957753
593	1.00664	1.002204				0.957865				0.8399	0.866581	0.883419	0.895012	0.903481	0.909939	0.915026	0.919137	0.922528	0.925372	0.927793	0.929879	0.931693	0.933287	0.934698	0.935955	0.937084	0.938101
594	1.005767	1.001915				0.985337				0.939423	0.950495	0.957265	0.961832	0.965121	0.967603	0.969542	0.971099	0.972376	0.973443	0.974348	0.975125	0.975799	0.97639	0.976911	0.977376	0.977792	0.978166
595	1.120147	1.037079				1.065649				1.364172	1.274888	1.226715	1.196573	1.175934	1.160916	1.149498	1.140524	1.133285	1.127322	1.122326	1.118079	1.114423	1.111244	1.108455	1.105987	1.103788	1.101816
596	0.759281	0.904422				0.869507				0.605937	0.655621	0.689545	0.71418	0.732883	0.747566	0.759399	0.769139	0.777296	0.784226	0.790188	0.795371	0.799917	0.803939	0.807521	0.810732	0.813626	0.816249
597	0.855586	0.946734				0.985442				0.939836	0.950837	0.957562	0.962099	0.965366	0.967831	0.969756	0.971302	0.972571	0.973631	0.974529	0.975301	0.97597	0.976557	0.977075	0.977536	0.977949	0.978321
598	0.909039	0.967722				0.99824				0.992418	0.993867	0.994738	0.99532	0.995736	0.996049	0.996292	0.996486	0.996645	0.996778	0.99689	0.996987	0.99707	0.997143	0.997208	0.997265	0.997316	0.997362
599	1.155752	1.047034				1.077273				1.451022	1.335212	1.274194	1.236522	1.21095	1.192453	1.178454	1.167488	1.158667	1.151417	1.145354	1.140207	1.135783	1.131941	1.128572	1.125594	1.122943	1.120568
600	1.0057	1.001893				0.992284				0.967401	0.973504	0.977203	0.979685	0.981465	0.982804	0.983849	0.984686	0.985372	0.985944	0.986429	0.986845	0.987205	0.987521	0.9878	0.988048	0.98827	0.98847
601	0.782651	0.915273				0.923154				0.734905	0.774383	0.800173	0.818343	0.831835	0.842249	0.850532	0.857276	0.862873	0.867594	0.87163	0.875119	0.878165	0.880848	0.883229	0.885356	0.887268	0.888996
602	0.555983	0.789762				0.77247				0.439294	0.492387	0.530885	0.560078	0.582977	0.601419	0.616589	0.629288	0.640073	0.649348	0.657408	0.664478	0.670729	0.676296	0.681286	0.685783	0.689857	0.693566
603	0.690232	0.869871				0.840196				0.548188	0.60035	0.636701	0.663484	0.684037	0.700307	0.713506	0.72443	0.733619	0.741456	0.74822	0.754117	0.759303	0.7639	0.768002	0.771686	0.775012	0.77803
604	1.119274	1.03683				1.047936				1.247226	1.19062	1.159057	1.138929	1.124974	1.114731	1.106892	1.100699	1.095684	1.091539	1.088057	1.08509	1.082531	1.080302	1.078343	1.076607	1.075059	1.07367
605	0.809486	0.927256				0.923393				0.735564	0.774973	0.800714	0.818845	0.832308	0.842698	0.850961	0.857689	0.863273	0.867983	0.872008	0.875488	0.878526	0.881202	0.883577	0.885699	0.887606	0.889329
606	1.164058	1.049295				1.035774				1.176006	1.137505	1.115591	1.101444	1.091558	1.084258	1.078648	1.074201	1.070591	1.0676	1.065083	1.062934	1.061079	1.059461	1.058038	1.056776	1.055649	1.054638
607	1.040827	1.013248				1.047803				1.246413	1.190021	1.158571	1.138512	1.124603	1.114393	1.106579	1.100407	1.095407	1.091276	1.087804	1.084846	1.082295	1.080073	1.07812	1.07639	1.074846	1.073461
608	0.745581	0.897872				0.910786				0.70202	0.744694	0.772883	0.792892	0.80783	0.819408	0.828646	0.836187	0.84246	0.84776	0.852297	0.856224	0.859657	0.862684	0.865373	0.867776	0.869939	0.871894
609	0.683363	0.866213				0.841944				0.551424	0.603483	0.639721	0.666397	0.686856	0.703044	0.716171	0.727032	0.736166	0.743955	0.750676	0.756533	0.761685	0.76625	0.770324	0.773981	0.777284	0.78028
610	0.76356	0.906439				0.897978				0.670095	0.715488	0.745801	0.767478	0.783749	0.796413	0.806549	0.814845	0.821761	0.827615	0.832634	0.836984	0.840791	0.844151	0.847138	0.849811	0.852217	0.854394
611	0.805938	0.9257				0.901624				0.678975	0.72365	0.753393	0.774618	0.790526	0.802892	0.812782	0.82087	0.827609	0.833309	0.838195	0.842428	0.846131	0.849399	0.852303	0.854901	0.857239	0.859354
612	1.263463	1.074701				1.022296				1.104375	1.082644	1.070011	1.061752	1.05593	1.051605	1.048266	1.04561	1.043446	1.041651	1.040136	1.038841	1.037721	1.036744	1.035883	1.035118	1.034435	1.033822
613	0.670623	0.859315				0.826518				0.523685	0.576491	0.613615	0.64114	0.662363	0.679226	0.692947	0.704329	0.713924	0.722122	0.729207	0.735391	0.740837	0.745668	0.749983	0.753861	0.757365	0.760547
614	1.194882	1.057491				1.082922				1.496588	1.366126	1.298224	1.256586	1.228443	1.208149	1.192823	1.180839	1.171212	1.163308	1.156703	1.151101	1.14629	1.142113	1.138453	1.135219	1.132341	1.129763
615	0.903963	0.965798				0.986512				0.944067	0.954333	0.960599	0.964823	0.967863	0.970156	0.971946	0.973383	0.974562	0.975547	0.976382	0.977099	0.977721	0.978266	0.978747	0.979175	0.979558	0.979904
616	1.188619	1.05585				1.084598				1.51057	1.375513	1.305481	1.262625	1.233696	1.212855	1.197126	1.184833	1.174962	1.16686	1.160092	1.154353	1.149424	1.145147	1.141398	1.138087	1.135141	1.132502
617	0.690431	0.869976				0.877316				0.622674	0.671392	0.704463	0.728381	0.746484	0.760664	0.77207	0.781445	0.789286	0.795941	0.801661	0.806629	0.810986	0.814836	0.818264	0.821335	0.824103	0.82661
618	1.317575	1.087362				1.07112				1.403948	1.302748	1.24874	1.215156	1.192252	1.175633	1.163024	1.15313	1.145159	1.138601	1.13311	1.128445	1.124433	1.120946	1.117887	1.115182	1.112773	1.110613
619	0.75586	0.9028				0.908738				0.696775	0.739922	0.768473	0.788765	0.803927	0.815687	0.825074	0.832741	0.83912	0.844512	0.849128	0.853125	0.85662	0.859702	0.862439	0.864887	0.86709	0.869081
620	0.879378	0.956277				0.971246				0.886296	0.906109	0.918428	0.926828	0.932923	0.937547	0.941176	0.944099	0.946504	0.948517	0.950228	0.951699	0.952977	0.954099	0.955091	0.955974	0.956766	0.95748
621	1.17001	1.050901				0.983459				0.932066	0.944404	0.951965	0.957073	0.960755	0.963536	0.965709	0.967455	0.968889	0.970086	0.971102	0.971974	0.972732	0.973395	0.973981	0.974503	0.97497	0.975391
622	0.894994	0.962363				0.959718				0.846107	0.871911	0.888163	0.899339	0.907495	0.91371	0.918603	0.922555	0.925814	0.928548	0.930873	0.932876	0.934619	0.936149	0.937503	0.93871	0.939793	0.94077
623	0.848199	0.943702				0.98419				0.934921	0.94677	0.954024	0.958923	0.962453	0.965117	0.9672	0.968873	0.970245	0.971392	0.972365	0.9732	0.973925	0.974561	0.975122	0.975621	0.976068	0.976471
624	1.120552	1.037195				1.088894				1.54741	1.400027	1.324345	1.278278	1.247288	1.225013	1.208231	1.195133	1.184626	1.17601	1.168817	1.162721	1.157489	1.15295	1.148974	1.145463	1.142339	1.139543
625	1.26298	1.074584				1.075312				1.435743	1.324734	1.266004	1.22966	1.204952	1.187063	1.173512	1.162892	1.154345	1.147318	1.141439	1.136447	1.132156	1.128428	1.125158	1.122268	1.119695	1.117389
626	0.85137	0.945008				0.963222				0.858032	0.882115	0.897225	0.907589	0.91514	0.920886	0.925405	0.929053	0.932059	0.934578	0.936721	0.938565	0.94017	0.941578	0.942824	0.943935	0.944931	0.945829
627	0.553895	0.788354				0.764074				0.427712	0.480605	0.519124	0.548427	0.571468	0.590061	0.60538	0.61822	0.629138	0.638535	0.646708	0.653882	0.66023	0.665886	0.670958	0.675532	0.679677	0.683452
628	1.254334	1.072487				1.087451				1.534871	1.391719	1.317966	1.272992	1.242702	1.220914	1.204489	1.191663	1.181371	1.172929	1.16588	1.159904	1.154775	1.150324	1.146425	1.142982	1.139918	1.137175
629	0.592	0.813187				0.835935				0.540399	0.592793	0.629407	0.656437	0.677211	0.693675	0.707044	0.718117	0.727437	0.735391	0.742259	0.748248	0.753517	0.758189	0.76236	0.766106	0.769489	0.77256
630	1.041323	1.013405				0.978001				0.911183	0.927016	0.936784	0.94341	0.948201	0.951826	0.954665	0.956948	0.958825	0.960394	0.961726	0.96287	0.963864	0.964736	0.965506	0.966192	0.966806	0.96736
631	0.877373	0.955485				0.961871				0.853406	0.878162	0.893718	0.904398	0.912184	0.918112	0.922777	0.926543	0.929647	0.932249	0.934463	0.936369	0.938027	0.939482	0.940771	0.941918	0.942948	0.943876
632	1.07375	1.023431				1.0477				1.245777	1.189553	1.15819	1.138185	1.124313	1.114129	1.106335	1.100178	1.095191	1.091069	1.087606	1.084655	1.082111	1.079894	1.077945	1.076219	1.07468	1.073298
633	1.063593	1.020336				0.986521				0.944104	0.954363	0.960626	0.964847	0.967885	0.970176	0.971965	0.973401	0.97458	0.975564	0.976398	0.977114	0.977736	0.97828	0.978761	0.979189	0.979572	0.979917
634	0.96303	0.987365				1.020401				1.094855	1.075241	1.063806	1.056318	1.051033	1.047103	1.044068	1.041652	1.039683	1.038049	1.03667	1.03549	1.03447	1.03358	1.032795	1.032099	1.031476	1.030917
635	1.413663	1.108082				1.08082				1.479361	1.354497	1.289209	1.249071	1.221897	1.202281	1.187454	1.175853	1.166529	1.15887	1.152469	1.147037	1.142371	1.13832	1.134769	1.131631	1.128838	1.126336
636	1.034318	1.011183				1.027257				1.129915	1.102374	1.086484	1.076143	1.068876	1.06349	1.059339	1.056041	1.053357	1.051132	1.049256	1.047653	1.046268	1.045059	1.043995	1.043051	1.042208	1.04145
637	1.278736	1.078352				1.085492				1.518117	1.380561	1.309376	1.265862	1.23651	1.215374	1.199428	1.186969	1.176967	1.168759	1.161903	1.15609	1.151099	1.146767	1.142972	1.13962	1.136636	1.133965
638	0.904386	0.965959				1.0182				1.083959	1.066736	1.056662	1.050051	1.045379	1.041903	1.039215	1.037074	1.03533	1.03388	1.032657	1.031611	1.030706	1.029915	1.029218	1.0286	1.028047	1.02755
639	1.214211	1.062481				1.072648				1.415402	1.310697	1.254994	1.220416	1.196862	1.179784	1.166835	1.156678	1.148499	1.141771	1.136139	1.131356	1.127243	1.123668	1.120533	1.117761	1.115292	1.11308
640	1.152334	1.046097				1.067351				1.376347	1.283457	1.233508	1.202313	1.180981	1.165471	1.153687	1.14443	1.136966	1.13082	1.125671	1.121295	1.117529	1.114255	1.111382	1.108841	1.106577	1.104548
641	0.936037	0.977729				0.990196				0.958861	0.966507	0.971154	0.974276	0.976519	0.978208	0.979526	0.980582	0.981449	0.982172	0.982784	0.98331	0.983766	0.984166	0.984518	0.984832	0.985113	0.985366
642	1.191326	1.056561				1.056414				1.301075	1.229867	1.190764	1.166049	1.149014	1.136561	1.12706	1.119573	1.113521	1.108527	1.104337	1.10077	1.097697	1.095023	1.092674	1.090594	1.08874	1.087077
643	0.997391	0.999129				1.021477				1.100245	1.079436	1.067324	1.059399	1.05381	1.049657	1.04645	1.043898	1.041819	1.040093	1.038637	1.037392	1.036316	1.035376	1.034548	1.033813	1.033156	1.032566
644	0.750641	0.900308				0.910268				0.700687	0.743482	0.771763	0.791844	0.806839	0.818464	0.82774	0.835313	0.841613	0.846936	0.851493	0.855439	0.858888	0.861928	0.864629	0.867044	0.869217	0.871181
645	0.897162	0.963198				0.896424				0.666361	0.712047	0.742594	0.764458	0.78088	0.793668	0.803907	0.81229	0.819281	0.825198	0.830273	0.834673	0.838523	0.841922	0.844944	0.847648	0.850083	0.852286
646	0.808011	0.92661				0.922628				0.733464	0.77309	0.79899	0.817243	0.830799	0.841266	0.84959	0.85637	0.861997	0.866744	0.870801	0.874309	0.877372	0.88007	0.882465	0.884604	0.886527	0.888265
647	0.811079	0.927952				0.93315				0.763106	0.79953	0.823102	0.839605	0.851804	0.861188	0.868631	0.874678	0.879689	0.883909	0.887512	0.890623	0.893337	0.895726	0.897844	0.899735	0.901434	0.902969
648	1.25114	1.071707				1.068188				1.382394	1.2877	1.236865	1.205148	1.18347	1.167717	1.155752	1.146355	1.138779	1.132542	1.127318	1.122878	1.119058	1.115737	1.112823	1.110246	1.10795	1.105892
649	0.901729	0.964946				0.99121				0.962992	0.969895	0.974084	0.976897	0.978917	0.980436	0.981622	0.982572	0.983351	0.984001	0.984552	0.985025	0.985435	0.985794	0.986111	0.986392	0.986645	0.986872
650	0.676397	0.86246				0.878505				0.625278	0.673836	0.706768	0.730571	0.748579	0.762678	0.774017	0.783334	0.791125	0.797737	0.803419	0.808354	0.812681	0.816504	0.819908	0.822958	0.825706	0.828194
651	1.007511	1.002491				0.991145				0.962729	0.969679	0.973897	0.97673	0.978764	0.980294	0.981488	0.982446	0.98323	0.983885	0.98444	0.984915	0.985328	0.98569	0.986009	0.986293	0.986547	0.986776
652	1.182196	1.054154				1.048947				1.253459	1.195202	1.162777	1.14212	1.127809	1.117309	1.109276	1.102933	1.097797	1.093553	1.089987	1.08695	1.084331	1.082049	1.080045	1.078269	1.076685	1.075263
653	0.892827	0.961527				1.003048				1.013345	1.010751	1.009201	1.00817	1.007435	1.006885	1.006457	1.006115	1.005836	1.005603	1.005406	1.005237	1.005091	1.004963	1.00485	1.00475	1.00466	1.004579
654	0.945044	0.980985				0.979465				0.916714	0.931636	0.940824	0.947051	0.95155	0.954952	0.957615	0.959756	0.961515	0.962985	0.964233	0.965305	0.966237	0.967053	0.967774	0.968416	0.968992	0.96951
655	0.985999	0.995289				0.999294				0.996947	0.997533	0.997885	0.998119	0.998287	0.998413	0.99851	0.998589	0.998653	0.998706	0.998751	0.99879	0.998824	0.998853	0.998879	0.998902	0.998923	0.998941
656	1.165253	1.049618				1.029409				1.14129	1.1111	1.093741	1.082466	1.074554	1.068696	1.064183	1.0606	1.057687	1.055271	1.053236	1.051497	1.049995	1.048685	1.047531	1.046507	1.045593	1.044772
657	0.859366	0.948272				0.913438				0.708894	0.750933	0.778638	0.798272	0.812913	0.824252	0.833292	0.840668	0.846801	0.851981	0.856413	0.860249	0.863602	0.866556	0.869181	0.871527	0.873636	0.875544
658	1.109936	1.034143				1.052661				1.276786	1.21226	1.176582	1.153942	1.138296	1.126837	1.118083	1.111178	1.10559	1.100977	1.097104	1.093805	1.090962	1.088487	1.086312	1.084387	1.082669	1.081128
659	1.090143	1.028344				1.018533				1.085598	1.068017	1.057739	1.050997	1.046233	1.042688	1.039948	1.037766	1.035987	1.03451	1.033263	1.032197	1.031275	1.030469	1.029759	1.029129	1.028566	1.028059
660	1.067068	1.021399				1.036945				1.182581	1.142467	1.119679	1.104985	1.094723	1.087151	1.081334	1.076725	1.072983	1.069884	1.067276	1.065051	1.06313	1.061455	1.059981	1.058675	1.057508	1.056461
661	0.64596	0.845527				0.813469				0.501593	0.554766	0.592448	0.620549	0.64231	0.659659	0.673815	0.685585	0.695525	0.704032	0.711394	0.717828	0.723498	0.728534	0.733037	0.737085	0.740746	0.744072
662	0.748565	0.89931				0.902597				0.681372	0.725848	0.755434	0.776536	0.792345	0.804631	0.814453	0.822485	0.829175	0.834835	0.839684	0.843885	0.847561	0.850803	0.853685	0.856263	0.858582	0.860681
663	0.751929	0.900924				0.91888				0.723299	0.76395	0.790611	0.809443	0.823453	0.834283	0.842906	0.849933	0.85577	0.860696	0.864909	0.868553	0.871736	0.87454	0.87703	0.879254	0.881255	0.883063
664	0.807662	0.926457				0.911645				0.704235	0.746706	0.77474	0.794629	0.809472	0.820973	0.830147	0.837635	0.843863	0.849124	0.853628	0.857526	0.860933	0.863937	0.866604	0.868989	0.871135	0.873075
665	0.906749	0.966856				0.940373				0.784456	0.818378	0.840177	0.855367	0.866558	0.875144	0.881942	0.887456	0.892019	0.895858	0.899132	0.901957	0.90442	0.906587	0.908507	0.91022	0.911759	0.913148
666	1.107169	1.033341				1.014049				1.063869	1.050961	1.043365	1.038362	1.034817	1.032175	1.030129	1.028498	1.027168	1.026061	1.025127	1.024328	1.023636	1.023032	1.022499	1.022026	1.021603	1.021222
667	0.858109	0.947762				0.972678				0.891488	0.910488	0.922281	0.930315	0.93614	0.940556	0.94402	0.94681	0.949104	0.951025	0.952656	0.954059	0.955278	0.956347	0.957293	0.958135	0.958889	0.95957
668	1.131735	1.040366				1.049488				1.256812	1.197663	1.164773	1.143832	1.129329	1.11869	1.110554	1.104129	1.098928	1.094631	1.091021	1.087946	1.085294	1.082985	1.080955	1.079158	1.077555	1.076116
669	1.102949	1.032112				1.046721				1.239805	1.18515	1.15461	1.13511	1.12158	1.111643	1.104034	1.098022	1.093151	1.089125	1.085742	1.082858	1.080372	1.078205	1.076301	1.074614	1.073109	1.071758
670	1.343382	1.093139				1.105008				1.700082	1.498357	1.398773	1.339425	1.300027	1.271966	1.250965	1.234657	1.221627	1.210977	1.202109	1.194611	1.188188	1.182624	1.177757	1.173465	1.169652	1.16624
671	1.005807	1.001928				1.003017				1.013207	1.01064	1.009106	1.008086	1.007359	1.006814	1.006391	1.006052	1.005776	1.005545	1.00535	1.005183	1.005039	1.004912	1.0048	1.004701	1.004612	1.004532
672	1.045085	1.01459				1.004933				1.021735	1.017482	1.014947	1.013264	1.012066	1.011169	1.010472	1.009915	1.009461	1.009082	1.008761	1.008487	1.008249	1.008041	1.007858	1.007695	1.007549	1.007418
673	1.114791	1.035544				0.982926				0.929997	0.942688	0.95047	0.95573	0.959522	0.962387	0.964626	0.966426	0.967903	0.969137	0.970184	0.971083	0.971864	0.972548	0.973152	0.97369	0.974172	0.974606
674	0.92812	0.974834				0.952771				0.823177	0.852154	0.870541	0.883246	0.89255	0.899658	0.905265	0.909802	0.913547	0.916692	0.91937	0.921678	0.923687	0.925453	0.927016	0.928411	0.929662	0.930791
675	1.015609	1.005149				1.010155				1.045548	1.036469	1.031097	1.027547	1.025026	1.023143	1.021684	1.020519	1.019568	1.018777	1.018108	1.017536	1.017041	1.016608	1.016226	1.015887	1.015583	1.015311
676	1.193342	1.057089				1.053141				1.279852	1.214491	1.178383	1.155481	1.13966	1.128076	1.119227	1.112248	1.106602	1.10194	1.098027	1.094694	1.091822	1.089321	1.087125	1.085179	1.083444	1.081888
677	0.879315	0.956252				0.931369				0.757969	0.79497	0.818958	0.83577	0.848207	0.857781	0.865378	0.871554	0.876672	0.880984	0.884665	0.887845	0.89062	0.893062	0.895228	0.897162	0.8989	0.900469
678	0.607722	0.822935				0.824181				0.51964	0.572529	0.609765	0.637403	0.658729	0.675684	0.689487	0.700943	0.710602	0.718858	0.725994	0.732225	0.737712	0.742582	0.746932	0.750842	0.754375	0.757583
679	1.229219	1.066278				1.067232				1.375488	1.282854	1.23303	1.20191	1.180626	1.165151	1.153393	1.144156	1.136708	1.130574	1.125436	1.121069	1.117311	1.114044	1.111177	1.108641	1.106382	1.104356
680	0.764896	0.907066				0.893691				0.659861	0.706044	0.736993	0.759179	0.775861	0.788862	0.799279	0.807813	0.814932	0.820962	0.826133	0.830618	0.834545	0.838011	0.841094	0.843853	0.846337	0.848585
681	1.201062	1.059099				1.045908				1.234878	1.18151	1.151648	1.132565	1.119316	1.109582	1.102126	1.096234	1.09146	1.087513	1.084195	1.081368	1.078929	1.076804	1.074936	1.073282	1.071805	1.07048
682	0.832815	0.937281				0.917771				0.720331	0.761275	0.788153	0.807153	0.821294	0.83223	0.840939	0.848038	0.853937	0.858915	0.863173	0.866857	0.870074	0.87291	0.875427	0.877677	0.879699	0.881528
683	0.922478	0.972751				0.992579				0.968617	0.974498	0.978061	0.980451	0.982166	0.983455	0.984461	0.985267	0.985927	0.986478	0.986944	0.987345	0.987692	0.987996	0.988264	0.988503	0.988717	0.988909
684	1.078098	1.024744				1.031217				1.150985	1.118509	1.099888	1.087814	1.079352	1.073091	1.068271	1.064446	1.061337	1.05876	1.056589	1.054735	1.053134	1.051737	1.050508	1.049417	1.048443	1.047568
685	0.831568	0.936754				0.942432				0.790701	0.823862	0.845127	0.859925	0.870816	0.879167	0.885774	0.891131	0.895563	0.89929	0.902468	0.90521	0.907599	0.909701	0.911563	0.913225	0.914717	0.916064
686	0.950321	0.982873				0.963882				0.860306	0.884055	0.898945	0.909153	0.916588	0.922244	0.926692	0.930281	0.933239	0.935718	0.937826	0.93964	0.941218	0.942603	0.943829	0.944921	0.9459	0.946784
687	0.670228	0.859099				0.853621				0.573698	0.62493	0.66031	0.68621	0.70599	0.72159	0.734208	0.744624	0.753369	0.760815	0.767232	0.772818	0.777726	0.782072	0.785947	0.789424	0.792561	0.795406
688	1.215668	1.062853				1.068432				1.384174	1.288947	1.237851	1.205979	1.184201	1.168376	1.156357	1.146919	1.13931	1.133047	1.1278	1.123342	1.119506	1.116171	1.113245	1.110657	1.108352	1.106285
689	0.88486	0.958429				0.941916				0.789128	0.822482	0.843883	0.858779	0.869746	0.878156	0.884811	0.890208	0.894673	0.898428	0.90163	0.904393	0.906801	0.908919	0.910796	0.912471	0.913975	0.915332
690	0.922491	0.972756				0.959626				0.845798	0.871646	0.887927	0.899124	0.907295	0.913522	0.918425	0.922385	0.925651	0.92839	0.93072	0.932727	0.934473	0.936007	0.937364	0.938574	0.939659	0.940637
691	0.929577	0.975369				1.004547				1.020008	1.016098	1.013767	1.012219	1.011116	1.01029	1.009649	1.009136	1.008717	1.008368	1.008073	1.007821	1.007602	1.00741	1.007242	1.007091	1.006957	1.006836
692	0.974306	0.991286				0.964604				0.862805	0.886185	0.900832	0.910869	0.918176	0.923733	0.928103	0.931628	0.934532	0.936967	0.939036	0.940817	0.942367	0.943726	0.944929	0.946001	0.946963	0.947829
693	1.144723	1.043996				1.044187				1.22455	1.17386	1.145411	1.127199	1.114541	1.105233	1.0981	1.092459	1.087887	1.084106	1.080927	1.078218	1.07588	1.073843	1.072052	1.070465	1.069049	1.067778
694	1.147175	1.044675				1.073392				1.42103	1.314591	1.258052	1.222986	1.199112	1.18181	1.168694	1.158409	1.150128	1.143316	1.137616	1.132774	1.128612	1.124995	1.121822	1.119017	1.116519	1.114281
695	1.005792	1.001923				0.951292				0.818415	0.848028	0.866847	0.879865	0.889405	0.896697	0.902452	0.907109	0.910955	0.914185	0.916937	0.919308	0.921373	0.923188	0.924795	0.926228	0.927515	0.928675
696	0.882958	0.957684				0.957868				0.839909	0.866589	0.883426	0.895019	0.903487	0.909945	0.915031	0.919142	0.922532	0.925377	0.927798	0.929883	0.931698	0.933291	0.934702	0.93596	0.937088	0.938105
697	1.143505	1.043658				1.017364				1.079866	1.063531	1.053966	1.047683	1.043242	1.039935	1.037378	1.035341	1.033681	1.032301	1.031137	1.03014	1.029279	1.028526	1.027862	1.027273	1.026747	1.026274
698	1.208307	1.060969				1.030366				1.146403	1.11501	1.096987	1.085291	1.077089	1.071018	1.066343	1.062633	1.059616	1.057115	1.055009	1.053209	1.051655	1.050299	1.049105	1.048046	1.0471	1.046251
699	1.022872	1.007509				0.995903				0.982486	0.985807	0.98781	0.989149	0.990109	0.990829	0.99139	0.99184	0.992208	0.992515	0.992775	0.992997	0.993191	0.99336	0.993509	0.993642	0.99376	0.993867
700	1.03396	1.011069				1.027068				1.128925	1.101613	1.08585	1.07559	1.06838	1.063035	1.058915	1.055641	1.052978	1.050769	1.048907	1.047316	1.045942	1.044742	1.043685	1.042748	1.041911	1.041158
701	0.888964	0.960029				0.970991				0.885378	0.905334	0.917745	0.92621	0.932353	0.937014	0.940671	0.943618	0.946042	0.948072	0.949797	0.95128	0.952569	0.9537	0.9547	0.955591	0.956389	0.957109
702	1.025658	1.008409				1.053464				1.28192	1.215994	1.179596	1.156518	1.140578	1.128909	1.119997	1.112968	1.107282	1.102588	1.098647	1.095292	1.0924	1.089883	1.087671	1.085712	1.083966	1.082399
703	1.306941	1.084934				1.095172				1.604036	1.437101	1.352638	1.301638	1.267501	1.243051	1.224677	1.210365	1.198901	1.189512	1.181682	1.175052	1.169366	1.164435	1.160119	1.15631	1.152922	1.149891
704	1.108305	1.03367				1.052443				1.275394	1.211246	1.175764	1.153242	1.137676	1.126274	1.117563	1.110691	1.10513	1.100539	1.096684	1.093401	1.090571	1.088108	1.085943	1.084026	1.082316	1.080783
705	1.056737	1.018223				1.012828				1.058073	1.046387	1.039499	1.034957	1.031737	1.029335	1.027474	1.02599	1.02478	1.023773	1.022923	1.022195	1.021565	1.021015	1.02053	1.020099	1.019713	1.019367
706	0.830288	0.936212				0.905097				0.687583	0.731534	0.760709	0.781487	0.797037	0.809112	0.81876	0.826645	0.833211	0.838762	0.843518	0.847637	0.85124	0.854417	0.857241	0.859766	0.862039	0.864094
707	1.030262	1.009888				1.024035				1.113224	1.089501	1.075747	1.066769	1.060447	1.055754	1.052133	1.049254	1.04691	1.044965	1.043325	1.041923	1.040711	1.039653	1.038721	1.037894	1.037155	1.036491
708	1.346514	1.093829				1.067462				1.377145	1.284018	1.233951	1.202689	1.18131	1.165768	1.15396	1.144685	1.137206	1.131047	1.125889	1.121504	1.117731	1.114451	1.111573	1.109027	1.106759	1.104725
709	1.013016	1.004301				1.00365				1.016013	1.012894	1.011031	1.009794	1.008911	1.008251	1.007738	1.007327	1.006992	1.006713	1.006476	1.006274	1.006099	1.005945	1.00581	1.00569	1.005582	1.005485
710	1.041509	1.013464				1.044748				1.227904	1.176347	1.147441	1.128946	1.116096	1.106649	1.099412	1.093689	1.089051	1.085216	1.081992	1.079244	1.076874	1.074808	1.072992	1.071383	1.069948	1.068659
711	0.860018	0.948537				0.943753				0.794746	0.827405	0.848322	0.862864	0.87356	0.881758	0.888241	0.893497	0.897843	0.901498	0.904613	0.907301	0.909643	0.911703	0.913528	0.915156	0.916618	0.917937
712	0.779054	0.913629				0.8928				0.657761	0.704101	0.735178	0.757467	0.774232	0.787302	0.797776	0.806359	0.813519	0.819584	0.824787	0.8293	0.833251	0.836739	0.839841	0.842618	0.845118	0.847381
713	1.244091	1.069977				1.083129				1.498307	1.367283	1.299119	1.257331	1.229092	1.208731	1.193355	1.181333	1.171675	1.163747	1.157122	1.151504	1.146678	1.142488	1.138817	1.135574	1.132687	1.130102
714	0.819944	0.931794				0.886451				0.643057	0.690451	0.722397	0.745388	0.762727	0.77627	0.787141	0.796059	0.803508	0.809822	0.815243	0.819948	0.82407	0.82771	0.830949	0.83385	0.836462	0.838827
715	1.045537	1.014732				1.002066				1.009013	1.007267	1.006222	1.005527	1.005031	1.004659	1.004371	1.00414	1.003951	1.003793	1.00366	1.003546	1.003447	1.003361	1.003285	1.003217	1.003156	1.003101
716	1.177104	1.0528				1.033503				1.163433	1.127981	1.107729	1.094626	1.085455	1.078677	1.073464	1.069329	1.06597	1.063187	1.060843	1.058842	1.057115	1.055607	1.054281	1.053105	1.052055	1.051111
717	0.824899	0.933919				0.912306				0.705948	0.748261	0.776174	0.79597	0.810739	0.82218	0.831305	0.838752	0.844945	0.850176	0.854654	0.858529	0.861916	0.864902	0.867553	0.869924	0.872056	0.873984
718	1.25567	1.072813				1.071476				1.406601	1.304592	1.250192	1.216378	1.193323	1.176598	1.16391	1.153955	1.145936	1.139338	1.133814	1.129122	1.125086	1.121579	1.118502	1.115782	1.113359	1.111187
719	0.955671	0.984774				0.973231				0.893504	0.912186	0.923774	0.931665	0.937384	0.94172	0.94512	0.947858	0.95011	0.951995	0.953595	0.954971	0.956167	0.957216	0.958144	0.95897	0.95971	0.960377
720	1.019912	1.00655				0.983364				0.931699	0.944099	0.951699	0.956834	0.960536	0.963332	0.965517	0.967273	0.968714	0.969918	0.970939	0.971816	0.972578	0.973245	0.973834	0.974359	0.974829	0.975252
721	0.776973	0.912674				0.916932				0.718095	0.759256	0.786299	0.805423	0.819663	0.830678	0.839452	0.846606	0.85255	0.857568	0.86186	0.865573	0.868818	0.871676	0.874214	0.876483	0.878523	0.880366
722	1.037513	1.012199				1.029573				1.142164	1.111769	1.094297	1.08295	1.074988	1.069094	1.064553	1.060949	1.058018	1.055587	1.05354	1.051791	1.05028	1.048961	1.047801	1.046771	1.045852	1.045025
723	0.838387	0.939624				0.929815				0.753529	0.791022	0.815364	0.832441	0.845084	0.854821	0.862551	0.868837	0.874048	0.878438	0.882188	0.885427	0.888254	0.890743	0.89295	0.894921	0.896692	0.898292
724	0.963664	0.987587				0.971167				0.886012	0.905869	0.918217	0.926637	0.932747	0.937382	0.941019	0.94395	0.946361	0.948379	0.950094	0.951569	0.952851	0.953975	0.95497	0.955855	0.956649	0.957365
725	1.199198	1.058615				1.074295				1.427919	1.319347	1.261783	1.226119	1.201854	1.184277	1.170957	1.160515	1.152109	1.145197	1.139412	1.1345	1.130278	1.126608	1.12339	1.120545	1.118012	1.115742
726	1.162599	1.048899				1.068874				1.387393	1.2912	1.239631	1.207481	1.185519	1.169565	1.15745	1.147937	1.14027	1.133958	1.128671	1.124179	1.120315	1.116955	1.114007	1.1114	1.109078	1.106996
727	1.108958	1.03386				1.028175				1.134745	1.106084	1.089572	1.078835	1.071294	1.065707	1.061402	1.057983	1.055202	1.052896	1.050952	1.049292	1.047857	1.046605	1.045502	1.044524	1.043651	1.042866
728	0.857357	0.947456				0.987126				0.946507	0.956345	0.962347	0.96639	0.969298	0.971492	0.973204	0.974579	0.975706	0.976648	0.977446	0.978131	0.978725	0.979246	0.979706	0.980116	0.980482	0.980812
729	0.890893	0.960778				0.966574				0.869674	0.892031	0.906005	0.915567	0.922522	0.927808	0.931961	0.93531	0.938068	0.940379	0.942344	0.944034	0.945504	0.946794	0.947935	0.948952	0.949863	0.950685
730	0.824155	0.933601				0.900232				0.675566	0.72052	0.750483	0.771883	0.787931	0.800412	0.810397	0.818565	0.825372	0.831132	0.836068	0.840346	0.84409	0.847393	0.850329	0.852955	0.855319	0.857458
731	0.960604	0.986514				1.012056				1.054429	1.043506	1.037061	1.032808	1.029791	1.02754	1.025797	1.024406	1.023271	1.022326	1.021529	1.020846	1.020256	1.019739	1.019284	1.01888	1.018518	1.018193
732	1.04121	1.01337				0.985739				0.941007	0.951805	0.958404	0.962854	0.966058	0.968475	0.970363	0.971879	0.973123	0.974162	0.975043	0.975799	0.976456	0.977031	0.977539	0.977991	0.978396	0.97876
733	0.895732	0.962648				0.969486				0.879981	0.900772	0.913724	0.922568	0.928991	0.933867	0.937695	0.94078	0.943319	0.945445	0.947252	0.948807	0.950158	0.951343	0.952392	0.953325	0.954163	0.954917
734	0.94694	0.981665				1.00875				1.039055	1.031309	1.026717	1.023678	1.021519	1.019905	1.018653	1.017654	1.016838	1.016159	1.015585	1.015094	1.014669	1.014297	1.013969	1.013677	1.013417	1.013182
735	1.180978	1.053831				1.079061				1.465191	1.344879	1.28173	1.242825	1.216452	1.197394	1.18298	1.171696	1.162622	1.155168	1.148934	1.143645	1.139099	1.135152	1.131692	1.128633	1.125911	1.123472
736	1.151617	1.0459				1.012782				1.057854	1.046214	1.039352	1.034828	1.03162	1.029227	1.027373	1.025895	1.024689	1.023686	1.022839	1.022114	1.021487	1.020938	1.020455	1.020026	1.019642	1.019297
737	1.334594	1.09119				1.096362				1.615163	1.444301	1.3581	1.306131	1.27138	1.246507	1.227824	1.213275	1.201627	1.192089	1.184135	1.177402	1.171629	1.166623	1.162242	1.158375	1.154937	1.15186
738	0.961023	0.986661				0.970931				0.885163	0.905153	0.917586	0.926066	0.93222	0.936889	0.940553	0.943505	0.945934	0.947968	0.949696	0.951182	0.952473	0.953607	0.954608	0.955501	0.956301	0.957022
739	1.151121	1.045763				1.004005				1.017589	1.014159	1.012112	1.010751	1.009782	1.009056	1.008493	1.008042	1.007674	1.007367	1.007107	1.006885	1.006693	1.006524	1.006376	1.006244	1.006126	1.006019
740	0.909897	0.968046				1.023025				1.108072	1.085511	1.072411	1.063852	1.057821	1.053343	1.049886	1.047136	1.044897	1.043039	1.041472	1.040132	1.038974	1.037963	1.037072	1.036281	1.035575	1.03494
741	1.156838	1.04733				1.062336				1.340969	1.258451	1.213642	1.1855	1.166185	1.152107	1.14139	1.132959	1.126153	1.120544	1.115841	1.111841	1.108397	1.105402	1.102772	1.100445	1.098371	1.096511
742	1.119577	1.036916				1.04719				1.24266	1.187256	1.156323	1.136582	1.122888	1.112833	1.105136	1.099054	1.094128	1.090056	1.086635	1.083719	1.081204	1.079014	1.077089	1.075383	1.073861	1.072495
743	0.943848	0.980555				0.937963				0.777239	0.812025	0.834432	0.85007	0.861604	0.870462	0.877479	0.883174	0.887889	0.891857	0.895242	0.898164	0.900712	0.902953	0.90494	0.906713	0.908306	0.909744
744	0.84222	0.941224				0.947739				0.807135	0.838224	0.858055	0.871804	0.881899	0.889624	0.895727	0.90067	0.904755	0.908187	0.911112	0.913634	0.915832	0.917763	0.919473	0.920999	0.922369	0.923605
745	0.826059	0.934414				0.939475				0.781757	0.816005	0.838032	0.85339	0.864709	0.873398	0.880277	0.885859	0.890479	0.894366	0.897682	0.900543	0.903038	0.905232	0.907177	0.908913	0.910472	0.91188
746	1.103608	1.032305				1.050431				1.262693	1.201972	1.168264	1.146823	1.131983	1.121104	1.112785	1.106218	1.100903	1.096513	1.092825	1.089683	1.086976	1.084617	1.082545	1.080709	1.079072	1.077603
747	0.969683	0.989686				1.004295				1.01888	1.015194	1.012995	1.011535	1.010494	1.009715	1.00911	1.008626	1.008231	1.007902	1.007623	1.007385	1.007178	1.006998	1.006838	1.006696	1.00657	1.006456
748	1.346971	1.09393				1.073825				1.424327	1.316868	1.259839	1.224487	1.200426	1.182992	1.169779	1.159419	1.151077	1.144218	1.138477	1.133602	1.12941	1.125768	1.122574	1.11975	1.117235	1.114981
749	1.279557	1.078547				1.035667				1.175413	1.137056	1.115221	1.101124	1.091271	1.083996	1.078405	1.073973	1.070374	1.067393	1.064884	1.062742	1.060893	1.059281	1.057862	1.056604	1.055481	1.054472
750	1.150136	1.045492				1.047858				1.246747	1.190267	1.158771	1.138683	1.124756	1.114532	1.106708	1.100527	1.095521	1.091384	1.087908	1.084946	1.082392	1.080167	1.078211	1.076479	1.074934	1.073547
751	0.954609	0.984397				0.980346				0.920068	0.934431	0.943267	0.949251	0.953571	0.956838	0.959394	0.961449	0.963137	0.964548	0.965745	0.966773	0.967666	0.968449	0.969141	0.969757	0.970308	0.970805
752	1.035279	1.011489				1.051273				1.267986	1.205842	1.171396	1.149505	1.134362	1.123265	1.114783	1.108089	1.102671	1.098197	1.094439	1.091238	1.08848	1.086077	1.083966	1.082097	1.08043	1.078933
753	1.23162	1.06688				1.082038				1.489302	1.361216	1.294421	1.253417	1.225684	1.205677	1.190561	1.178739	1.16924	1.16144	1.15492	1.14939	1.14464	1.140516	1.136902	1.133708	1.130866	1.12832
754	0.817435	0.930712				0.924009				0.737258	0.776492	0.802103	0.820137	0.833523	0.843852	0.852065	0.858751	0.8643	0.86898	0.872979	0.876436	0.879455	0.882113	0.884472	0.88658	0.888474	0.890186
755	0.953389	0.983965				0.968416				0.876172	0.897545	0.910877	0.919987	0.926607	0.931635	0.935583	0.938766	0.941386	0.943581	0.945446	0.94705	0.948445	0.949669	0.950752	0.951716	0.952581	0.95336
756	0.934711	0.977247				1.007953				1.0354	1.028399	1.024244	1.021492	1.019536	1.018073	1.016939	1.016033	1.015293	1.014677	1.014157	1.013711	1.013325	1.012988	1.01269	1.012426	1.012189	1.011977
757	0.90468	0.96607				1.000463				1.00201	1.001623	1.001391	1.001236	1.001126	1.001043	1.000978	1.000927	1.000885	1.00085	1.00082	1.000794	1.000772	1.000753	1.000736	1.000721	1.000707	1.000695
758	0.861684	0.949211				0.937381				0.775509	0.810499	0.833051	0.848796	0.860412	0.869335	0.876403	0.882142	0.886893	0.890892	0.894304	0.897249	0.899817	0.902076	0.904079	0.905867	0.907473	0.908923
759	0.98667	0.995517				1.002647				1.011574	1.009328	1.007985	1.007091	1.006454	1.005976	1.005605	1.005309	1.005066	1.004864	1.004693	1.004547	1.00442	1.004309	1.004211	1.004124	1.004046	1.003976
760	1.11932	1.036843				1.036707				1.18124	1.141456	1.118846	1.104264	1.094079	1.086563	1.080788	1.076212	1.072496	1.06942	1.06683	1.064621	1.062713	1.06105	1.059586	1.058289	1.05713	1.05609
761	0.971423	0.990289				0.997756				0.990346	0.992188	0.993297	0.994037	0.994567	0.994964	0.995274	0.995521	0.995724	0.995893	0.996036	0.996159	0.996265	0.996358	0.99644	0.996513	0.996578	0.996637
762	0.85988	0.948481				0.944982				0.798536	0.830721	0.851308	0.865609	0.876122	0.884175	0.890543	0.895703	0.899969	0.903556	0.906613	0.90925	0.911548	0.913568	0.915358	0.916955	0.918389	0.919683
763	1.16244	1.048856				1.076957				1.448544	1.333517	1.27287	1.235414	1.209981	1.191583	1.177656	1.166747	1.15797	1.150756	1.144722	1.1396	1.135198	1.131374	1.128022	1.125058	1.12242	1.120055
764	1.147899	1.044875				1.051107				1.266936	1.205075	1.170775	1.148973	1.133891	1.122837	1.114387	1.107718	1.102321	1.097863	1.094119	1.090931	1.088182	1.085788	1.083685	1.081822	1.080161	1.07867
765	1.055926	1.017972				0.994466				0.976455	0.980896	0.983581	0.985378	0.986667	0.987635	0.988389	0.988994	0.989489	0.989902	0.990251	0.990551	0.990811	0.991039	0.99124	0.991418	0.991578	0.991722
766	0.921362	0.972337				0.978276				0.912219	0.927882	0.937541	0.944093	0.948829	0.952413	0.955219	0.957475	0.95933	0.960881	0.962197	0.963328	0.96431	0.965171	0.965932	0.96661	0.967217	0.967764
767	0.789946	0.91858				0.948352				0.809064	0.839904	0.859563	0.873188	0.883188	0.89084	0.896883	0.901777	0.905821	0.909219	0.912115	0.914611	0.916785	0.918697	0.92039	0.9219	0.923255	0.924478
768	1.00055	1.000183				0.992401				0.967884	0.973899	0.977544	0.979989	0.981743	0.983063	0.984092	0.984916	0.985592	0.986156	0.986633	0.987043	0.987398	0.98771	0.987984	0.988229	0.988447	0.988644
769	1.072325	1.022999				1.017215				1.079139	1.062962	1.053486	1.047263	1.042862	1.039585	1.037051	1.035033	1.033387	1.03202	1.030866	1.029879	1.029025	1.028279	1.027621	1.027037	1.026516	1.026047
770	1.084141	1.026557				1.018259				1.08425	1.066963	1.056853	1.050219	1.045531	1.042042	1.039345	1.037197	1.035447	1.033992	1.032765	1.031715	1.030807	1.030013	1.029314	1.028694	1.028139	1.027641
771	0.835333	0.938342				0.92962				0.752973	0.790527	0.814913	0.832024	0.844692	0.85445	0.862196	0.868495	0.873718	0.878118	0.881877	0.885124	0.887957	0.890451	0.892664	0.89464	0.896415	0.898019
772	1.2905	1.081123				1.066368				1.369294	1.278498	1.229578	1.198994	1.178063	1.162838	1.151266	1.142172	1.134839	1.128799	1.123738	1.119436	1.115735	1.112516	1.109691	1.107192	1.104966	1.102969
773	1.167879	1.050327				1.065075				1.360103	1.272015	1.224435	1.194644	1.174237	1.159383	1.148088	1.139209	1.132046	1.126145	1.121199	1.116995	1.113377	1.11023	1.107468	1.105024	1.102847	1.100895
774	0.971758	0.990405				0.928459				0.749682	0.787596	0.812242	0.829548	0.842368	0.852246	0.86009	0.866471	0.871762	0.876221	0.88003	0.883321	0.886193	0.888722	0.890965	0.892968	0.894768	0.896395
775	1.033977	1.011075				0.966402				0.869072	0.891519	0.905553	0.915157	0.922142	0.927452	0.931624	0.934989	0.93776	0.940082	0.942055	0.943754	0.94523	0.946526	0.947673	0.948694	0.94961	0.950436
776	1.051258	1.016521				1.048891				1.253111	1.194946	1.162569	1.141942	1.127651	1.117165	1.109143	1.102808	1.097679	1.093441	1.08988	1.086846	1.08423	1.081952	1.07995	1.078176	1.076594	1.075174
777	0.957517	0.985426				0.990077				0.958379	0.966112	0.970812	0.97397	0.976239	0.977948	0.979281	0.98035	0.981226	0.981958	0.982578	0.98311	0.983571	0.983975	0.984332	0.98465	0.984934	0.98519
778	0.989194	0.996372				0.979387				0.91642	0.931391	0.94061	0.946858	0.951372	0.954786	0.957458	0.959607	0.961372	0.962848	0.9641	0.965176	0.966111	0.96693	0.967654	0.968299	0.968876	0.969396
779	0.866755	0.951255				0.95671				0.836065	0.863281	0.880478	0.892328	0.90099	0.907597	0.912804	0.917012	0.920484	0.923398	0.925878	0.928014	0.929874	0.931507	0.932952	0.934241	0.935397	0.93644
780	0.932816	0.976555				0.976634				0.906062	0.922731	0.93303	0.940025	0.945086	0.948917	0.951919	0.954334	0.956319	0.95798	0.959389	0.960601	0.961653	0.962576	0.963392	0.964118	0.964769	0.965355
781	0.646337	0.845742				0.824032				0.519383	0.572277	0.60952	0.637165	0.658497	0.675459	0.689267	0.700727	0.710391	0.71865	0.72579	0.732023	0.737513	0.742385	0.746737	0.750649	0.754184	0.757394
782	1.328469	1.089821				1.106268				1.713086	1.506497	1.404847	1.344373	1.30427	1.275728	1.254378	1.237806	1.224569	1.213753	1.204749	1.197137	1.190617	1.18497	1.180032	1.175677	1.171807	1.168346
783	1.109292	1.033957				0.989904				0.957673	0.965532	0.97031	0.973522	0.975829	0.977566	0.978922	0.980009	0.9809	0.981644	0.982275	0.982816	0.983285	0.983696	0.984059	0.984382	0.984671	0.984931
784	0.836933	0.939015				0.926455				0.74405	0.782569	0.807656	0.825294	0.838371	0.848455	0.856467	0.862986	0.868394	0.872953	0.876849	0.880215	0.883154	0.885741	0.888037	0.890087	0.89193	0.893595
785	1.127272	1.039106				1.043145				1.218367	1.169266	1.141659	1.123968	1.111664	1.102611	1.095671	1.090181	1.085731	1.082049	1.078954	1.076315	1.074038	1.072054	1.070309	1.068763	1.067383	1.066145
786	0.992172	0.997377				0.977919				0.910875	0.926759	0.936559	0.943207	0.948014	0.951652	0.954501	0.956792	0.958675	0.960249	0.961586	0.962735	0.963732	0.964607	0.96538	0.966068	0.966685	0.96724
787	0.970596	0.990003				0.991663				0.96485	0.971416	0.975399	0.978073	0.979991	0.981435	0.982561	0.983464	0.984204	0.984821	0.985344	0.985793	0.986182	0.986523	0.986823	0.987091	0.987331	0.987546
788	1.614043	1.145229				1.175794				2.83994	2.097701	1.813343	1.66305	1.570099	1.50693	1.461206	1.426577	1.399442	1.377606	1.359654	1.344636	1.331885	1.320925	1.311404	1.303054	1.295673	1.289102
789	1.222495	1.064585				1.058438				1.31449	1.239525	1.198515	1.172649	1.154847	1.141846	1.131935	1.124129	1.117822	1.11262	1.108256	1.104543	1.101344	1.098561	1.096117	1.093953	1.092025	1.090295
790	1.273465	1.077099				1.091856				1.573706	1.417334	1.337588	1.289229	1.256774	1.233486	1.21596	1.202295	1.19134	1.182363	1.174871	1.168525	1.16308	1.158358	1.154223	1.150571	1.147324	1.144417
791	0.892166	0.961271				0.940777				0.785678	0.819452	0.841148	0.856261	0.867393	0.875934	0.882694	0.888177	0.892715	0.896532	0.899787	0.902596	0.905045	0.907198	0.909107	0.910811	0.91234	0.913721
792	0.924253	0.973408				0.946921				0.804567	0.835986	0.856044	0.869959	0.880179	0.888002	0.894184	0.899192	0.903331	0.90681	0.909774	0.912331	0.914558	0.916515	0.91825	0.919797	0.921186	0.922439
793	1.011887	1.003931				1.007628				1.033915	1.027215	1.023237	1.020602	1.018728	1.017327	1.01624	1.015372	1.014663	1.014073	1.013575	1.013147	1.012778	1.012454	1.012169	1.011916	1.011689	1.011485
794	0.821643	0.932524				0.895871				0.665037	0.710826	0.741456	0.763386	0.779861	0.792692	0.802968	0.811382	0.818398	0.824339	0.829433	0.83385	0.837717	0.841129	0.844163	0.846879	0.849323	0.851536
795	0.849482	0.944231				0.926616				0.744502	0.782973	0.808025	0.825636	0.838693	0.84876	0.856759	0.863267	0.868666	0.873217	0.877105	0.880465	0.883399	0.885981	0.888273	0.890319	0.892159	0.893821
796	1.084474	1.026657				0.990566				0.960366	0.967742	0.972222	0.975232	0.977394	0.979021	0.980291	0.981309	0.982143	0.982839	0.98343	0.983936	0.984375	0.98476	0.985099	0.985402	0.985672	0.985916
797	0.875574	0.954773				0.917986				0.720904	0.761791	0.788628	0.807595	0.821711	0.832626	0.841318	0.848404	0.854291	0.859259	0.863508	0.867184	0.870395	0.873225	0.875737	0.877981	0.88	0.881824
798	1.258401	1.073476				1.047984				1.247519	1.190835	1.159232	1.139079	1.125108	1.114852	1.107004	1.100804	1.095783	1.091634	1.088148	1.085177	1.082616	1.080384	1.078423	1.076686	1.075136	1.073745
799	0.857386	0.947467				0.912993				0.707735	0.749882	0.777669	0.797366	0.812058	0.823438	0.832511	0.839915	0.846072	0.851271	0.855721	0.859573	0.862939	0.865906	0.868541	0.870897	0.873015	0.874931
800	0.958265	0.98569				0.979414				0.916524	0.931477	0.940685	0.946926	0.951435	0.954844	0.957513	0.959659	0.961422	0.962896	0.964147	0.965222	0.966155	0.966973	0.967696	0.96834	0.968917	0.969436
801	0.655265	0.850798				0.85156				0.569681	0.621077	0.656622	0.682667	0.702574	0.718282	0.730994	0.741492	0.750309	0.757818	0.764289	0.769925	0.774878	0.779263	0.783175	0.786684	0.789851	0.792724
802	1.346487	1.093823				1.104953				1.699519	1.498003	1.398509	1.33921	1.299842	1.271803	1.250816	1.23452	1.221499	1.210856	1.201994	1.194501	1.188082	1.182521	1.177658	1.173369	1.169558	1.166148
803	1.190927	1.056456				1.034666				1.169843	1.132842	1.111744	1.09811	1.088574	1.08153	1.076114	1.071821	1.068333	1.065444	1.063012	1.060935	1.059143	1.057579	1.056203	1.054983	1.053894	1.052915
804	1.122833	1.037845				1.01476				1.06727	1.05364	1.045627	1.040353	1.036618	1.033835	1.03168	1.029963	1.028562	1.027397	1.026414	1.025573	1.024845	1.024209	1.023648	1.02315	1.022705	1.022305
805	0.746498	0.898314				0.890821				0.653128	0.69981	0.731166	0.753678	0.770627	0.783847	0.794447	0.803136	0.810387	0.816531	0.821803	0.826376	0.830381	0.833917	0.837063	0.839878	0.842414	0.844709
806	0.827797	0.935155				0.95068				0.816454	0.846327	0.865324	0.878469	0.888106	0.895474	0.901289	0.905996	0.909884	0.913149	0.915931	0.918328	0.920416	0.922251	0.923876	0.925326	0.926626	0.9278
807	1.17228	1.051511				1.024132				1.113719	1.089884	1.076067	1.067048	1.060698	1.055985	1.052349	1.049457	1.047103	1.04515	1.043502	1.042094	1.040877	1.039815	1.038879	1.038049	1.037307	1.03664
808	1.065965	1.021062				1.051535				1.269638	1.207048	1.172371	1.15034	1.135103	1.123938	1.115404	1.10867	1.103221	1.09872	1.094941	1.091722	1.088948	1.086531	1.084409	1.082528	1.080852	1.079347
809	1.070175	1.022346				1.029044				1.139349	1.109614	1.092506	1.081391	1.073589	1.067811	1.06336	1.059826	1.056952	1.054568	1.05256	1.050845	1.049363	1.048069	1.046931	1.045921	1.045019	1.044208
810	1.243156	1.069746				1.079903				1.471943	1.349468	1.285301	1.245808	1.219054	1.19973	1.185118	1.173683	1.16449	1.156938	1.150624	1.145267	1.140664	1.136667	1.133163	1.130067	1.127311	1.124842

	A	B	C	D	E	F	G	H	I	J	K	L	M	N	O	P	Q	R	S	T	U	V	W	X	Y	Z	AA
811	0.928533	0.974986				0.955632				0.83251	0.860217	0.877745	0.889832	0.898672	0.905418	0.910735	0.915034	0.918582	0.921559	0.924094	0.926277	0.928178	0.929847	0.931326	0.932644	0.933826	0.934892
812	1.109341	1.033971				1.025218				1.119306	1.094201	1.079672	1.070198	1.063532	1.058587	1.054772	1.051741	1.049273	1.047225	1.045499	1.044024	1.042748	1.041635	1.040655	1.039785	1.039008	1.03831
813	0.924939	0.973662				0.963194				0.857935	0.882033	0.897152	0.907523	0.915078	0.920828	0.92535	0.929	0.932008	0.93453	0.936674	0.93852	0.940125	0.941535	0.942782	0.943893	0.944889	0.945788
814	1.058847	1.018875				0.987689				0.948757	0.958199	0.963956	0.967832	0.970619	0.972721	0.974362	0.975678	0.976758	0.97766	0.978424	0.97908	0.979649	0.980148	0.980588	0.98098	0.981331	0.981647
815	1.001864	1.000621				1.015586				1.07124	1.056762	1.048262	1.042671	1.038713	1.035765	1.033483	1.031665	1.030182	1.02895	1.027909	1.027019	1.026249	1.025576	1.024983	1.024456	1.023986	1.023562
816	0.686276	0.867769				0.876423				0.620729	0.669565	0.702738	0.726741	0.744916	0.759155	0.770611	0.780029	0.787907	0.794594	0.800343	0.805336	0.809714	0.813585	0.817031	0.820118	0.822901	0.825421
817	1.000834	1.000278				0.999063				0.995952	0.996728	0.997194	0.997505	0.997727	0.997894	0.998024	0.998128	0.998213	0.998283	0.998343	0.998395	0.998439	0.998478	0.998513	0.998543	0.99857	0.998595
818	0.930288	0.97563				0.932676				0.761732	0.798311	0.821995	0.838581	0.850844	0.860279	0.867763	0.873845	0.878884	0.883129	0.886753	0.889882	0.892613	0.895015	0.897146	0.899049	0.900758	0.902302
819	1.033272	1.01085				1.043102				1.218112	1.169076	1.141504	1.123834	1.111544	1.102502	1.09557	1.090087	1.085641	1.081964	1.078872	1.076236	1.073962	1.07198	1.070237	1.068692	1.067314	1.066077
820	1.103133	1.032166				1.014094				1.064087	1.051132	1.04351	1.038489	1.034933	1.032281	1.030228	1.028592	1.027257	1.026147	1.02521	1.024408	1.023714	1.023107	1.022573	1.022098	1.021673	1.021292
821	1.097992	1.030661				0.986242				0.942997	0.953449	0.959832	0.964135	0.967232	0.969568	0.971393	0.972858	0.974059	0.975063	0.975914	0.976645	0.977279	0.977834	0.978325	0.978761	0.979152	0.979504
822	1.387882	1.102729				1.096176				1.613413	1.443171	1.357244	1.305426	1.270772	1.245965	1.227331	1.21282	1.2012	1.191685	1.183751	1.177034	1.171275	1.166281	1.16191	1.158052	1.154621	1.151551
823	1.227139	1.065756				1.085961				1.522096	1.383217	1.311423	1.267562	1.237987	1.216696	1.200636	1.18809	1.178018	1.169755	1.162853	1.157001	1.151977	1.147617	1.143797	1.140423	1.137421	1.134732
824	0.900843	0.964608				0.979717				0.917674	0.932437	0.941524	0.947681	0.952129	0.955492	0.958125	0.960241	0.96198	0.963433	0.964667	0.965726	0.966647	0.967453	0.968166	0.968801	0.969369	0.969881
825	0.80222	0.92406				0.929065				0.751397	0.789124	0.813635	0.830839	0.84358	0.853396	0.861189	0.867527	0.872783	0.877211	0.880993	0.884261	0.887113	0.889624	0.891851	0.89384	0.895628	0.897242
826	0.783051	0.915456				0.911382				0.703555	0.746089	0.77417	0.794096	0.808968	0.820493	0.829687	0.837191	0.843433	0.848706	0.853219	0.857127	0.860542	0.863553	0.866227	0.868618	0.870768	0.872713
827	1.401371	1.105548				1.111396				1.767823	1.540374	1.429984	1.364781	1.321732	1.291187	1.268388	1.250721	1.236628	1.225124	1.215556	1.207473	1.200554	1.194564	1.189329	1.184714	1.180615	1.176949
828	0.972116	0.990529				0.995503				0.980801	0.984436	0.986629	0.988097	0.989149	0.989938	0.990554	0.991046	0.99145	0.991786	0.992071	0.992316	0.992528	0.992713	0.992877	0.993022	0.993152	0.99327
829	1.080988	1.025613				1.029502				1.141787	1.111481	1.094057	1.082741	1.074801	1.068922	1.064394	1.060798	1.057875	1.055451	1.053409	1.051664	1.050157	1.048842	1.047684	1.046657	1.04574	1.044916
830	1.215865	1.062903				1.144282				2.20452	1.789908	1.608409	1.506564	1.441372	1.396064	1.362747	1.337217	1.31703	1.300667	1.287135	1.275759	1.266061	1.257695	1.250405	1.243996	1.238316	1.233249
831	0.952722	0.983728				0.961684				0.852769	0.877618	0.893234	0.903958	0.911776	0.91773	0.922414	0.926196	0.929314	0.931928	0.934151	0.936066	0.937731	0.939193	0.940487	0.94164	0.942674	0.943607
832	0.762469	0.905926				0.879835				0.628208	0.676582	0.709356	0.733029	0.750928	0.764938	0.7762	0.785452	0.793187	0.799751	0.80539	0.810287	0.81458	0.818373	0.82175	0.824775	0.827501	0.829969
833	1.102849	1.032083				1.066663				1.371403	1.279982	1.230755	1.199988	1.178937	1.163627	1.151991	1.142849	1.135476	1.129404	1.124317	1.119993	1.116272	1.113037	1.110198	1.107686	1.105449	1.103442
834	0.698667	0.874305				0.90102				0.677494	0.722291	0.75213	0.773431	0.7894	0.801816	0.811747	0.81987	0.826638	0.832365	0.837272	0.841525	0.845246	0.848529	0.851447	0.854057	0.856406	0.858532
835	0.753162	0.901514				0.906532				0.691186	0.734826	0.763758	0.784347	0.799746	0.811698	0.821243	0.829043	0.835536	0.841025	0.845726	0.849797	0.853358	0.856498	0.859288	0.861783	0.864028	0.866058
836	0.731914	0.891191				0.886725				0.643683	0.691034	0.722943	0.745905	0.763221	0.776744	0.787598	0.796502	0.803938	0.810242	0.815654	0.820351	0.824465	0.828099	0.831332	0.834227	0.836835	0.839196
837	0.863606	0.949988				0.930225				0.754695	0.79206	0.816309	0.833317	0.845906	0.8556	0.863295	0.869552	0.874738	0.879108	0.88284	0.886064	0.888877	0.891353	0.89355	0.895511	0.897274	0.898866
838	0.686093	0.867672				0.860324				0.587018	0.637661	0.672469	0.697866	0.717214	0.732444	0.744744	0.754885	0.763391	0.770626	0.776857	0.782278	0.787038	0.791251	0.795005	0.798373	0.80141	0.804163
839	1.043721	1.014161				0.973003				0.892671	0.911485	0.923158	0.931108	0.936871	0.94124	0.944666	0.947425	0.949695	0.951595	0.953208	0.954595	0.955801	0.956858	0.957793	0.958625	0.959371	0.960044
840	1.245519	1.070328				1.065278				1.361539	1.273029	1.22524	1.195325	1.174836	1.159925	1.148586	1.139673	1.132483	1.126561	1.121597	1.117378	1.113747	1.110588	1.107817	1.105364	1.10318	1.101221
841	1.048422	1.015636				0.988453				0.951818	0.96072	0.966141	0.96979	0.972413	0.974389	0.975932	0.97717	0.978185	0.979033	0.979751	0.980367	0.980902	0.981371	0.981785	0.982153	0.982482	0.982779
842	0.968316	0.989211				0.993733				0.973399	0.978404	0.981432	0.983461	0.984915	0.986009	0.986861	0.987544	0.988103	0.98857	0.988965	0.989304	0.989598	0.989856	0.990083	0.990285	0.990466	0.990629
843	0.867554	0.951576				0.944377				0.796668	0.829087	0.849837	0.864257	0.87486	0.882985	0.889409	0.894617	0.898923	0.902543	0.905629	0.908291	0.91061	0.91265	0.914457	0.91607	0.917517	0.918824
844	1.082026	1.025924				1.00179				1.007802	1.006292	1.005388	1.004787	1.004357	1.004036	1.003786	1.003586	1.003422	1.003286	1.003171	1.003072	1.002986	1.002911	1.002845	1.002787	1.002734	1.002687
845	0.87799	0.955729				0.945201				0.799213	0.831312	0.85184	0.866098	0.876578	0.884606	0.890952	0.896095	0.900347	0.903922	0.906969	0.909597	0.911887	0.9139	0.915684	0.917275	0.918704	0.919993
846	1.263014	1.074592				1.029239				1.140387	1.110409	1.093167	1.081966	1.074106	1.068285	1.063801	1.06024	1.057345	1.054945	1.052922	1.051194	1.049701	1.048399	1.047252	1.046235	1.045326	1.04451
847	1.194877	1.05749				1.094347				1.5964	1.432144	1.348871	1.298536	1.264822	1.240663	1.222502	1.208352	1.197015	1.18773	1.179984	1.173425	1.1678	1.162921	1.15865	1.15488	1.151528	1.148527
848	1.030485	1.009959				1.008887				1.039685	1.03181	1.027143	1.024054	1.02186	1.02022	1.018948	1.017933	1.017104	1.016414	1.015831	1.015332	1.014899	1.014522	1.014188	1.013892	1.013628	1.01339
849	0.910283	0.968192				0.947006				0.804834	0.836219	0.856253	0.870151	0.880358	0.888171	0.894345	0.899346	0.903479	0.906953	0.909913	0.912466	0.91469	0.916645	0.918377	0.919922	0.921309	0.922561
850	1.027595	1.009032				1.045758				1.23397	1.180839	1.151102	1.132095	1.118898	1.109201	1.101774	1.095904	1.091147	1.087215	1.08391	1.081092	1.078663	1.076546	1.074684	1.073035	1.071564	1.070244
851	1.085349	1.026918				1.040908				1.205258	1.15949	1.13366	1.11707	1.105515	1.097004	1.090474	1.085306	1.081114	1.077645	1.074728	1.072239	1.070092	1.06822	1.066574	1.065115	1.063813	1.062644
852	1.033386	1.010886				1.06125				1.333505	1.253135	1.2094	1.181901	1.163013	1.149238	1.138747	1.130492	1.123826	1.118331	1.113723	1.109803	1.106428	1.103492	1.100915	1.098633	1.0966	1.094777
853	1.208789	1.061093				1.036001				1.17728	1.138467	1.116384	1.102132	1.092172	1.08482	1.07917	1.074692	1.071055	1.068044	1.065509	1.063345	1.061478	1.059849	1.058416	1.057145	1.056011	1.054992
854	0.924917	0.973654				0.992063				0.966494	0.972762	0.976562	0.979112	0.980941	0.982318	0.983391	0.984252	0.984957	0.985545	0.986043	0.986471	0.986842	0.987166	0.987453	0.987708	0.987936	0.988142
855	1.041038	1.013315				0.977301				0.908558	0.924821	0.934861	0.941677	0.946606	0.950337	0.953259	0.95561	0.957542	0.959158	0.96053	0.961709	0.962733	0.963631	0.964424	0.965131	0.965764	0.966334
856	0.984773	0.994872				0.989694				0.956824	0.964835	0.969706	0.972981	0.975334	0.977106	0.978489	0.979598	0.980507	0.981266	0.98191	0.982462	0.98294	0.98336	0.98373	0.98406	0.984355	0.98462
857	0.991322	0.99709				1.020913				1.097412	1.077232	1.065477	1.057781	1.052352	1.048317	1.045199	1.042719	1.040698	1.03902	1.037604	1.036394	1.035347	1.034433	1.033628	1.032913	1.032275	1.031701
858	1.198241	1.058366				1.059272				1.320083	1.243537	1.201728	1.175383	1.157261	1.144032	1.13395	1.126012	1.119599	1.114311	1.109875	1.1061	1.10285	1.100021	1.097538	1.09534	1.09338	1.091623
859	0.995303	0.99843				0.995397				0.980357	0.984074	0.986318	0.98782	0.988895	0.989703	0.990333	0.990837	0.99125	0.991594	0.991886	0.992136	0.992352	0.992542	0.99271	0.992859	0.992992	0.993112
860	1.024112	1.00791				1.0081				1.036076	1.028938	1.024702	1.021897	1.019903	1.018413	1.017256	1.016333	1.015579	1.014952	1.014422	1.013967	1.013574	1.01323	1.012927	1.012658	1.012417	1.0122
861	1.150249	1.045523				1.045761				1.233991	1.180855	1.151114	1.132106	1.118908	1.10921	1.101782	1.095912	1.091155	1.087222	1.083916	1.081099	1.078669	1.076551	1.07469	1.073041	1.07157	1.070249
862	1.06714	1.021421				1.050591				1.263696	1.202706	1.168858	1.147332	1.132435	1.121514	1.113164	1.106573	1.101239	1.096832	1.093131	1.089979	1.087261	1.084894	1.082815	1.080973	1.07933	1.077856
863	0.843397	0.941714				0.906643				0.691467	0.735082	0.763995	0.784569	0.799956	0.811898	0.821436	0.829229	0.835716	0.8412	0.845897	0.849965	0.853522	0.856659	0.859446	0.861939	0.864182	0.86621
864	1.009624	1.003187				1.003156				1.013819	1.011132	1.009527	1.008459	1.007698	1.007128	1.006685	1.006331	1.006042	1.0058	1.005596	1.005422	1.00527	1.005138	1.005021	1.004917	1.004824	1.004741
865	1.107022	1.033298				1.011669				1.052612	1.042069	1.035843	1.031734	1.028819	1.026644	1.024958	1.023613	1.022516	1.021603	1.020832	1.020172	1.019601	1.019101	1.018661	1.01827	1.017921	1.017606
866	0.864735	0.950443				0.961566				0.852365	0.877272	0.892927	0.903678	0.911518	0.917487	0.922184	0.925976	0.929102	0.931724	0.933953	0.935873	0.937543	0.939009	0.940307	0.941463	0.9425	0.943435
867	1.160498	1.048328				1.019544				1.090592	1.071918	1.061017	1.053872	1.048827	1.045075	1.042175	1.039867	1.037986	1.036424	1.035106	1.033978	1.033003	1.032152	1.031401	1.030735	1.03014	1.029605
868	0.983223	0.994344				1.01399				1.063586	1.050738	1.043177	1.038196	1.034667	1.032037	1.03	1.028376	1.027051	1.02595	1.02502	1.024224	1.023535	1.022933	1.022403	1.021932	1.021511	1.021132
869	1.045396	1.014687				1.014432				1.0657	1.052403	1.044583	1.039434	1.035787	1.033069	1.030964	1.029287	1.027918	1.026781	1.02582	1.024998	1.024287	1.023666	1.023118	1.022631	1.022197	1.021805
870	0.795776	0.921196				0.900283				0.67569	0.720634	0.750589	0.771983	0.788026	0.800503	0.810484	0.818649	0.825454	0.831211	0.836146	0.840422	0.844164	0.847466	0.850401	0.853026	0.85539	0.857528
871	1.150686	1.045643				1.053381				1.281386	1.215606	1.179283	1.15625	1.140341	1.128694	1.119798	1.112782	1.107107	1.102421	1.098487	1.095138	1.092251	1.089738	1.08753	1.085575	1.083831	1.082267
872	0.941626	0.979754				0.966841				0.870613	0.892829	0.906711	0.916208	0.923114	0.928362	0.932486	0.935811	0.938549	0.940844	0.942794	0.944472	0.945931	0.947211	0.948344	0.949353	0.950257	0.951073
873	1.060184	1.019287				1.070237				1.397398	1.298187	1.245146	1.21213	1.189598	1.173242	1.160828	1.151085	1.143234	1.136772	1.131362	1.126765	1.122812	1.119375	1.11636	1.113693	1.111318	1.109189
874	0.701537	0.875799				0.84912				0.564975	0.616556	0.652286	0.6785	0.698552	0.714387	0.727208	0.737801	0.7467	0.754282	0.760818	0.766512	0.771516	0.775948	0.779902	0.78345	0.786652	0.789556
875	0.990766	0.996903				1.008284				1.036918	1.029608	1.025272	1.022401	1.02036	1.018835	1.017652	1.016707	1.015936	1.015293	1.014751	1.014286	1.013884	1.013532	1.013222	1.012946	1.0127	1.012478
876	1.115368	1.03571				1.052843				1.277945	1.213103	1.177263	1.154524	1.138812	1.127306	1.118516	1.111582	1.105973	1.101342	1.097453	1.094141	1.091288	1.088803	1.08662	1.084686	1.082963	1.081416
877	1.161942	1.048721				1.083767				1.50361	1.370846	1.301876	1.259626	1.231088	1.210519	1.19499	1.182851	1.173101	1.165098	1.158411	1.15274	1.147869	1.143642	1.139937	1.136664	1.133752	1.131143
878	0.926612	0.974279				0.972725				0.891657	0.91063	0.922407	0.930428	0.936244	0.940654	0.944112	0.946898	0.949189	0.951107	0.952735	0.954136	0.955353	0.95642	0.957364	0.958205	0.958958	0.959637
879	0.669439	0.858667				0.898929				0.672397	0.717607	0.747774	0.769334	0.785512	0.798099	0.808171	0.816414	0.823284	0.829098	0.834082	0.838402	0.842183	0.845519	0.848484	0.851138	0.853526	0.855687
880	0.862025	0.949349				0.93925				0.781081	0.81541	0.837495	0.852895	0.864246	0.87296	0.87986	0.885459	0.890093	0.893992	0.897318	0.900188	0.902691	0.904893	0.906844	0.908585	0.910149	0.911561
881	1.160278	1.048268				1.062025				1.338825	1.256925	1.212425	1.184468	1.165276	1.151284	1.140633	1.132252	1.125486	1.119909	1.115234	1.111257	1.107833	1.104855	1.10224	1.099926	1.097864	1.096014
882	0.993063	0.997677				0.99977				0.999005	0.999196	0.999311	0.999387	0.999442	0.999483	0.999515	0.99954	0.999561	0.999579	0.999593	0.999606	0.999617	0.999627	0.999635	0.999642	0.999649	0.999655
883	1.161151	1.048506				1.009291				1.041546	1.033291	1.0284	1.025165	1.022866	1.02115	1.019818	1.018756	1.017888	1.017166	1.016556	1.016033	1.015581	1.015185	1.014837	1.014527	1.01425	1.014001
884	0.911581	0.968681				0.944115				0.795859	0.828379	0.849199	0.863671	0.874313	0.882469	0.888918	0.894145	0.898468	0.902103	0.905201	0.907874	0.910203	0.912251	0.914066	0.915685	0.917139	0.918451
885	1.263087	1.07461				1.05904				1.318528	1.242423	1.200836	1.174624	1.156591	1.143425	1.133391	1.125489	1.119106	1.113842	1.109425	1.105668	1.102432	1.099616	1.097144	1.094955	1.093004	1.091254
886	1.158778	1.04786				1.06673				1.371883	1.28032	1.231022	1.200214	1.179135	1.163806	1.152156	1.143003	1.135621	1.129542	1.124449	1.12012	1.116395	1.113156	1.110313	1.107799	1.105558	1.10355
887	1.086954	1.027397				1.076129				1.442073	1.329082	1.269405	1.232511	1.207445	1.189304	1.175567	1.164804	1.156143	1.149023	1.143067	1.138011	1.133665	1.129889	1.126579	1.123652	1.121047	1.118712
888	0.997967	0.999321				0.962308				0.854898	0.879438	0.89485	0.905429	0.913139	0.919009	0.923626	0.927354	0.930426	0.933002	0.935193	0.937079	0.93872	0.94016	0.941435	0.942571	0.943589	0.944508
889	0.742243	0.896253				0.838874				0.545757	0.597994	0.634429	0.66129	0.681912	0.698243	0.711496	0.722467	0.731697	0.739571	0.746368	0.752293	0.757505	0.762126	0.766249	0.769953	0.773297	0.776331
890	0.743245	0.89674				0.922314				0.732605	0.772319	0.798284	0.816586	0.830181	0.840679	0.849029	0.855829	0.861474	0.866236	0.870307	0.873826	0.8769	0.879607	0.882009	0.884156	0.886085	0.887829
891	0.879269	0.956234				0.90987				0.699668	0.742555	0.770907	0.791043	0.806082	0.817742	0.827047	0.834644	0.840965	0.846306	0.850879	0.854837	0.858298	0.86135	0.86406	0.866484	0.868664	0.870635
892	0.817965	0.930941				0.919772				0.725701	0.766113	0.792595	0.811292	0.825195	0.83594	0.844492	0.851461	0.857249	0.862133	0.866309	0.869921	0.873075	0.875855	0.878322	0.880526	0.882508	0.884299
893	0.888883	0.959998				0.944905				0.798298	0.830513	0.851121	0.865437	0.875961	0.884024	0.890398	0.895564	0.899836	0.903427	0.906488	0.909128	0.911429	0.913451	0.915243	0.916842	0.918278	0.919573
894	0.986435	0.995437				0.966183				0.868304	0.890866	0.904975	0.914632	0.921657	0.926997	0.931194	0.934578	0.937365	0.939701	0.941687	0.943395	0.944881	0.946184	0.947338	0.948366	0.949287	0.950118
895	1.045232	1.014636				1.035396				1.173902	1.135914	1.114279	1.100307	1.09054	1.083328	1.077784	1.07339	1.069821	1.066865	1.064377	1.062253	1.060419	1.05882	1.057413	1.056165	1.055051	1.054051
896	0.810169	0.927555				0.926206				0.743356	0.781948	0.80709	0.824768	0.837877	0.847986	0.856018	0.862555	0.867977	0.872549	0.876455	0.87983	0.882777	0.885372	0.887674	0.88973	0.891578	0.893248
897	0.785626	0.916627				0.912894				0.707477	0.749648	0.777453	0.797165	0.811868	0.823256	0.832337	0.839747	0.845909	0.851114	0.855567	0.859422	0.862792	0.865761	0.868399	0.870757	0.872877	0.874794
898	0.875994	0.95494				0.968898				0.877886	0.898997	0.912159	0.921149	0.92768	0.93264	0.936534	0.939673	0.942257	0.944421	0.94626	0.947842	0.949217	0.950423	0.951491	0.952441	0.953294	0.954062
899	1.012914	1.004268				0.983847				0.933578	0.945658	0.953056	0.958054	0.961655	0.964374	0.9665	0.968207	0.969608	0.970779	0.971772	0.972624	0.973364	0.974013	0.974586	0.975096	0.975553	0.975964
900	0.834379	0.937941				0.908565				0.696333	0.739519	0.768101	0.788416	0.803597	0.815372	0.824772	0.832449	0.838838	0.844237	0.84886	0.852863	0.856363	0.859449	0.862191	0.864643	0.866848	0.868843
901	0.895517	0.962565				0.980012				0.918797	0.933373	0.942342	0.948418	0.952806	0.956124	0.958721	0.960808	0.962523	0.963956	0.965173	0.966218	0.967125	0.967921	0.968624	0.96925	0.96981	0.970315
902	1.067709	1.021595				1.024398				1.115085	1.090941	1.07695	1.06782	1.061393	1.056623	1.052943	1.050017	1.047635	1.045658	1.043992	1.042567	1.041336	1.040261	1.039314	1.038474	1.037724	1.037049
903	1.094264	1.029563				1.013302				1.060314	1.048157	1.040995	1.036275	1.03293	1.030435	1.028502	1.026962	1.025705	1.02466	1.023777	1.023021	1.022368	1.021796	1.021293	1.020845	1.020446	1.020086
904	1.0279	1.00913				0.988394				0.95158	0.960524	0.965971	0.969637	0.972273	0.974259	0.97581	0.977054	0.978074	0.978926	0.979648	0.980267	0.980805	0.981276	0.981692	0.982062	0.982393	0.982691
905	0.933508	0.976808				0.989316				0.955295	0.963579	0.968619	0.972008	0.974443	0.976278	0.97771	0.978858	0.9798	0.980586	0.981252	0.981823	0.982319	0.982754	0.983137	0.983479	0.983784	0.984059
906	0.8034	0.924582				0.8935				0.659408	0.705626	0.736602	0.75881	0.775511	0.788526	0.798956	0.8075	0.814628	0.820665	0.825844	0.830335	0.834266	0.837737	0.840824	0.843587	0.846075	0.848326
907	0.837164	0.939112				0.9846				0.936525	0.948098	0.95518	0.959961	0.963405	0.966004	0.968036	0.969667	0.971006	0.972124	0.973073	0.973887	0.974594	0.975213	0.975761	0.976248	0.976684	0.977076
908	0.836166	0.938693				0.942884				0.792083	0.825073	0.846219	0.86093	0.871754	0.880053	0.886618	0.89194	0.896343	0.900045	0.903202	0.905925	0.908299	0.910386	0.912235	0.913886	0.915367	0.916705
909	1.03525	1.01148				0.95009				0.814572	0.844693	0.863859	0.877127	0.886857	0.894297	0.90017	0.904925	0.908853	0.912152	0.914962	0.917385	0.919495	0.921349	0.922992	0.924457	0.925771	0.926958
910	1.044977	1.014556				0.976176				0.904356	0.921302	0.931778	0.938895	0.944045	0.947945	0.951001	0.95346	0.955481	0.957172	0.958608	0.959842	0.960914	0.961853	0.962684	0.963424	0.964087	0.964684
911	1.119394	1.036864				1.028714				1.137596	1.10827	1.091389	1.080418	1.072716	1.067011	1.062616	1.059125	1.056286	1.053932	1.051949	1.050254	1.04879	1.047512	1.046388	1.04539	1.044499	1.043698
912	0.83983	0.940227				0.901562				0.678821	0.723509	0.753262	0.774495	0.790409	0.802781	0.812675	0.820767	0.827508	0.833212	0.838099	0.842334	0.84604	0.849309	0.852214	0.854814	0.857153	0.859269
913	0.93653	0.977908				0.972738				0.891706	0.910671	0.922443	0.930461	0.936274	0.940682	0.944139	0.946923	0.949213	0.95113	0.952758	0.954158	0.955375	0.956442	0.957385	0.958225	0.958978	0.959657
914	1.047013	1.015195				0.967913				0.87439	0.896035	0.909543	0.918778	0.925489	0.930588	0.934592	0.937821	0.940479	0.942705	0.944598	0.946226	0.947641	0.948883	0.949982	0.950961	0.951838	0.952629
915	1.027793	1.009096				1.082473				1.49288	1.363629	1.296291	1.254975	1.227041	1.206893	1.191674	1.179772	1.17021	1.162359	1.155798	1.150232	1.145452	1.141302	1.137665	1.134452	1.131592	1.12903
916	0.767172	0.908131				0.927489				0.746948	0.785157	0.810018	0.827485	0.840431	0.850409	0.858334	0.864782	0.87013	0.874638	0.878489	0.881816	0.884721	0.887278	0.889546	0.891573	0.893393	0.895039
917	0.790369	0.918771				0.926026				0.742853	0.781499	0.806679	0.824387	0.837519	0.847646	0.855693	0.862242	0.867675	0.872256	0.876169	0.879552	0.882504	0.885104	0.887411	0.889471	0.891323	0.892997
918	1.067578	1.021555				1.016232				1.074363	1.059216	1.05033	1.044489	1.040357	1.037279	1.034897	1.033	1.031453	1.030167	1.029082	1.028153	1.02735	1.026648	1.026029	1.02548	1.024989	1.024548
919	0.920654	0.972074				0.984206				0.934983	0.946821	0.954069	0.958963	0.96249	0.965152	0.967232	0.968903	0.970275	0.971421	0.972392	0.973227	0.973951	0.974586	0.975146	0.975645	0.976092	0.976495
920	0.773836	0.911227				0.940587				0.785101	0.818946	0.84069	0.855839	0.866999	0.875561	0.882339	0.887837	0.892387	0.896214	0.899478	0.902295	0.90475	0.90691	0.908824	0.910532	0.912066	0.913451
921	0.93234	0.976381				0.979962				0.918604	0.933212	0.942202	0.948292	0.95269	0.956016	0.958618	0.960711	0.96243	0.963867	0.965086	0.966133	0.967043	0.967841	0.968545	0.969173	0.969734	0.970241
922	0.780134	0.914124				0.928696				0.750352	0.788192	0.812786	0.830052	0.842841	0.852695	0.86052	0.866883	0.872161	0.876608	0.880406	0.883688	0.886553	0.889074	0.891311	0.893309	0.895104	0.896726
923	0.763537	0.906428				0.923784				0.736639	0.775938	0.801596	0.819665	0.833079	0.843431	0.851662	0.858364	0.863926	0.868616	0.872625	0.87609	0.879116	0.881781	0.884146	0.886258	0.888157	0.889873
924	0.673312	0.860784				0.89498				0.662915	0.708866	0.739628	0.761663	0.778224	0.791125	0.801458	0.809921	0.81698	0.822957	0.828083	0.832528	0.836419	0.839854	0.842908	0.845641	0.848102	0.850329
925	0.858228	0.94781				0.950963				0.81736	0.847114	0.866029	0.879115	0.888707	0.896039	0.901827	0.906511	0.910379	0.913629	0.916396	0.918782	0.920859	0.922685	0.924301	0.925743	0.927037	0.928205
926	1.083951	1.026501				1.037735				1.187047	1.145831	1.122447	1.107381	1.096865	1.089108	1.08315	1.07843	1.074599	1.071427	1.068758	1.066481	1.064515	1.062801	1.061293	1.059956	1.058763	1.057691
927	0.624842	0.83324				0.836919				0.542185	0.594528	0.631083	0.658057	0.678781	0.695201	0.708532	0.71957	0.728861	0.736788	0.743632	0.7496	0.754851	0.759505	0.76366	0.767392	0.770762	0.773821
928	1.010418	1.003449				0.969733				0.880861	0.901516	0.914381	0.923163	0.92954	0.934381	0.938181	0.941244	0.943764	0.945875	0.947668	0.949211	0.950552	0.951729	0.952769	0.953696	0.954527	0.955276
929	0.842744	0.941442				0.955138				0.830888	0.858818	0.876496	0.888691	0.897612	0.904421	0.909789	0.914129	0.917711	0.920718	0.923277	0.925482	0.927402	0.929088	0.930581	0.931912	0.933106	0.934184
930	0.761765	0.905594				0.935259				0.769253	0.804973	0.828044	0.844173	0.856083	0.865239	0.872497	0.878392	0.883274	0.887385	0.890893	0.893922	0.896564	0.898888	0.900949	0.902789	0.904442	0.905934
931	1.214424	1.062535				1.043565				1.220851	1.171113	1.143168	1.125268	1.112821	1.103666	1.096648	1.091098	1.086599	1.082877	1.079748	1.077081	1.07478	1.072774	1.071011	1.069448	1.068054	1.066802
932	0.961707	0.986901				0.986005				0.942059	0.952674	0.959159	0.963531	0.966679	0.969053	0.970908	0.972397	0.973618	0.974639	0.975504	0.976246	0.976891	0.977456	0.977954	0.978398	0.978796	0.979154
933	0.600238	0.818329				0.83349				0.535996	0.588509	0.625265	0.65243	0.673326	0.689897	0.703361	0.714516	0.72391	0.731929	0.738855	0.744896	0.750212	0.754926	0.759135	0.762916	0.766332	0.769431
934	1.084944	1.026797				1.020314				1.094423	1.074905	1.063524	1.05607	1.05081	1.046898	1.043876	1.041471	1.039512	1.037884	1.036511	1.035337	1.034322	1.033435	1.032654	1.031961	1.031341	1.030784
935	0.824516	0.933755				0.909073				0.697629	0.7407	0.769193	0.789438	0.804564	0.816294	0.825657	0.833304	0.839666	0.845042	0.849646	0.853632	0.857117	0.860189	0.862919	0.86536	0.867555	0.869541
936	0.833913	0.937745				0.896644				0.666888	0.712533	0.743047	0.764885	0.781286	0.794056	0.80428	0.812652	0.819631	0.82554	0.830607	0.835	0.838844	0.842238	0.845255	0.847954	0.850385	0.852584
937	0.908278	0.967435				0.90266				0.681527	0.72599	0.755566	0.77666	0.792463	0.804743	0.814561	0.822589	0.829277	0.834933	0.83978	0.843979	0.847653	0.850894	0.853774	0.856351	0.858669	0.860767
938	1.201161	1.059125				1.065743				1.364841	1.27536	1.22709	1.196889	1.176212	1.161167	1.149729	1.14074	1.133488	1.127516	1.122511	1.118256	1.114595	1.111411	1.108617	1.106144	1.103942	1.101967
939	0.882124	0.957357				0.929839				0.753595	0.791081	0.815417	0.832491	0.845131	0.854866	0.862594	0.868877	0.874087	0.878476	0.882225	0.885464	0.88829	0.890777	0.892984	0.894955	0.896725	0.898325
940	1.197747	1.058238				1.053636				1.283026	1.216798	1.180245	1.157072	1.141069	1.129355	1.120408	1.113353	1.107646	1.102934	1.098979	1.095611	1.092709	1.090182	1.087963	1.085997	1.084244	1.082672
941	1.198702	1.058486				1.073705				1.423412	1.316237	1.259344	1.224071	1.200062	1.182665	1.169478	1.159139	1.150814	1.143968	1.138238	1.133373	1.129189	1.125554	1.122366	1.119547	1.117037	1.114787
942	1.115001	1.035604				1.055527				1.295268	1.225672	1.187392	1.163173	1.14647	1.134254	1.124931	1.117583	1.111642	1.106739	1.102623	1.09912	1.096102	1.093475	1.091168	1.089125	1.087303	1.085669
943	1.077733	1.024635				1.024954				1.117946	1.093151	1.078795	1.069432	1.062843	1.057955	1.054183	1.051186	1.048746	1.046721	1.045014	1.043555	1.042294	1.041193	1.040224	1.039363	1.038595	1.037904
944	0.86084	0.94887				0.917772				0.720334	0.761277	0.788156	0.807155	0.821296	0.832232	0.840941	0.84804	0.853938	0.858917	0.863175	0.866858	0.870076	0.872912	0.875429	0.877678	0.879701	0.881529
945	0.892863	0.961541				0.930843				0.756461	0.79363	0.817738	0.834641	0.847148	0.856778	0.86442	0.870633	0.875782	0.880121	0.883825	0.887026	0.889818	0.892276	0.894456	0.896403	0.898152	0.899732
946	1.300153	1.083369				1.06314				1.346542	1.262412	1.216797	1.188176	1.168543	1.154238	1.143353	1.134791	1.12788	1.122186	1.117412	1.113352	1.109858	1.106818	1.104149	1.101788	1.099684	1.097797
947	1.425117	1.110413				1.080837				1.479497	1.354589	1.28928	1.24913	1.221949	1.202328	1.187496	1.175892	1.166566	1.158906	1.152502	1.147069	1.142402	1.13835	1.134798	1.131659	1.128865	1.126363
948	0.914587	0.96981				0.949244				0.811884	0.842357	0.861765	0.875207	0.885069	0.892612	0.898569	0.903391	0.907376	0.910723	0.913575	0.916034	0.918175	0.920057	0.921725	0.923212	0.924546	0.92575
949	0.750309	0.900148				0.881714				0.632376	0.680484	0.71303	0.736514	0.754259	0.768138	0.779292	0.788451	0.796106	0.8026	0.808178	0.813021	0.817266	0.821017	0.824355	0.827345	0.830039	0.832478
950	1.076794	1.024351				1.043645				1.221331	1.171469	1.143459	1.125519	1.113045	1.103869	1.096837	1.091275	1.086766	1.083037	1.079902	1.077228	1.074923	1.072913	1.071146	1.06958	1.068183	1.066929
951	1.014802	1.004886				1.006878				1.030504	1.024494	1.020922	1.018554	1.016869	1.01561	1.014632	1.013851	1.013213	1.012682	1.012233	1.011849	1.011516	1.011225	1.010968	1.01074	1.010536	1.010353
952	1.017046	1.005618				0.997979				0.9913	0.992961	0.99396	0.994628	0.995105	0.995464	0.995742	0.995966	0.996148	0.996301	0.996429	0.99654	0.996636	0.99672	0.996794	0.996859	0.996918	0.996971
953	1.184086	1.054655				1.05723				1.306459	1.233749	1.193882	1.168705	1.151362	1.138689	1.129024	1.121408	1.115254	1.110176	1.105916	1.10229	1.099167	1.096449	1.094061	1.091948	1.090064	1.088374
954	0.54942	0.78532				0.786902				0.460089	0.513394	0.551749	0.58067	0.603256	0.621383	0.636253	0.648671	0.659199	0.668236	0.676078	0.682949	0.689017	0.694415	0.69925	0.703604	0.707546	0.711132
955	0.889132	0.960094				0.936282				0.772258	0.807629	0.830451	0.846396	0.858166	0.86721	0.874377	0.880197	0.885016	0.889073	0.892535	0.895523	0.89813	0.900423	0.902456	0.904271	0.905901	0.907373
956	1.072222	1.022968				0.992782				0.969456	0.975184	0.978654	0.98098	0.982649	0.983904	0.984883	0.985667	0.98631	0.986846	0.9873	0.987689	0.988027	0.988323	0.988584	0.988817	0.989024	0.989212
957	1.368406	1.098588				1.083354				1.500169	1.368535	1.300088	1.258138	1.229793	1.209359	1.19393	1.181867	1.172177	1.164222	1.157575	1.151938	1.147097	1.142894	1.139211	1.135957	1.133062	1.130468
958	0.972646	0.990713				0.982195				0.927169	0.94034	0.948423	0.95389	0.957833	0.960812	0.963142	0.965014	0.966551	0.967836	0.968925	0.969861	0.970674	0.971386	0.972015	0.972575	0.973077	0.973529
959	1.090952	1.028584				1.022243				1.104103	1.082433	1.069834	1.061597	1.055791	1.051477	1.048147	1.045497	1.04334	1.041548	1.040037	1.038746	1.037629	1.036654	1.035795	1.035033	1.034351	1.033739
960	0.460066	0.718804				0.717012				0.368967	0.419926	0.457869	0.487217	0.510594	0.529654	0.545492	0.558861	0.570296	0.58019	0.588833	0.596449	0.603211	0.609254	0.614689	0.619601	0.624063	0.628135
961	0.921359	0.972336				0.977485				0.909245	0.925396	0.935365	0.942131	0.947024	0.950728	0.953628	0.955961	0.957879	0.959482	0.960844	0.962013	0.96303	0.96392	0.964708	0.965409	0.966037	0.966603
962	0.932033	0.976269				0.990332				0.959413	0.96696	0.971546	0.974627	0.97684	0.978507	0.979807	0.980849	0.981704	0.982417	0.983021	0.98354	0.98399	0.984384	0.984732	0.985041	0.985318	0.985568
963	1.015592	1.005144				0.966895				0.870803	0.89299	0.906853	0.916337	0.923233	0.928474	0.932592	0.935912	0.938647	0.940937	0.942885	0.94456	0.946017	0.947295	0.948426	0.949434	0.950337	0.951152
964	0.857908	0.94768				0.954277				0.82807	0.856385	0.874323	0.886705	0.895766	0.902685	0.90814	0.912552	0.916194	0.919251	0.921854	0.924096	0.926049	0.927764	0.929283	0.930637	0.931852	0.932948
965	1.157229	1.047437				1.054609				1.289303	1.221352	1.183915	1.160206	1.143844	1.131873	1.122733	1.115528	1.1097	1.10489	1.100853	1.097416	1.094454	1.091876	1.089611	1.087606	1.085818	1.084213
966	1.071685	1.022805				1.06018				1.326217	1.24793	1.205242	1.17837	1.159897	1.146419	1.13615	1.128066	1.121538	1.116155	1.11164	1.107799	1.104492	1.101614	1.099087	1.096851	1.094858	1.09307
967	0.99706	0.999018				1.032596				1.158465	1.124206	1.104606	1.091915	1.083027	1.076455	1.071399	1.067388	1.064128	1.061427	1.059152	1.05721	1.055533	1.054069	1.052781	1.051639	1.050619	1.049703
968	0.910043	0.968101				0.946648				0.803716	0.835244	0.855376	0.869346	0.879607	0.887463	0.893671	0.898701	0.902858	0.906352	0.909329	0.911897	0.914134	0.916101	0.917843	0.919397	0.920792	0.922052
969	1.20026	1.058891				1.034037				1.166371	1.130211	1.109571	1.096225	1.086887	1.079987	1.074681	1.070473	1.067055	1.064223	1.061839	1.059804	1.058046	1.056513	1.055164	1.053967	1.052899	1.05194
970	1.079506	1.025168				0.986375				0.943523	0.953883	0.960209	0.964473	0.967542	0.969857	0.971665	0.973116	0.974307	0.975301	0.976144	0.976868	0.977496	0.978046	0.978532	0.978965	0.979352	0.979701
971	1.186185	1.055209				1.054972				1.291653	1.223055	1.185286	1.161376	1.14488	1.132812	1.123601	1.116339	1.110466	1.10562	1.101552	1.098089	1.095105	1.092507	1.090226	1.088205	1.086404	1.084788
972	0.934349	0.977115				0.96262				0.855968	0.880352	0.895661	0.906167	0.913823	0.91965	0.924234	0.927935	0.930984	0.933541	0.935715	0.937587	0.939215	0.940645	0.94191	0.943037	0.944048	0.944959
973	0.917207	0.97079				0.923395				0.735567	0.774976	0.800716	0.818848	0.83231	0.8427	0.850963	0.857691	0.863275	0.867984	0.872009	0.875489	0.878528	0.881204	0.883579	0.8857	0.887607	0.889331
974	1.299801	1.083287				1.072465				1.41402	1.30974	1.254241	1.219784	1.196308	1.179286	1.166377	1.156252	1.148098	1.14139	1.135775	1.131006	1.126905	1.123341	1.120215	1.117451	1.11499	1.112784
975	0.850645	0.94471				0.928601				0.750082	0.787952	0.812567	0.829849	0.842651	0.852514	0.860347	0.866717	0.872	0.876452	0.880255	0.88354	0.886408	0.888932	0.891172	0.893172	0.894969	0.896592
976	1.235006	1.067725				1.086315				1.52512	1.385233	1.312975	1.268851	1.239107	1.217698	1.201551	1.188939	1.178815	1.17051	1.163573	1.157692	1.152643	1.148261	1.144422	1.141031	1.138014	1.135313
977	0.66593	0.856737				0.865168				0.596897	0.647057	0.681414	0.70642	0.725436	0.740383	0.752441	0.762375	0.770699	0.777776	0.783867	0.789164	0.793813	0.797926	0.801591	0.804877	0.80784	0.810525
978	1.170603	1.05106				1.088349				1.542653	1.39688	1.32193	1.276278	1.245553	1.223463	1.206816	1.193821	1.183395	1.174845	1.167706	1.161656	1.156463	1.151957	1.14801	1.144525	1.141424	1.138648
979	0.949658	0.982636				0.98808				0.950322	0.959489	0.965074	0.968834	0.971537	0.973575	0.975165	0.976442	0.977488	0.978362	0.979103	0.979739	0.980291	0.980774	0.981201	0.98158	0.98192	0.982227
980	1.349256	1.094431				1.123605				1.910944	1.626084	1.492586	1.415134	1.364556	1.328933	1.302486	1.282075	1.265845	1.252631	1.241663	1.232413	1.224508	1.217674	1.211706	1.206451	1.201787	1.197621
981	1.119099	1.036779				1.065453				1.362777	1.273903	1.225934	1.195912	1.175352	1.160391	1.149015	1.140073	1.13286	1.126919	1.12194	1.117707	1.114065	1.110897	1.108117	1.105657	1.103466	1.101501
982	1.353786	1.095423				1.055273				1.293615	1.224476	1.186429	1.162352	1.145744	1.133595	1.124324	1.117015	1.111105	1.106227	1.102134	1.098649	1.095647	1.093033	1.090737	1.088705	1.086892	1.085267
983	1.261238	1.074163				1.104677				1.696688	1.496226	1.397181	1.338127	1.298913	1.270979	1.250069	1.23383	1.220854	1.210247	1.201415	1.193947	1.187549	1.182007	1.177159	1.172884	1.169085	1.165687
984	1.066051	1.021088				1.043539				1.220697	1.170998	1.143074	1.125187	1.11275	1.1036	1.096587	1.091041	1.086545	1.082826	1.079699	1.077033	1.074734	1.072729	1.070967	1.069406	1.068012	1.066761
985	0.969624	0.989665				0.965503				0.865928	0.888845	0.903187	0.913009	0.920155	0.92559	0.929861	0.933306	0.936144	0.938522	0.940544	0.942284	0.943797	0.945125	0.9463	0.947346	0.948285	0.949131
986	0.85949	0.948323				0.961235				0.85124	0.876309	0.892072	0.9029	0.910796	0.91681	0.921542	0.925363	0.928513	0.931155	0.933402	0.935336	0.937019	0.938497	0.939805	0.94097	0.942015	0.942958
987	0.985271	0.995042				0.966504				0.86943	0.891823	0.905822	0.915401	0.922368	0.927663	0.931824	0.93518	0.937943	0.940259	0.942227	0.94392	0.945393	0.946685	0.947829	0.948847	0.94976	0.950584
988	0.996115	0.998702				1.007428				1.033005	1.02649	1.02262	1.020056	1.018233	1.01687	1.015812	1.014967	1.014277	1.013703	1.013217	1.012802	1.012442	1.012127	1.011849	1.011603	1.011382	1.011184
989	0.84804	0.943637				0.948992				0.811085	0.841663	0.861142	0.874636	0.884537	0.892111	0.898092	0.902935	0.906936	0.910298	0.913162	0.915631	0.917782	0.919673	0.921347	0.922841	0.924181	0.925391
990	0.850048	0.944464				0.920076				0.72652	0.766851	0.793272	0.811922	0.825789	0.836504	0.845033	0.851982	0.857753	0.862622	0.866786	0.870386	0.873531	0.876302	0.878761	0.880959	0.882935	0.88472
991	0.830334	0.936232				0.937737				0.776566	0.811432	0.833896	0.849576	0.861141	0.870024	0.877061	0.882773	0.887502	0.891482	0.894878	0.897809	0.900364	0.902613	0.904606	0.906385	0.907983	0.909425
992	1.166985	1.050086				1.045088				1.229938	1.177855	1.14867	1.130003	1.117037	1.107507	1.100205	1.094433	1.089756	1.085888	1.082637	1.079865	1.077475	1.075392	1.073561	1.071939	1.070491	1.069192
993	1.175714	1.05243				1.071767				1.408777	1.306103	1.251382	1.217379	1.194201	1.177388	1.164635	1.154631	1.146572	1.139941	1.134391	1.129676	1.125621	1.122097	1.119006	1.116273	1.113838	1.111657
994	0.686221	0.86774				0.876249				0.620352	0.66921	0.702403	0.726423	0.744611	0.758862	0.770328	0.779754	0.787639	0.794333	0.800086	0.805085	0.809467	0.813342	0.816791	0.819882	0.822667	0.82519
995	1.087566	1.027579				1.026103				1.123895	1.09774	1.082624	1.072775	1.06585	1.060714	1.056753	1.053606	1.051045	1.04892	1.047129	1.045599	1.044276	1.043122	1.042105	1.041203	1.040397	1.039673
996	0.945108	0.981008				0.969876				0.881375	0.901951	0.914764	0.92351	0.929861	0.934681	0.938465	0.941514	0.944024	0.946125	0.947911	0.949447	0.950782	0.951953	0.952989	0.953912	0.954739	0.955485
997	0.975005	0.991527				1.030242				1.145738	1.114502	1.096565	1.084925	1.07676	1.070717	1.066063	1.062369	1.059366	1.056876	1.054779	1.052987	1.05144	1.050089	1.048901	1.047846	1.046905	1.046059
998	1.111663	1.034642				1.026636				1.126671	1.099878	1.084405	1.07433	1.067247	1.061996	1.057947	1.05473	1.052113	1.049942	1.048111	1.046548	1.045196	1.044017	1.042978	1.042057	1.041233	1.040494
999	0.8408	0.940632				0.92819				0.748924	0.78692	0.811626	0.828976	0.841831	0.851737	0.859604	0.866003	0.87131	0.875783	0.879603	0.882904	0.885785	0.888322	0.890572	0.892582	0.894388	0.896019
1000	1.221537	1.064343				1.082697				1.494726	1.364873	1.297254	1.255778	1.227739	1.207519	1.192246	1.180304	1.170709	1.162832	1.156249	1.150665	1.14587	1.141706	1.138058	1.134834	1.131965	1.129395
1001	0.867525	0.951564				0.973105				0.893044	0.911799	0.923434	0.931357	0.937101	0.941455	0.94487	0.947619	0.949881	0.951774	0.953381	0.954764	0.955965	0.957018	0.95795	0.95878	0.959523	0.960193
1002	1.207935	1.060873				1.05766				1.309311	1.235802	1.195529	1.170108	1.152602	1.139813	1.13006	1.122377	1.116168	1.111046	1.106749	1.103092	1.099942	1.097201	1.094793	1.092662	1.090762	1.089058
1003	0.597496	0.816626				0.793735				0.470347	0.523688	0.561924	0.590675	0.613081	0.631034	0.645741	0.65801	0.6684	0.677313	0.685042	0.691809	0.697782	0.703094	0.707849	0.71213	0.716005	0.719528
1004	0.864501	0.950348				0.981785				0.925587	0.939025	0.947276	0.952858	0.956886	0.959929	0.962309	0.964222	0.965793	0.967105	0.968219	0.969176	0.970006	0.970734	0.971377	0.97195	0.972462	0.972924
